# Invasion Dynamics of a Fish-Free Landscape by Brown Trout (*Salmo trutta*)

**DOI:** 10.1371/journal.pone.0071052

**Published:** 2013-08-21

**Authors:** Jacques Labonne, Matthias Vignon, Etienne Prévost, Frédéric Lecomte, Julian J. Dodson, Renaud Kaeuffer, Jean-Christophe Aymes, Marc Jarry, Philippe Gaudin, Patrick Davaine, Edward Beall

**Affiliations:** 1 INRA, UMR 1224, ECOBIOP, Pôle d'Hydrobiologie, Saint-Pée sur Nivelle, France; 2 UPPA, UMR 1224, ECOBIOP, Pôle d'Hydrobiologie, Saint-Pée sur Nivelle, France; 3 Ministère des Ressources naturelles et de la Faune du Québec, Québec, Canada; 4 Université Laval, Département de Biologie, Pavillon Vachon, Québec, Canada; 5 Redpath Museum and Department of Biology, McGill University, Montreal, Quebec, Canada; Swansea University, United Kingdom

## Abstract

Metapopulation dynamics over the course of an invasion are usually difficult to grasp because they require large and reliable data collection, often unavailable. The invasion of the fish-free freshwater ecosystems of the remote sub-Antarctic Kerguelen Islands following man-made introductions of brown trout (*Salmo trutta*) in the 1950's is an exception to this rule. Benefiting from a full long term environmental research monitoring of the invasion, we built a Bayesian dynamic metapopulation model to analyze the invasion dynamics of 85 river systems over 51 years. The model accounted for patch size (river length and connections to lakes), alternative dispersal pathways between rivers, temporal trends in dynamics, and uncertainty in colonization date. The results show that the model correctly represents the observed pattern of invasion, especially if we assume a coastal dispersal pathway between patches. Landscape attributes such as patch size influenced the colonization function, but had no effect on propagule pressure. Independently from patch size and distance between patches, propagule pressure and colonization function were not constant through time. Propagule pressure increased over the course of colonization, whereas the colonization function decreased, conditional on propagule pressure. The resulting pattern of this antagonistic interplay is an initial rapid invasion phase followed by a strong decrease in the invasion rate. These temporal trends may be due to either adaptive processes or environmental gradients encountered along the colonization front. It was not possible to distinguish these two hypotheses. Because invasibility of Kerguelen Is. freshwater ecosystems is very high due to the lack of a pre-existing fish fauna and minimal human interference, our estimates of invasion dynamics represent a blueprint for the potential of brown trout invasiveness in pristine environments. Our conclusions shed light on the future of polar regions where, because of climate change, fish-free ecosystems become increasingly accessible to invasion by fish species.

## Introduction

Biological invasions are a major source of biodiversity loss and environmental disturbance. Coastal estuarine and marine ecosystems are currently among the most heavily invaded systems in the world, with alien organisms being a significant part of the biota [1], [2]. In invasion biology, the invasion success is intrinsically related to invasiveness (i.e. the intrinsic ability of species to become invasive) and invasibility (i.e. the susceptibility of communities to the establishment/proliferation of invaders, [3]). The competition-driven biotic resistance describes the ability of resident species in a community to reduce the success of exotic invasions, with the idea that invasibility decreases with an increase in species richness [4].

Unfortunately, the monitoring of most biological invasions suffers from two shortcomings. First, the initial conditions of invasions are generally poorly documented (numbers, origins, early dynamics, [5]), and the data series are generally insufficient. Second, a large number of factors generally interact with the invasion process: the multiple natural forces counteracting or resisting the successful implementation of the first settlers (*e.g.* niche packing, pre-existing fauna, predators), as well as human-mediated elements, either facilitating (e.g. disturbed habitat, altered fauna, etc.) or restricting the invasion processes (*e.g.* exploitation, control, containment, etc.). A direct consequence is that it is difficult if not impossible to disentangle these confounding factors from intraspecific evolutionary process of major interest which are known to happen sometimes on short temporal scales [6], [7], [8], [9]. High levels of biotic interactions in natural systems would normally preclude disentangling invasiveness from invasibility. Coincidentally, due to climate change, a variety of landscapes such as Arctic and Antarctic territories are or will be soon accessible to a wide range of species by natural or human-driven dispersal [10], [11], [12]: these landscapes usually shelter many under exploited ecological niches (high invasibility), in which the usual confounding factors do not exist, but for which our current knowledge will therefore not apply.

The brown trout (*Salmo trutta* L.) provides an interesting illustration of the above mentioned general context. In the northern hemisphere, brown trout has been shown to be modestly invasive, even when competing with other local salmoninae [12], [13], [14], [15]. When introduced in the southern hemisphere, the species rapidly proliferated in South America [16] and New Zealand [17]. For these invasions, there were established local fish communities that directly suffered from the invasion of brown trout. The pre-established community may even have promoted invasibility by providing food for the invading trout [18], [19], [20], [21]. While all of these invasions were documented, they do not fully help to forecast upcoming events in new, fish-free hydrographic systems liberated by melting ice caps in the polar regions and potentially available for colonization [22], [23], [24]. Yet the pace of invasion of these newly accessible freshwater bodies is of importance: salmonids are good candidates for colonizing under exploited ecological niches in these ecosystems [25], [26], [27], [28], [29]. They may interact with local species which have temporarily resisted the rapid ecological transition due to ice meltdown [30], they are known to modify their environment [31] and they may also represent a new resource for local human populations [23].

The introduction of brown trout in the 1950 s in subantarctic Kerguelen Is. presents an ideal opportunity to study an invasion process in a simplified context. The freshwater ecosystems of these islands were initially devoid of any fish species, thus providing the potential for many new ecological niches, and post-introduction fishing pressure was limited. We therefore are able to measure the invasiveness (combination of dispersal, demography and adaptation) of the species in a post-glacial landscape with barely any interaction with human activities. The brown trout was successfully introduced in 6 systems, was secondarily transported to another 3 systems and naturally colonized 32 new systems [32]. Natural colonization was achieved by anadromous individuals leaving an already colonized river and dispersing through the marine environment to reach a new river for reproduction. When natural reproduction occurred in a river (first observed in 1962 in two separate stocked systems), no local extinction was ever documented thereafter. While numerous attempts to explain invasion patterns generally suffer from largely anecdotal observations and *a posteriori* argumentation, here the progression and timing of colonization by brown trout of these fish-free ecosystems is fully documented.

We present a dynamic modeling approach to analyze the respective roles of spatial (arrangement of patches), temporal, and ecological (patch size) factors in the success of colonization by brown trout. The model is composed of a propagule pressure term and a colonization function term, that estimates the probability to become colonized conditional on a given level of propagule pressure. We asked the following questions: how does the spatial arrangement of patches control colonization? Can patch size play a role in propagule pressure or the colonization function? Do propagule pressure and colonization function change over time? Answering these questions serves to provide a blueprint for invasion dynamics in unexploited and ecologically unsaturated landscapes.

## Materials and Methods

### Species description

Brown trout is an iteroparous, facultative anadromous salmonid species, originating in the Eurasian region [33], that exhibits both a resident and a marine migratory form. Reproduction occurs only in freshwater habitats, but the growth phase can be achieved in either river, lake, estuarine or marine environments. This strategy can be viewed as an efficient way to use multiple resources, with a potential balance between costs and benefits for each phenotype. It also allows the species to easily colonize new watersheds via marine migration.

### Geographical and historical context

The Kerguelen Islands are located at the convergence of the Southern Ocean and Indian Ocean waters (69°30′E–49°30′S, Figure S1). They are composed of a mainland (surface  = 6675 km^2^) and more than 300 islands, many of which do not harbor significant freshwater hydrographic systems. The mainland itself is characterized by an environmental cline, where the eastern side is a mix of lowland plains and mountainous landscapes, with relatively dry weather (rainfall <500 mm per year). In contrast, the west is dominated by mountainous landscapes and glaciers, with heavy rainfall due to exposure to dominant marine winds (rainfall >2000 mm per year). On the one hand, the general dynamics of the landscape are affected by climate change: glaciers in the west are melting rapidly [34], [35], thereby creating new freshwater systems. On the other hand, the archipelago is undergoing a dramatic ecological change driven by the effects of introduced species (insects, plants, herbivorous mammals, cat, salmonids, [36], [37]). It was only in 1954 that salmonids were introduced in freshwater systems of Kerguelen Is. A species poor native invertebrate community is dominated by Chironomidae and zooplankters [38], [39], [40].

The freshwater systems in Kerguelen present a large variety of environmental conditions, from relatively warm waters (that may exceed 20°C during the southern summer) to very cold waters in mountain lakes and river systems located below glaciers. This gradient is correlated with a productivity gradient, but because many rivers have direct access to the sea, even some very oligotrophic systems are hospitable for anadromous species. Freshwater systems also vary in size, from large drainage areas (150 km^2^) connected to lake systems, to first order streams directly connected to the sea. Many systems are isolated by impassable waterfalls. Along with brown trout, seven other salmonid species were introduced in Kerguelen Is. Three species disappeared quickly, one is currently nearing extinction and the remaining three species are showing signs of stagnation or very recent and limited signs of expansion [32]. Co-existence with brown trout was limited: in five hydrographic systems, one other species was introduced simultaneously with brown trout. In three hydrographic systems, one other species of salmonid had previously been introduced before brown trout colonization but ecological saturation was far from reached [41]. A full data report on introductions (numbers, stages, origins) is available in Lecomte et al. [32].

### Data

Our analysis encompasses 85 contiguous river systems (hereafter referred to as patches) over 59 years (1954–2012). We define a patch as colonized when actual reproduction is ascertained. The first ascertained natural reproductions occurred in 1962, so the statistical inference of the natural dynamics of colonization are actually made over 51 years (Appendix S1). The monitoring of colonization has been traced back using historical reports, data (abundance estimates using electrofishing) and samples (detailed in Lecomte et al [32]). The monitoring zone increased with time, following the various introduction attempts (see File S1). Angling by personnel stationed at Kerguelen provided additional presence/absence data, notably for more distant sites.

The capture of juveniles indicated actual natural reproduction. The sole presence of anadromous adults in a patch was not considered as sufficient to conclude that the patch was colonized. When the time lag between two sampling campaigns was too long for some systems, we relied on age determination from scale and otolith samples of resident fish to infer the date of first natural reproduction of recently established local populations. However, population age structure was sometimes already balanced and therefore the first exact date of reproduction could not be inferred. We then accounted for uncertainty in colonization date, assuming that the probable first reproduction date ranged between our estimation using ageing of older resident fish, and our last previous sampling in the patch. Such uncertainty ranged from one to twenty years (median  = 6.5 years) and affected 24 of the 85 monitored patches (Appendix S1).

### Model description

We used a stochastic and dynamic metapopulation framework (see [42], [43], [44]), although no extinction was observed for the considered species in the Kerguelen Is. We therefore adapted the metapopulation model in discrete time by deleting the representation of the extinction process. We previously checked that it did not affect our conclusions.

Let *Y_t,i_* a Bernoulli variable describing the state of patch *i* at time *t*, 0 for uncolonized state and 1 for colonized state:
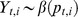

*p_t,i_* is the probability that an empty patch *i* is naturally colonized at time *t* conditional on the states of the *j* other patches at *t-1*such that:



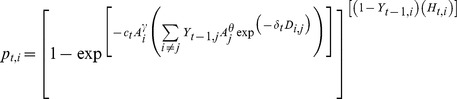
with *c_t_* and *δ_t_* the colonization and dispersal parameters, *A_i_* the size of the *i^th^* patch, *γ* the exponent describing the relationship between patch size and its colonization probability, *θ* the exponent describing the relationship between patch size and the quantity of emitted propagules, *H* an indicator variable specifying man made introduction (equals 0 when the patch has been artificially populated, 1 otherwise), and *D_ij_* the distance between patches *i* and *j*. The exponential of -*δ_t_D_ij_* is the dispersal kernel and controls for the probability that propagules emigrate over the distance *D* [45]. The summed term including the dispersal kernel, the state of patches *Y_t-1_* and their size effect A_j_ *^θ^* is referred to as propagule pressure on patch *i*. Finally, -*c_t_A_i_^γ^*is referred to as the colonization function (conditional on propagule pressure) hereafter: it controls the probability to become colonized conditional on a given level of propagule pressure. This model recursively accounts for the state of the patches beginning at the first time step, which is always known. Once a patch changes to the colonized state, it remains so (no extinction).

### Dispersal scenarios

We tested several hypotheses of dispersal pathways. This was done by providing different distance matrices between pairs of patches (*D_i,j_*). Four matrices were computed. First a distance-independent hypothesis was formulated with a distance matrix wherein all pairs of patches are equally distant. Second, a stepping-stone matrix was elaborated, where the distance between patches only depended on the relative positions of river mouths along the shoreline, where all contiguous patches are separated by a distance of 1 and distance between two non-contiguous patches is therefore equal to the number of patches encountered along a coastal pathway plus one. To do so, patches were numbered from 1 to 85 (see Figure S1) clockwise starting from the north-westernmost river to the south-westernmost. For rivers located on a different island (5 small rivers), they were integrated in this count as intermediate between the two nearest patches on the mainland (Appendix S2). Third, a matrix was calculated using the shortest path between two patches within a 5 km-wide coastal buffer: on Kerguelen Is., this allows to directly cross the eastern inner bay of Kerguelen, for example (Figure S1, Appendix S2). Finally, a fourth matrix was calculated by considering the shortest path between patches within a1 km-wide buffer along the coast, thus constraining dispersal to a coastal pathway, yet allowing the crossing of small fjords (i.e. width<1 km, Appendix S2).

### Patch size

Patch size is an important component of metapopulation models [45]. Here we classified patches in 6 ordered modalities depending on their size (main stem length) and their degree of connection with lakes. Small rivers (length<2.5 km) had a score of one, intermediate rivers a score of two (2.5<length<10 km) and large rivers a score of three (length>10 km). This first score was augmented when connection with lakes was present: one point was given for connections with small lakes or ponds (area<500ha), two points for a connection with a large lake (area>500ha), and three points for connections with a multi-lake system. This pseudo-logarithmic ranking synthesizes the overall carrying capacity of the system.

In the present model, we investigated whether patch size had an influence on propagule emission or on colonization function. To do so, the effect of the patch size *A* was controlled by two exponents (*θ* for propagule pressure, *γ* for colonization function). Positive values for *θ* indicate a trend for a large patch to emit more propagules, zero values indicate a lack of effect of patch size on propagule pressure, and a negative values imply that smaller patches emit more propagules than big ones. The same rationale applies for *γ* regarding the effect of patch size on colonization function conditional on propagule pressure.

### Time effect

We finally investigated whether the invasion dynamics were constant through time, by including a time effect on the *c* and/or *δ* parameters. Colonization function or propagule pressure could indeed change for several reasons, including climate change and/or trophic changes in freshwater or marine waters. A non-exclusive explanation could involve adaptation of brown trout to these new ecosystems and landscapes through the evolution of migratory traits [46]. The time effect on *c* and *δ* parameters was modeled as follows:
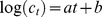


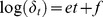

*b* and *f* are the intercept values when there is no time effect, and *a* and *e* are the slopes for the time effect.

### Parameter estimation and model selection

Statistical inference was conducted in the Bayesian framework. We used few informative priors for the model parameters. Uniform [−100,100] and independent prior distributions were assigned to *a*, *b*, *e* and *f*. *γ* and *θ* were given independent and uniform prior distributions but with a reduced span [−5, 5] given that such parameters are biologically unlikely to be large (large values of exponents would lead to an unreasonable number of propagules either attracted or released). The joint posterior distribution of all unknown quantities of the model, i.e. parameters and missing colonization data, was approximated by MCMC sampling as implemented by the OpenBUGS (version 3.21) software. A MCMC sample of 10000 draws was used, after checking its convergence by applying the Gelman-Rubin test [47].

To compare models according to dispersal hypotheses, we built our own goodness-of-fit criterion:
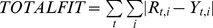



This criterion compares the states of the patches as predicted *a posteriori* by the model (*R_t,i_*) to their observed states (*Y_t,i_*). It equals zero for a perfect prediction, with a worst possible score of 4335 (51 years×85 patches). Its posterior distribution is easily obtained by MCMC sampling. It is then straight forward to calculate the percentage of patches for which the model accurately predicts the colonization state. For each MCMC draw of the model parameters, a full history of colonization is predicted and the criterion is computed according to the above formula. Note that the number of parameters in our models remains constant (n = 6) so this criterion compares equally parameterized models and we therefore do not need to use information criteria accounting for parameter number scaling. The model code is provided in Appendix S3.

## Results

### Parameter estimates

Among the four proposed dispersal matrices, the distance-independent model showed the poorest fit to data (median  = 949.5, see Table 1). Among the three other scenarios, the coastal migration model provided the best fit. We therefore used this distance matrix to explore the estimates of the model parameters. The posterior distribution of all the model parameters revealed a significant updating of the priors by the available data. The 95% posterior probability intervals were much narrower compared to the definition range of their priors, and all parameters were statistically different from zero, except *θ*. The patch size showed a positive effect on colonization function (median(*γ*)  = 1.355, Figure S2): largest patches were more likely to be colonized. Regarding propagule pressure, there was no clear effect of patch size although it seemed that small patches tended to release more propagules than large ones (median(*θ*)  = −0.387), but the opposite hypothesis could not be ruled out (P(*θ>0*)* = *0.249). The time dependent parameter for colonization function *a* was negative and different from zero (median(*a*)  = −0.2426, Figure S2): all else being equal, the patch colonization function decreased over time. However, the time dependent parameter for the dispersal kernel *e* was negative and different from zero (median(*e*)  = −0.1393, Figure S2): propagules tended to go farther at the end of the colonization process compared to the beginning.

**Table 1:** Goodness-of-fit measures.

Dispersal scenario	5% Quantile	Median	95% Quantile	Percentage of accurate prediction
Distance-independent	772.85	949.5	1206	79%
Stepping-stone	388.95	551.5	984.15	87.3%
Shortest path	394.95	520.5	723.1	88%
Coastal	**378.95**	**497**	**672.05**	**88.5%**

Summary of posterior distributions of Goodness-of-fit measures (5, 50 and 95% quantiles) for the four alternative dispersal scenarios, as well as the percentage of patches for which the model accurately predicts the colonization state. The lower values in bold indicate the best fit.

### Model fit and predictions

When plotted comparatively with the observed number of colonized rivers, the posterior predictions of the model showed large variations of invasion dynamics (Figure S3). This is partly due to the missing information regarding the colonization date of some patches. Yet, the model did not predict a lack of invasion or a total invasion (the number of colonized rivers is always above 25 and below 65). It also predicted the relative slowing in the number of colonized rivers in recent years. The median of the posterior check was located between the observed and the maximum number of colonized rivers.

We here provide simple posterior predictions based on a hypothetical situation: we assume a landscape with only two patches. One of them is colonized at time t = 0 (small patch, A = 1). We predict the probability that the second patch will be colonized (conditional on not being already colonized) depending on its size, distance to the other patch, and time. In any situation regarding patch size and geographic isolation, there is a maximum for colonization probability after approximately 30 years, whereas the probability is low at the beginning and low after 60 years (Figure S4). This is expected given the antagonistic time effects of dispersal and colonization parameters (*c* and *δ*). The peak in colonization probability depends on both patch size and distance. When patch size is increased, it has a positive effect on colonization probability. When distance between patches is increased, it has a negative effect on colonization probability, and it generates a delay in the colonization peak.

## Discussion

### Data and model fit

This study represents one of the best spatially explicit time series of presence/absence of an invasion process in vertebrates. Our long term monitoring provided a dynamic modeling of temporal and spatial variations rarely possible in many studies. Our direct integration of missing data also provided reliable confidence intervals around our estimates and predictions. Although we already knew that brown trout benefited from the quasi-absence of human exploitation to successfully colonize the Kerguelen Is. environment [46], the ecological (*i.e.* patch size in our case), spatial and temporal dynamics of the invasion process remained unclear. Although the spatial configuration and sizes of patches play a clear role, we also demonstrate that the colonization rate presents an underlying temporal dynamic. This result suggests that even in an unsaturated landscape, the colonization process has complex dynamics, and colonization rates are not constant in time and space. This may be related to either a changing environment through time and/or space not included in the model, or to demographic and evolutionary feedbacks. Nevertheless, this modeling exercise may be considered as an approximate blueprint of what could happen in other pristine environments in polar areas that may soon be accessible to salmonids due to global warming.

### Spatial context

The spatial configuration of the hydrographic systems of Kerguelen Is. has obviously played a role in the invasion process. The costal migration model provided the best fit, suggesting that fish usually stay close to the coast to disperse to new patches. This dispersal scenario intrinsically accounts for some important geographical features, such as the concavity and convexity of coast lines. Because of the 1 km buffer, some relatively complex concave regions can be easily crossed, such as the fjords within the inner Bay. Trout tend to remain near the shoreline, in specific habitats. Feeding and telemetric studies indeed indicate that anadromous brown trout feed chiefly in shallow waters close to the coast and are seldom found far offshore [48], [49], [50], [51]. Further isotopic and microchemistry analyses of scale and tissue samples could help to verify this hypothesis in the Kerguelen Is. Stepping-stone and shortest-path scenarios also provided acceptable fits to the data, showing that dispersal might also be influenced by other landscape attributes. This question could be further explored by changing the dispersal kernel [52], but it might interact very strongly with the distance matrix structure.

### Ecological context

The effect of patch size was dual. Patch size played a positive and clear role on the colonization function, which is somewhat expected in salmonids: larger systems tend to attract more propagules [16], yet it could also be due to higher freshwater survival for colonizers before spawning or for their offspring. Such a result is of interest for the invasion process, since it can possibly trigger colonization, despite the large distance to the nearest occupied patch. For instance, the south-western part of Kerguelen hosts many large, yet uncolonized, systems but that may strongly attract propagules or efficiently host colonizers. On the other hand, patch size had no clear effect on propagule release. The demographic lag not included in our model may generate an artifactual effect: indeed, large patches may not be saturated as quickly as small patches, and if propagule release depends on demographic saturation (*i.e.*, anadromy dependent on density), then we should expect such patterns at least temporarily. We could not include a demographic lag in the model, because it would require considering a full demographic model, not only a binary state approach, which is difficult – f not intractable- at such a large scale, although it has been attempted locally [53]. Alternatively, if patch size is correlated to patch quality, small patches may release more propagules than larger ones. Our measure of patch size includes connections to water bodies of various sizes and big patches can therefore encompass water bodies such as ponds and lakes that provide ample planktonic resources. They are thus favorable habitats for the resident life history tactic in Kerguelen. This may prevent the decision to migrate to sea from large patches, which is a prerequisite for actual dispersal. We also attempted to model the separate effects of river length and lake surface with two different parameters instead of aggregating them, but they could not be statistically differentiated in our data. Finally, brown trout was introduced with another species in some systems, but we did not include this effect so as to not overparameterize the model. It appears that over the period considered, there was no obvious difference in invasion rate in the vicinity of patches where trout occurred alone or in sympatry with another species.

### Temporal dynamics

The model highlights antagonistic time effects on the dispersal kernel and colonization function: dispersal seemed to increase with time, while the probability of colonization of a patch at a given level of propagule flow decreased with time. The resulting pattern of this interaction is that the colonization process accelerated during the first 30 years, and then appears to slow down independently of spatial configuration and patch size. Such a time effect could be correlated with an ecological gradient along the colonization front over the course of the invasion process. For instance, the western part of the archipelago is partly influenced by glaciers: some of the uncolonized patches on the current colonization front are directly linked to a glacier system. Such environments may be much harder to colonize due to the lack of trophic resources for juvenile fish. It was however not possible to integrate this effect in the model, because it was confounded with the time-related parameters. This environmental gradient may limit the invasion, but then again, approximately 21% of the ice cap surface in Kerguelen melted between 1963 and 2001 [35], and many other rivers free of ice are available, especially in the northern part of the archipelago. These observations thus cannot fully account for the reduced pace of invasion these last decades. As an alternative explanation regarding the time effect on dispersal kernel, Travis et al. [54], [55] have shown that dispersal could evolve as a byproduct of density-dependent demography during an invasion process. Life history traits related to dispersal are known to rapidly evolve in some cases [56], [57], [58] and are actually evolving relatively quickly in Kerguelen (unpublished data).

### Applied considerations

Here, in absence of native competitors and human exploitation, our data allowed us to focus exclusively on invasiveness of the brown trout, independently of the usual biotic controls and anthropogenic impacts. Our findings shed light on the future of the northern and southern hemispheres' hydrographic systems freed by melting ice caps, although such extrapolation demands caution. First, the invasion process might not be constant in space and time, independently from the spatial configuration of habitats. Demographic and evolutionary processes may play a role at least as important as geography. Second, we observed no case of patch extinction throughout the invasion process, even after more than 12 generations. No population went extinct after a first successful reproduction. This does not imply that extinction will not occur, but because habitat is distributed between fresh- and saltwater for this species, and because sea migration is facultative, extinction may be rare in relatively pristine environments compared to human-driven extinction processes in the original distribution area. The limitation of prey availability to brown trout may not be as strong as initially expected based on closely related species [17]. Still, in light of ongoing climate change, investigating the range expansion of anadromous salmonids should also accommodate physiological and behavioural considerations as they can have direct and substantial impacts on their dynamics [59], [60], [61], [26], [62]. Third, the geographical context nevertheless plays an important role: concave and complex coastlines may favor invasion by brown trout. This result may allow targeting areas more likely to be successfully invaded first, either to prevent the invasion to protect local ecosystems (since the invasion process itself may affect ecosystem trophic structure [31]), or to facilitate fisheries exploitation for local human communities.

The whole Kerguelen archipelago is now a Natural Reserve, and the progress of invasive species is of prime interest for biodiversity management. Based on our present results, it is interesting to witness how the invasion has slowed. About 40% of the archipelago is already colonized, and we are monitoring the progress on the colonization front. If our quantitative predictions are accurate, the remainder of the invasion should take much longer than the initial spread. It remains to be investigated what the most likely factors influencing the dynamics of colonization are: environmental variation along the colonization front, evolutionary processes, or both.

## Supporting Information

Figure S1 **Map of Kerguelen Islands.** Map of Kerguelen Is. showing sampling locations included in the model and their current status (adapted from Lecomte et al. 2013).
(TIF)

Figure S2 **Model parameter estimates.** Posterior density for *a*, *b*, *e*, *f*, *θ* and *γ* hyper-parameters (MCMC sample size  = 10000). *a* and *b* parameters represent the colonization function (log(*c_t_*) = *at*+*b*), e and f parameters represent the dispersal function(log(*δ*) = *et*+*f*), and *θ* and*γ*are the exponents for the effect of patch size *A* on attraction and emission respectively.
(TIF)

Figure S3 **Prediction of invasion dynamics.** Dynamics of invasion: number of colonized patches against time. The grey lines represent simulated trajectories drawn from the model a posteriori (N = 1000), the full black line shows observed number of colonized patches, the interrupted black line represents the maximum number of colonized patches (*i.e.* assuming that all the patches in an unknown state were actually colonized).The actual but partly unknown colonization history falls between these two thick black lines.
(TIF)

Figure S4 **Colonization probability as a function of distance, patch size, and time.** Predicted probability that a small single colonized patch (A = 1) colonizes another single empty patch of size A = 1, 3 or 6 and located at various distances (light grey for 5 km, grey for 10 km, dark grey for 20 km), as a function of time. Solid lines represent the median of the posterior density; the lower and upper interrupted lines represent 5 and 95% quantiles of the distribution for each considered distance.
(TIF)

File S1 **Mapped dynamics of observed colonization over the last five decades in the Kerguelen Islands.** Empty circles represent uncolonized rivers, full black circle represent colonized rivers. The number in the upper right corner of each map is the number of colonized patch compared to the total number of considered patch for this study.
(TIF)

Appendix S1 **River colonization data in Kerguelen Islands.**
(PDF)

Appendix S2 **Distance matrices between hydrographic systems for each dispersal scenario.** Appendix 2a: distances calculated with a stepping-stone approach (stepping-stone dispersal). Appendix 2b: distances calculated within a 1 km buffer from the coast (coastal dispersal). Appendix 3b: distances calculated within a 5 km buffer from the coast (shortest path dispersal).
(PDF)

Appendix S3 **Model code for the Openbugs 3.21 software.**
(PDF)
