# Supplementary figures and images for: Invasion Dynamics of a Fish-Free Landscape by Brown Trout (Salmo trutta)
Source: PLoS One. 2013 Aug 21;8(8):e71052. doi: 10.1371/journal.pone.0071052 (PMC3749212; doi:10.1371/journal.pone.0071052)

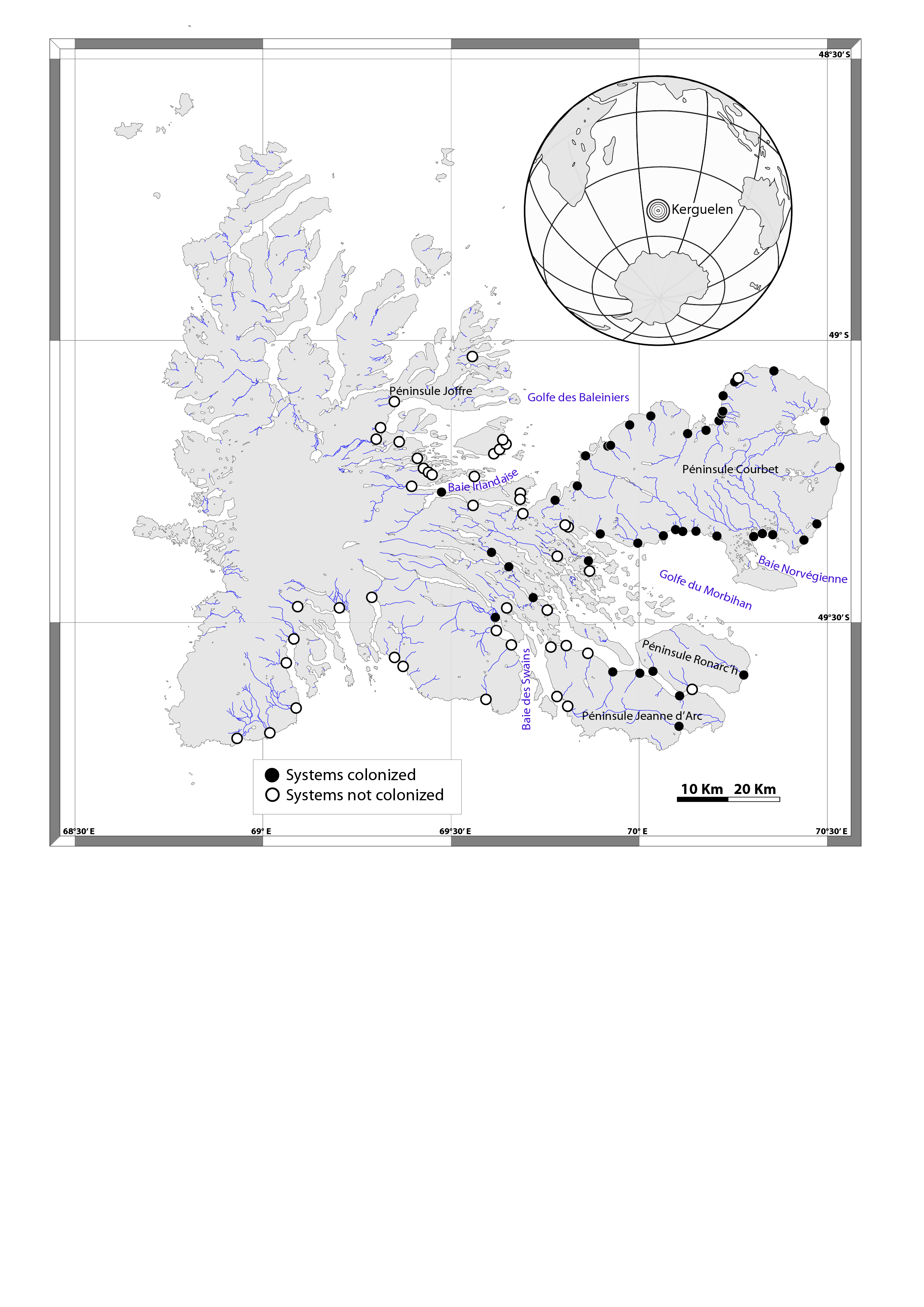

Supplement: Figure S1 — Map of Kerguelen Islands. Map of Kerguelen Is. showing sampling locations included in the model and their current status (adapted from Lecomte et al. 2013). (TIF) [file pone.0071052.s001.tif]

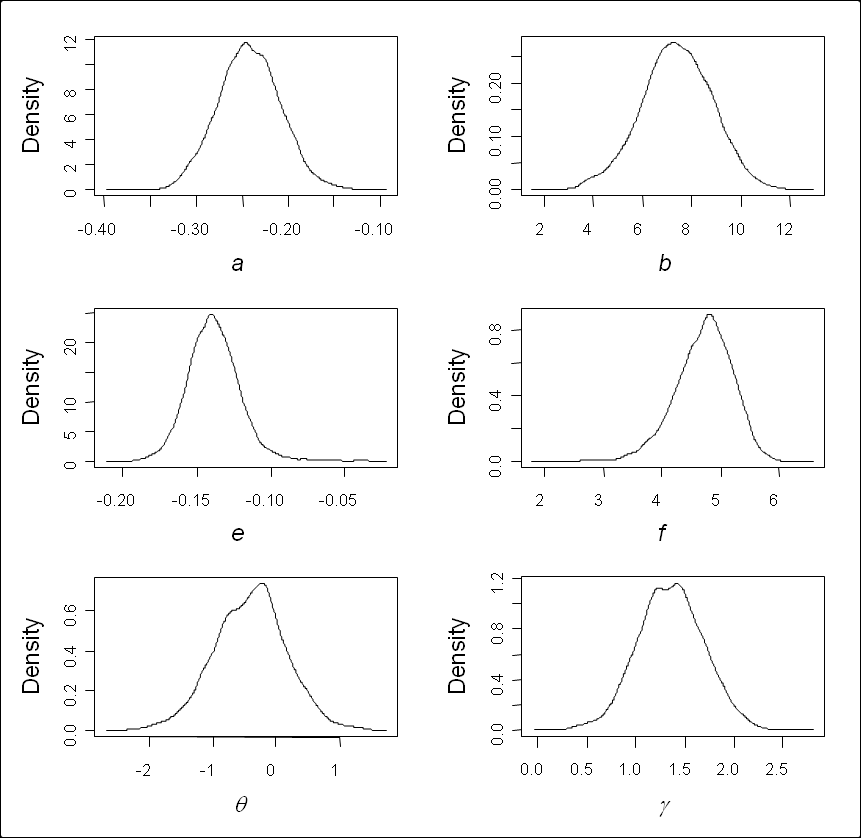

Supplement: Figure S2 — Model parameter estimates. Posterior density for a, b, e, f, θ and γ hyper-parameters (MCMC sample size = 10000). a and b parameters represent the colonization function (log(ct) = at+b), e and f parameters represent the dispersal function(log(δ) = et+f), and θ andγare the exponents for the effect of patch size A on attraction and emission respectively. (TIF) [file pone.0071052.s002.tif]

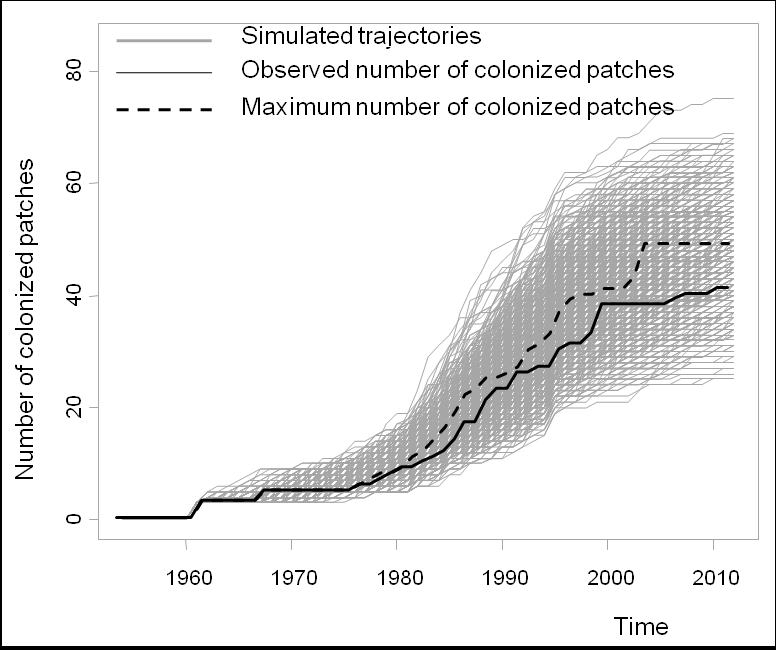

Supplement: Figure S3 — Prediction of invasion dynamics. Dynamics of invasion: number of colonized patches against time. The grey lines represent simulated trajectories drawn from the model a posteriori (N = 1000), the full black line shows observed number of colonized patches, the interrupted black line represents the maximum number of colonized patches (i.e. assuming that all the patches in an unknown state were actually colonized).The actual but partly unknown colonization history falls between these two thick black lines. (TIF) [file pone.0071052.s003.tif]

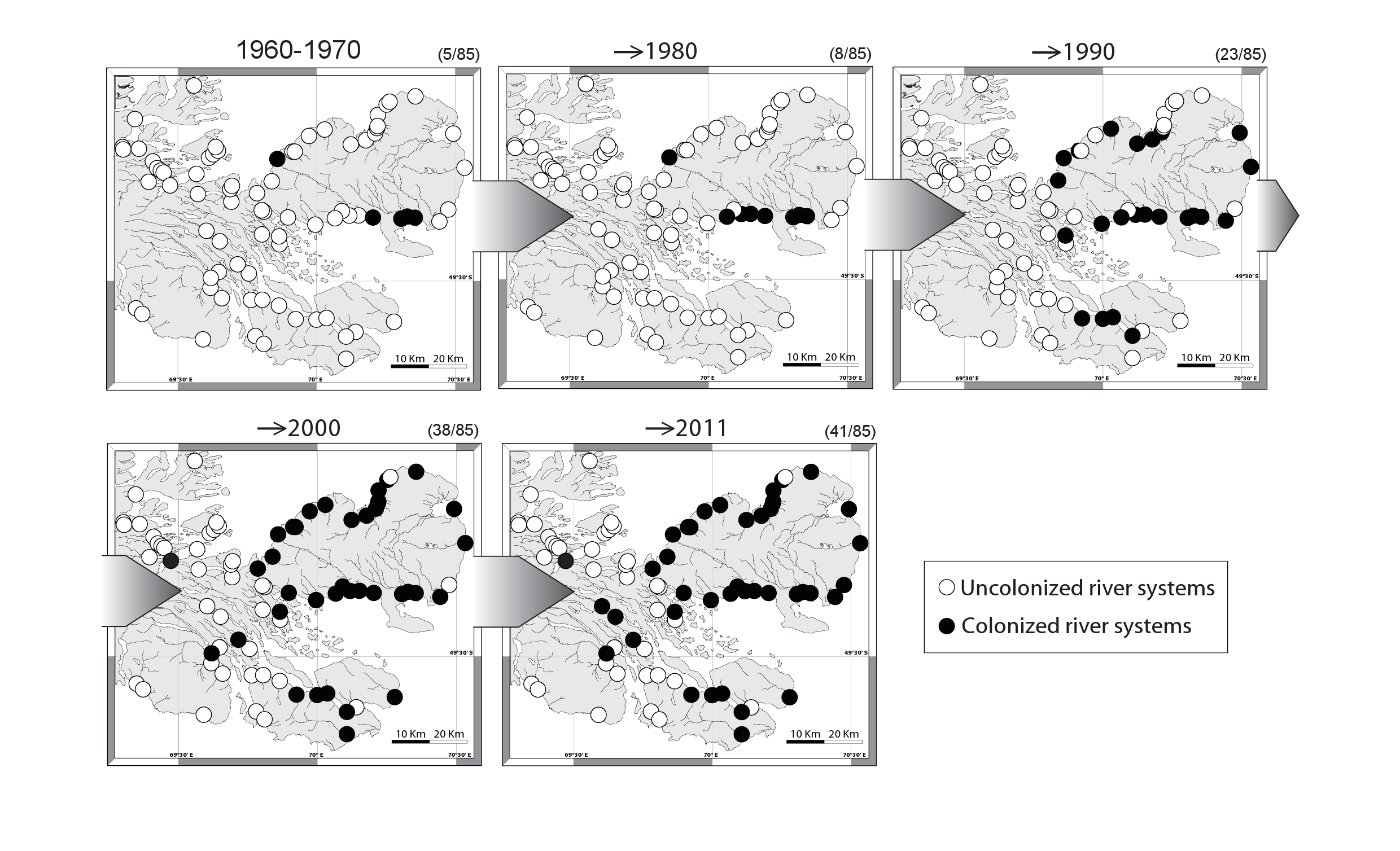

Supplement: File S1 — Mapped dynamics of observed colonization over the last five decades in the Kerguelen Islands. Empty circles represent uncolonized rivers, full black circle represent colonized rivers. The number in the upper right corner of each map is the number of colonized patch compared to the total number of considered patch for this study. (TIF) [file pone.0071052.s005.tif]
