# Supplementary material for: Invasion Dynamics of a Fish-Free Landscape by Brown Trout (Salmo trutta)
Source: PLoS One. 2013 Aug 21;8(8):e71052. doi: 10.1371/journal.pone.0071052 (PMC3749212; doi:10.1371/journal.pone.0071052)
Supplement: Appendix S1 — River colonization data in Kerguelen Islands. (PDF) [file pone.0071052.s006.pdf]

	River Name	Entrelacs R.	Baie Chanzy	Déversoir du Lac de la Croix du Sud	Outlet of Lac Euphrosine	Ballon R.	Sans Nom R.	S. Brook Ile aux Skuas	Brook 1 Ile du Port	Brook 2 Ile du Port	Brook 3 Ile du Port	Brook 4 Ile du Port	Val Froide R.	Val Travers R.	Brook N. of Port-Couvreux	Brook of Port-Couvreux cabin	Brook W end of Havre du Beau Temps	Anse Sablonneuse Brook	Port Kirk R.	Val de l'Ouest R.	Studer R.	Doute R.	Charbon R.	Sérail R.	Chasseurs R.	Nord R.	Pépins R.	Cataractes R.	Hautes Mares South Brook (Gorfous 1)	Hautes Mares North Brook (Gorfous 2)	Pointe des Cabanes Brook (Gorfous 4)	Cap Rouge Brook (Gorfous 5)	N. Cap Rouge Brook (Gorfous 6)	Cap de Rohan R.	Lac Marville Outlet	Manchots R.	Calcédoines R.	Bungay R.	Albatros R.	Norvégienne R.	Château R.	PAF Ferme R.	Borgne R.	Sud R.	Pointe de l'épave Brook	Américains R.	3 Lacs R.	Val d'Auge R.	Anse de St Malo R.	Basin downstream of Korrigans Lake	Baie de l'Observatoire R.	SW Brook on Bras Jules Laboureur	Grisanche R.	Lac des Fougères Outlet	Lac d'Armor Basin	Anse J. Bourcart Brook	Ravin du Charbon R.	Vallée des Neiges Basin	Acaena R.	Vallée de la Planchette R.	Val Raide Brook	Mouettes R.	Macaronis R.	Vallée Phonolite Basin	Val du Levant R.	Anse de la Canicule R.	Crête des Laves R.	Halage des Swains R.	Northern arm of Anse aux Ecueils Brook	La Clarée Basin: Hermance Lake & Galets R.	Olsen R.	Vallée du Radioleine R.	Val des Skuas R.	Doigt de Sainte-Anne R.	Plaine de Dante R.	Mont Ventoux R.	Mont Sinaï R.	Bastille R.	Lac Mariot outlet	Plaine d'Ampère R.	Portes Noires R.	Contacts R.	Mouche R.	Larmor R.	Plage Jaune R.	Sables R.
	Patch number																																																																																			
Time step	Year	1	2	3	4	5	6	7	8	9	10	11	12	13	14	15	16	17	18	19	20	21	22	23	24	25	26	27	28	29	30	31	32	33	34	35	36	37	38	39	40	41	42	43	44	45	46	47	48	49	50	51	52	53	54	55	56	57	58	59	60	61	62	63	64	65	66	67	68	69	70	71	72	73	74	75	76	77	78	79	80	81	82	83	84	85
1	1954	0	0	0	0	0	0	0	0	0	0	0	0	0	0	0	0	0	0	0	0	0	0	0	0	0	0	0	0	0	0	0	0	0	0	0	0	0	0	0	0	0	0	0	0	0	0	0	0	0	0	0	0	0	0	0	0	0	0	0	0	0	0	0	0	0	0	0	0	0	0	0	0	0	0	0	0	0	0	0	0	0	0	0	0	0
2	1955	0	0	0	0	0	0	0	0	0	0	0	0	0	0	0	0	0	0	0	0	0	0	0	0	0	0	0	0	0	0	0	0	0	0	0	0	0	0	0	0	0	0	0	0	0	0	0	0	0	0	0	0	0	0	0	0	0	0	0	0	0	0	0	0	0	0	0	0	0	0	0	0	0	0	0	0	0	0	0	0	0	0	0	0	0
3	1956	0	0	0	0	0	0	0	0	0	0	0	0	0	0	0	0	0	0	0	0	0	0	0	0	0	0	0	0	0	0	0	0	0	0	0	0	0	0	0	0	0	0	0	0	0	0	0	0	0	0	0	0	0	0	0	0	0	0	0	0	0	0	0	0	0	0	0	0	0	0	0	0	0	0	0	0	0	0	0	0	0	0	0	0	0
4	1957	0	0	0	0	0	0	0	0	0	0	0	0	0	0	0	0	0	0	0	0	0	0	0	0	0	0	0	0	0	0	0	0	0	0	0	0	0	0	0	0	0	0	0	0	0	0	0	0	0	0	0	0	0	0	0	0	0	0	0	0	0	0	0	0	0	0	0	0	0	0	0	0	0	0	0	0	0	0	0	0	0	0	0	0	0
5	1958	0	0	0	0	0	0	0	0	0	0	0	0	0	0	0	0	0	0	0	0	0	0	0	0	0	0	0	0	0	0	0	0	0	0	0	0	0	0	0	0	0	0	0	0	0	0	0	0	0	0	0	0	0	0	0	0	0	0	0	0	0	0	0	0	0	0	0	0	0	0	0	0	0	0	0	0	0	0	0	0	0	0	0	0	0
6	1959	0	0	0	0	0	0	0	0	0	0	0	0	0	0	0	0	0	0	0	0	0	0	0	0	0	0	0	0	0	0	0	0	0	0	0	0	0	0	0	0	0	0	0	0	0	0	0	0	0	0	0	0	0	0	0	0	0	0	0	0	0	0	0	0	0	0	0	0	0	0	0	0	0	0	0	0	0	0	0	0	0	0	0	0	0
7	1960	0	0	0	0	0	0	0	0	0	0	0	0	0	0	0	0	0	0	0	0	0	0	0	0	0	0	0	0	0	0	0	0	0	0	0	0	0	0	0	0	0	0	0	0	0	0	0	0	0	0	0	0	0	0	0	0	0	0	0	0	0	0	0	0	0	0	0	0	0	0	0	0	0	0	0	0	0	0	0	0	0	0	0	0	0
8	1961	0	0	0	0	0	0	0	0	0	0	0	0	0	0	0	0	0	0	0	0	0	0	0	0	0	0	0	0	0	0	0	0	0	0	0	0	0	0	0	0	0	0	0	0	0	0	0	0	0	0	0	0	0	0	0	0	0	0	0	0	0	0	0	0	0	0	0	0	0	0	0	0	0	0	0	0	0	0	0	0	0	0	0	0	0
9	1962	0	0	0	0	0	0	0	0	0	0	0	0	0	0	0	0	0	0	0	1	0	0	0	0	0	0	0	0	0	0	0	0	0	0	0	0	0	0	0	1	1	0	0	0	0	0	0	0	0	0	0	0	0	0	0	0	0	0	0	0	0	0	0	0	0	0	0	0	0	0	0	0	0	0	0	0	0	0	0	0	0	0	0	0	0
10	1963	0	0	0	0	0	0	0	0	0	0	0	0	0	0	0	0	0	0	0	1	0	0	0	0	0	0	0	0	0	0	0	0	0	0	0	0	0	0	0	1	1	0	0	0	0	0	0	0	0	0	0	0	0	0	0	0	0	0	0	0	0	0	0	0	0	0	0	0	0	0	0	0	0	0	0	0	0	0	0	0	0	0	0	0	0
11	1964	0	0	0	0	0	0	0	0	0	0	0	0	0	0	0	0	0	0	0	1	0	0	0	0	0	0	0	0	0	0	0	0	0	0	0	0	0	0	0	1	1	0	0	0	0	0	0	0	0	0	0	0	0	0	0	0	0	0	0	0	0	0	0	0	0	0	0	0	0	0	0	0	0	0	0	0	0	0	0	0	0	0	0	0	0
12	1965	0	0	0	0	0	0	0	0	0	0	0	0	0	0	0	0	0	0	0	1	0	0	0	0	0	0	0	0	0	0	0	0	0	0	0	0	0	0	0	1	1	0	0	0	0	0	0	0	0	0	0	0	0	0	0	0	0	0	0	0	0	0	0	0	0	0	0	0	0	0	0	0	0	0	0	0	0	0	0	0	0	0	0	0	0
13	1966	0	0	0	0	0	0	0	0	0	0	0	0	0	0	0	0	0	0	0	1	0	0	0	0	0	0	0	0	0	0	0	0	0	0	0	0	0	0	0	1	1	0	0	0	0	0	0	0	0	0	0	0	0	0	0	0	0	0	0	0	0	0	0	0	0	0	0	0	0	0	0	0	0	0	0	0	0	0	0	0	0	0	0	0	0
14	1967	0	0	0	0	0	0	0	0	0	0	0	0	0	0	0	0	0	0	0	1	0	0	0	0	0	0	0	0	0	0	0	0	0	0	0	0	0	0	0	1	1	0	0	0	0	0	0	0	0	0	0	0	0	0	0	0	0	0	0	0	0	0	0	0	0	0	0	0	0	0	0	0	0	0	0	0	0	0	0	0	0	0	0	0	0
15	1968	0	0	0	0	0	0	0	0	0	0	0	0	0	0	0	0	0	0	0	1	0	0	0	0	0	0	0	0	0	0	0	0	0	0	0	0	0	1	1	1	1	0	0	0	0	0	0	0	0	0	0	0	0	0	0	0	0	0	0	0	0	0	0	0	0	0	0	0	0	0	0	0	0	0	0	0	0	0	0	0	0	0	0	0	0
16	1969	0	0	0	0	0	0	0	0	0	0	0	0	0	0	0	0	0	0	0	1	0	0	0	0	0	0	0	0	0	0	0	0	0	0	0	0	0	1	1	1	1	0	0	0	0	0	0	0	0	0	0	0	0	0	0	0	0	0	0	0	0	0	0	0	0	0	0	0	0	0	0	0	0	0	0	0	0	0	0	0	0	0	0	0	0
17	1970	0	0	0	0	0	0	0	0	0	0	0	0	0	0	0	0	0	0	0	1	0	0	0	0	0	0	0	0	0	0	0	0	0	0	0	0	0	1	1	1	1	0	0	0	0	0	0	0	0	0	0	0	0	0	0	0	0	0	0	0	0	0	0	0	0	0	0	0	0	0	0	0	0	0	0	0	0	0	0	0	0	0	0	0	0
18	1971	0	0	0	0	0	0	0	0	0	0	0	0	0	0	0	0	0	0	0	1	0	0	0	0	0	0	0	0	0	0	0	0	0	0	0	0	0	1	1	1	1	0	0	0	0	0	0	0	0	0	0	0	0	0	0	0	0	0	0	0	0	0	0	0	0	0	0	0	0	0	0	0	0	0	0	0	0	0	0	0	0	0	0	0	0
19	1972	0	0	0	0	0	0	0	0	0	0	0	0	0	0	0	0	0	0	0	1	0	0	0	0	0	0	0	0	0	0	0	0	0	0	0	0	0	1	1	1	1	0	0	0	0	0	0	0	0	0	0	0	0	0	0	0	0	0	0	0	0	0	0	0	0	0	0	0	0	0	0	0	0	0	0	0	0	0	0	0	0	0	0	0	0
20	1973	0	0	0	0	0	0	0	0	0	0	0	0	0	0	0	0	0	0	0	1	0	0	0	0	0	0	0	0	0	0	0	0	0	0	0	0	0	1	1	1	1	0	0	0	0	0	0	0	0	0	0	0	0	0	0	0	0	0	0	0	0	0	0	0	0	0	0	0	0	0	0	0	0	0	0	0	0	0	0	0	0	0	0	0	0
21	1974	0	0	0	0	0	0	0	0	0	0	0	0	0	0	0	0	0	0	0	1	0	0	0	0	0	0	0	0	0	0	0	0	0	0	0	0	0	1	1	1	1	0	0	0	0	0	0	0	0	0	0	0	0	0	0	0	0	0	0	0	0	0	0	0	0	0	0	0	0	0	0	0	0	0	0	0	0	0	0	0	0	0	0	0	0
22	1975	0	0	0	0	0	0	0	0	0	0	0	0	0	0	0	0	0	0	0	1	0	0	0	0	0	0	0	0	0	0	0	0	0	0	0	0	0	1	1	1	1	0	0	0	0	0	0	0	0	0	0	0	0	0	0	0	0	0	0	0	0	0	0	0	0	0	0	0	0	0	0	0	0	0	0	0	0	0	0	0	0	0	0	0	0
23	1976	0	0	0	0	0	0	0	0	0	0	0	0	0	0	0	0	0	0	0	1	0	0	0	0	0	0	0	0	0	0	0	0	0	0	0	0	0	1	1	1	1	0	0	0	0	0	0	0	0	0	0	0	0	0	0	0	0	0	0	0	0	0	0	0	0	0	0	0	0	0	0	0	0	0	0	0	0	0	0	0	0	0	0	0	0
24	1977	0	0	0	0	0	0	0	0	0	0	0	0	0	0	0	0	0	0	0	1	0	0	0	0	0	0	0	0	0	0	0	0	0	0	0	0	0	1	1	1	1	1	0	0	0	0	0	0	0	0	0	0	0	0	0	0	0	0	0	0	0	0	0	0	0	0	0	0	0	0	0	0	0	0	0	0	0	0	0	0	0	0	0	0	0
25	1978	0	0	0	0	0	0	0	0	0	0	0	0	0	0	0	0	0	0	0	1	0	0	0	0	0	0	0	0	0	0	0	0	0	0	0	0	0	1	1	1	1	1	0	0	NA	0	0	0	0	0	0	0	0	0	0	0	0	0	0	0	0	0	0	0	0	0	0	0	0	0	0	0	0	0	0	0	0	0	0	0	0	0	0	0	0
26	1979	0	0	0	0	0	0	0	0	0	0	0	0	0	0	0	0	0	0	0	1	0	0	0	0	0	0	0	0	0	0	0	0	0	0	0	0	0	1	1	1	1	1	1	0	NA	0	0	0	0	0	0	0	0	0	0	0	0	0	0	0	0	0	0	0	0	0	0	0	0	0	0	0	0	0	0	0	0	0	0	0	0	0	0	0	0
27	1980	0	0	0	0	0	0	0	0	0	0	0	0	0	0	0	0	0	0	0	1	0	0	0	0	0	0	0	0	0	0	0	0	0	0	0	0	0	1	1	1	1	1	1	0	1	0	0	0	0	0	0	0	0	0	0	0	0	0	0	0	0	0	0	0	0	0	0	0	0	0	0	0	0	0	0	0	0	0	0	0	0	0	0	0	0
28	1981	0	0	0	0	0	0	0	0	0	0	0	0	0	0	0	0	0	0	0	1	0	0	0	0	0	0	0	0	0	0	0	0	0	0	0	0	0	1	1	1	1	1	1	0	1	0	0	0	1	0	0	0	0	0	0	0	0	0	0	0	0	0	0	0	0	0	0	0	0	0	0	0	0	0	0	0	0	0	0	0	0	0	0	0	0
29	1982	0	0	0	0	0	0	0	0	0	0	0	0	0	0	0	0	0	0	0	1	0	0	0	0	0	0	0	0	0	0	0	0	0	NA	NA	0	0	1	1	1	1	1	1	0	1	0	0	0	1	0	0	0	0	0	0	0	0	0	0	0	0	0	0	0	0	0	0	0	0	0	0	0	0	0	0	0	0	0	0	0	0	0	0	0	0
30	1983	0	0	0	0	0	0	0	0	0	0	0	0	0	0	0	0	0	0	0	1	0	0	0	0	0	0	0	0	0	0	0	0	0	NA	NA	0	0	1	1	1	1	1	1	0	1	0	0	0	1	0	0	0	0	0	0	0	0	1	0	0	0	0	0	0	0	0	0	0	0	0	0	0	0	0	0	0	0	0	0	0	0	0	0	0	0
31	1984	0	0	0	0	0	0	0	0	0	0	0	0	0	0	0	0	0	0	0	1	0	0	0	0	0	0	0	0	0	0	0	0	0	NA	NA	0	NA	1	1	1	1	1	1	0	1	0	0	0	1	0	0	0	0	0	0	0	0	1	1	0	0	0	0	0	0	0	0	0	0	0	0	0	0	0	0	0	0	0	0	0	0	0	0	0	0
32	1985	0	0	0	0	0	0	0	0	0	0	0	0	0	0	0	0	0	0	0	1	1	0	0	0	0	0	NA	0	0	0	0	0	0	NA	NA	0	NA	1	1	1	1	1	1	0	1	0	0	0	1	0	0	0	0	0	0	0	0	1	1	0	0	0	0	0	0	0	0	0	0	0	0	0	0	0	0	0	0	0	0	0	0	0	0	0	0
33	1986	0	0	0	0	0	0	0	0	0	0	0	0	0	0	0	0	0	0	0	1	1	NA	0	1	1	0	NA	0	0	0	0	0	0	NA	NA	0	NA	1	1	1	1	1	1	0	1	0	0	0	1	0	0	0	0	0	0	0	0	1	1	0	0	0	0	0	0	0	0	0	0	0	0	0	0	0	0	0	0	0	0	0	0	0	0	0	0
34	1987	0	0	0	0	0	0	0	0	0	0	0	0	0	0	0	0	0	0	1	1	1	NA	0	1	1	0	NA	0	0	0	0	0	0	NA	NA	0	NA	1	1	1	1	1	1	0	1	0	0	0	1	0	0	0	0	0	0	0	0	1	1	1	1	0	0	0	0	0	0	0	0	0	0	0	0	0	0	0	0	0	0	0	0	0	0	0	0
35	1988	0	0	0	0	0	0	0	0	0	0	0	0	0	0	0	0	0	0	1	1	1	NA	NA	1	1	0	NA	0	0	0	0	0	0	NA	NA	0	NA	1	1	1	1	1	1	0	1	0	0	0	1	0	0	0	0	0	0	0	0	1	1	1	1	0	0	0	0	0	0	0	0	0	0	0	0	0	0	0	0	0	0	0	0	0	0	0	0
36	1989	0	0	0	0	0	0	0	0	0	0	0	0	0	0	0	0	0	0	1	1	1	NA	NA	1	1	1	1	0	0	0	0	0	0	NA	NA	0	1	1	1	1	1	1	1	0	1	1	0	0	1	0	0	0	0	0	0	0	0	1	1	1	1	0	0	0	0	0	0	0	0	0	0	0	0	0	0	0	0	0	0	0	0	0	0	0	0
37	1990	0	0	0	0	0	0	0	0	0	0	0	0	0	0	0	0	0	0	1	1	1	NA	NA	1	1	1	1	0	0	0	0	0	0	1	1	0	1	1	1	1	1	1	1	0	1	1	0	0	1	0	0	0	0	0	0	0	0	1	1	1	1	0	0	0	0	0	0	0	0	0	0	0	0	0	0	0	0	0	0	0	0	0	0	0	0
38	1991	0	0	0	0	0	0	0	0	0	0	0	0	0	0	0	0	0	0	1	1	1	NA	NA	1	1	1	1	0	NA	0	0	0	0	1	1	0	1	1	1	1	1	1	1	0	1	1	0	0	1	0	0	0	0	0	0	0	0	1	1	1	1	0	0	0	0	0	0	0	0	0	0	0	0	0	0	0	0	0	0	0	0	0	0	0	0
39	1992	0	0	0	0	0	0	0	0	0	0	0	0	0	0	0	0	0	0	1	1	1	1	1	1	1	1	1	0	NA	0	0	0	0	1	1	0	1	1	1	1	1	1	1	1	1	1	0	0	1	0	0	0	0	0	0	0	0	1	1	1	1	0	0	0	0	0	0	0	0	0	0	0	0	0	0	0	0	0	0	0	0	0	0	0	0
40	1993	0	0	0	0	0	0	0	0	0	0	0	0	0	0	0	0	0	0	1	1	1	1	1	1	1	1	1	0	NA	0	0	0	0	1	1	0	1	1	1	1	1	1	1	1	1	1	0	0	1	0	0	0	0	0	0	0	0	1	1	1	1	0	0	0	NA	NA	NA	0	0	0	0	0	0	0	0	0	0	0	0	0	0	0	0	0	0
41	1994	0	0	0	0	0	0	0	0	0	0	0	0	0	0	0	0	0	0	1	1	1	1	1	1	1	1	1	0	NA	0	0	0	0	1	1	0	1	1	1	1	1	1	1	1	1	1	0	0	1	0	0	0	0	1	0	0	0	1	1	1	1	0	0	0	NA	NA	NA	0	0	0	0	0	0	0	0	0	0	0	0	0	0	0	0	0	0
42	1995	0	0	0	0	0	0	0	0	0	0	0	0	0	0	0	0	0	0	1	1	1	1	1	1	1	1	1	0	NA	0	0	0	NA	1	1	0	1	1	1	1	1	1	1	1	1	1	NA	0	1	0	0	0	0	1	0	0	0	1	1	1	1	0	0	0	NA	NA	NA	0	0	0	0	0	0	0	0	0	0	0	0	0	0	0	0	0	0
43	1996	0	0	0	0	0	0	0	0	0	0	0	0	1	0	0	0	0	0	1	1	1	1	1	1	1	1	1	0	NA	0	0	0	NA	1	1	0	1	1	1	1	1	1	1	1	1	1	NA	0	1	0	0	0	0	1	0	0	0	1	1	1	1	NA	0	1	NA	NA	NA	0	1	0	0	0	0	0	0	0	0	0	0	0	0	0	0	0	0
44	1997	0	0	0	0	0	0	0	0	0	0	0	0	1	0	0	0	0	1	1	1	1	1	1	1	1	1	1	0	NA	0	NA	0	NA	1	1	0	1	1	1	1	1	1	1	1	1	1	NA	0	1	0	0	0	0	1	0	0	0	1	1	1	1	NA	0	1	NA	NA	NA	0	1	0	0	0	0	0	0	0	0	0	0	0	0	0	0	0	0
45	1998	0	0	0	0	0	0	0	0	0	0	0	0	1	0	0	0	0	1	1	1	1	1	1	1	1	1	1	0	NA	NA	NA	0	NA	1	1	0	1	1	1	1	1	1	1	1	1	1	NA	0	1	0	0	0	0	1	0	0	0	1	1	1	1	NA	0	1	NA	NA	NA	0	1	0	0	0	0	0	0	0	0	0	0	0	0	0	0	0	0
46	1999	0	0	0	0	0	0	0	0	0	0	0	0	1	0	0	0	0	1	1	1	1	1	1	1	1	1	1	0	NA	NA	NA	0	NA	1	1	0	1	1	1	1	1	1	1	1	1	1	1	0	1	0	0	0	0	1	0	0	0	1	1	1	1	1	0	1	NA	NA	NA	0	1	0	0	0	0	0	0	0	0	0	0	0	0	0	0	0	0
47	2000	0	0	0	0	0	0	0	0	0	0	0	0	1	0	0	0	0	1	1	1	1	1	1	1	1	1	1	1	1	1	1	0	1	1	1	0	1	1	1	1	1	1	1	1	1	1	1	0	1	0	0	0	0	1	0	0	0	1	1	1	1	1	0	1	NA	NA	NA	0	1	0	0	0	0	0	0	0	0	0	0	0	0	0	0	0	0
48	2001	0	0	0	0	0	0	0	0	0	0	0	0	1	0	0	0	0	1	1	1	1	1	1	1	1	1	1	1	1	1	1	0	1	1	1	0	1	1	1	1	1	1	1	1	1	1	1	0	1	0	0	0	0	1	0	0	0	1	1	1	1	1	0	1	NA	NA	NA	0	1	0	0	0	0	0	0	0	0	0	0	0	0	0	0	0	0
49	2002	0	0	0	0	0	0	0	0	0	0	0	0	1	0	0	0	0	1	1	1	1	1	1	1	1	1	1	1	1	1	1	0	1	1	1	0	1	1	1	1	1	1	1	1	1	1	1	0	1	0	0	0	0	1	0	0	0	1	1	1	1	1	0	1	NA	NA	NA	0	1	0	0	0	0	0	0	0	0	0	0	0	0	0	0	0	0
50	2003	0	0	0	0	0	0	0	0	0	0	0	0	1	0	0	0	0	1	1	1	1	1	1	1	1	1	1	1	1	1	1	0	1	1	1	NA	1	1	1	1	1	1	1	1	1	1	1	NA	1	0	0	0	0	1	0	0	0	1	1	1	1	1	0	1	NA	NA	NA	0	1	0	0	0	0	0	0	0	0	0	0	0	0	0	0	0	0
51	2004	0	0	0	0	0	0	0	0	0	0	0	0	1	0	0	0	0	1	1	1	1	1	1	1	1	1	1	1	1	1	1	0	1	1	1	NA	1	1	1	1	1	1	1	1	1	1	1	NA	1	NA	0	NA	NA	1	NA	NA	NA	1	1	1	1	1	0	1	NA	NA	NA	0	1	0	0	0	0	0	0	0	0	0	0	0	0	0	0	0	0
52	2005	0	0	0	0	0	0	0	0	0	0	0	0	1	0	0	0	0	1	1	1	1	1	1	1	1	1	1	1	1	1	1	0	1	1	1	NA	1	1	1	1	1	1	1	1	1	1	1	NA	1	NA	0	NA	NA	1	NA	NA	NA	1	1	1	1	1	0	1	NA	NA	NA	0	1	0	0	0	0	0	0	0	0	0	0	0	0	0	0	0	0
53	2006	0	0	0	0	0	0	0	0	0	0	0	0	1	0	0	0	0	1	1	1	1	1	1	1	1	1	1	1	1	1	1	0	1	1	1	NA	1	1	1	1	1	1	1	1	1	1	1	NA	1	NA	0	NA	NA	1	NA	NA	NA	1	1	1	1	1	0	1	NA	NA	NA	0	1	0	0	0	0	0	0	0	0	0	0	0	0	0	0	0	0
54	2007	0	0	0	0	0	0	0	0	0	0	0	0	1	0	0	0	0	1	1	1	1	1	1	1	1	1	1	1	1	1	1	0	1	1	1	1	1	1	1	1	1	1	1	1	1	1	1	NA	1	NA	0	NA	NA	1	NA	NA	NA	1	1	1	1	1	0	1	NA	NA	NA	0	1	0	0	0	0	0	0	0	0	0	0	0	0	0	0	0	0
55	2008	0	0	0	0	0	0	0	0	0	0	0	0	1	0	0	0	0	1	1	1	1	1	1	1	1	1	1	1	1	1	1	0	1	1	1	1	1	1	1	1	1	1	1	1	1	1	1	NA	1	NA	0	1	NA	1	NA	NA	NA	1	1	1	1	1	0	1	NA	NA	NA	0	1	0	0	0	0	0	0	0	0	0	0	0	0	0	0	0	0
56	2009	0	0	0	0	0	0	0	0	0	0	0	0	1	0	0	0	0	1	1	1	1	1	1	1	1	1	1	1	1	1	1	0	1	1	1	1	1	1	1	1	1	1	1	1	1	1	1	NA	1	NA	0	1	NA	1	NA	NA	NA	1	1	1	1	1	0	1	NA	NA	NA	0	1	0	0	0	0	0	0	0	0	0	0	0	0	0	0	0	0
57	2010	0	0	0	0	0	0	0	0	0	0	0	0	1	0	0	0	0	1	1	1	1	1	1	1	1	1	1	1	1	1	1	0	1	1	1	1	1	1	1	1	1	1	1	1	1	1	1	NA	1	NA	0	1	NA	1	NA	NA	NA	1	1	1	1	1	0	1	NA	NA	NA	0	1	0	0	0	0	0	0	0	0	0	0	0	0	0	0	0	0
58	2011	0	0	0	0	0	0	0	0	0	0	0	0	1	0	0	0	0	1	1	1	1	1	1	1	1	1	1	1	1	1	1	0	1	1	1	1	1	1	1	1	1	1	1	1	1	1	1	NA	1	NA	0	1	1	1	NA	NA	NA	1	1	1	1	1	0	1	NA	NA	NA	0	1	0	0	0	0	0	0	0	0	0	0	0	0	0	0	0	0
59	2012	0	0	0	0	0	0	0	0	0	0	0	0	1	0	0	0	0	1	1	1	1	1	1	1	1	1	1	1	1	1	1	0	1	1	1	1	1	1	1	1	1	1	1	1	1	1	1	NA	1	NA	0	1	1	1	NA	NA	NA	1	1	1	1	1	0	1	NA	NA	NA	0	1	0	0	0	0	0	0	0	0	0	0	0	0	0	0	0	0
Introduction		F	F	F	F	F	F	F	F	F	F	F	F	T	F	F	F	F	F	F	T	F	F	F	F	T	F	F	F	F	F	F	F	F	F	F	F	F	F	F	T	T	F	F	F	F	F	F	F	F	F	F	F	F	T	F	F	F	T	F	F	F	F	F	T	F	F	F	F	T	F	F	F	F	F	F	F	F	F	F	F	F	F	F	F	F
Patch size		3	1	3	3	1	1	1	1	1	1	1	2	4	1	1	1	1	2	2	6	1	1	1	2	2	3	1	1	2	1	1	1	1	6	4	2	3	3	3	3	1	2	2	1	2	1	1	1	5	2	1	5	2	5	1	1	1	2	2	1	1	3	2	2	1	1	1	1	4	2	2	1	2	2	1	1	1	4	4	2	2	3	3	3	3

Labonne et al. 2013, Appendix 1: river colonization data in Kerguelen Is
