# Supplementary material for: Invasion Dynamics of a Fish-Free Landscape by Brown Trout (Salmo trutta)
Source: PLoS One. 2013 Aug 21;8(8):e71052. doi: 10.1371/journal.pone.0071052 (PMC3749212; doi:10.1371/journal.pone.0071052)
Supplement: Appendix S3 — Model code for the Openbugs 3.21 software. (PDF) [file pone.0071052.s008.pdf]

```
# OpenBugs code for colonization model from Labonne et al. 2013.
# Invasion dynamics of an empty and unexploited ecological niche by brown trout (Salmo trutta).

model {
# Likelihood
	for (t in 2:T) {								# Iteration on time
		for (i in 1:N) {							# Iteration on patches
			Y[t,i]~dbern(p[t,i])					# Bernouilli process for colonization state probability p
			p[t,i]<-pow(pcol[t,i],col[t,i])			# If the exponent "col" is worth 0, then p=1.
			pcol[t,i]<-1-exp(-10*c[t]*(pow(A[i],gamma))*sigma[t,i])	# Inference on colonization probability.
			col[t,i]<-(1-Y[t-1,i])*(V[t,i])			# V indicates the previous state of the patch - or manmade introduction.
			sigma[t,i] <- sum(out[t,i,])/N			# sum of propagule flow on patch i over emitting patches j = propagule pressure
		}
		log(c[t])<- a*t+b							# Time effect on colonization function
	}
	# progule flow computation
	for (t in 2:T) {
		for (i in 1:N) {							# Iteration on focal patches where propagules arrive
			for (j in 1:N) {						# Iteration on patches that emit propagules
				out[t,i,j]<-(Y[t-1,j]*(pow(A[j],theta))*exp(-delta[t]*(D[i,j]/10)))	# Propagule pressure on patch i from patch j
			}
		}
		log(delta[t])<-e*t+f						# Time effect on dispersal kernel
	}
# Priors on hyper parameters
	a ~ dunif(-100,100)		## non informative prior
	b ~ dunif(-100,100)		## non informative prior
	e ~ dunif(-100,100)		## non informative prior
	f ~ dunif(-100,100)		## non informative prior
	gamma ~dunif(-5,5)			## gamma is an exponent : reduced prior range.
	theta ~ dunif(-5,5)		## theta is an exponent : reduced prior range.
}
```
