# Supplementary material for: Invasion Dynamics of a Fish-Free Landscape by Brown Trout (Salmo trutta)
Source: PLoS One. 2013 Aug 21;8(8):e71052. doi: 10.1371/journal.pone.0071052 (PMC3749212; doi:10.1371/journal.pone.0071052)
Supplement: Appendix S2 — Distance matrices between hydrographic systems for each dispersal scenario. Appendix 2a: distances calculated with a stepping-stone approach (stepping-stone dispersal). Appendix 2b: distances calculated within a 1 km buffer from the coast (coastal dispersal). Appendix 3b: distances calculated within a 5 km buffer from the coast (shortest path dispersal). (PDF) [file pone.0071052.s007.pdf]

Patch names	Entrelacs R.	Baie Chanzy	Déversoir du Lac de la Croix du Sud	Outlet of Lac Euphrosine	Ballon R.	Sans Nom R.	S. Brook Ile aux Skuas	Brook 1 Ile du Port	Brook 2 Ile du Port	Brook 3 Ile du Port	Brook 4 Ile du Port	Val Froide R.	Val Travers R.	Brook N. of Port-Couvreux	Brook of Port-Couvreux cabin	Brook W end of Havre du Beau Temps	Anse Sablonneuse Brook	Port Kirk R.	Val de l'Ouest R.	Studer R.	Doute R.	Charbon R.	Sérail R.	Chasseurs R.	Nord R.	Pépins R.	Cataractes R.	Hautes Mares South Brook (Gorfous 1)	Hautes Mares North Brook (Gorfous 2)	Pointe des Cabanes Brook (Gorfous 4)	Cap Rouge Brook (Gorfous 5)	N. Cap Rouge Brook (Gorfous 6)	Cap de Rohan R.	Lac Marville Outlet	Manchots R.	Calcédoines R.	Bungay R.	Albatros R.	Norvégienne R.	Château R.	PAF Ferme R.	Borgne R.	Sud R.	Pointe de l'épave Brook	Américains R.	3 Lacs R.	Val d'Auge R.	Anse de St Malo R.	Basin downstream of Korrigans Lake	Baie de l'Observatoire R.	SW Brook on Bras Jules Laboureur	Grisanche R.	Lac des Fougères Outlet	Lac d'Armor Basin	Anse J. Bourcart Brook	Ravin du Charbon R.	Vallée des Neiges Basin	Acaena R.	Vallée de la Planchette R.	Val Raide Brook	Mouettes R.	Macaronis R.	Vallée Phonolite Basin	Val du Levant R.	Anse de la Canicule R.	Crête des Laves R.	Halage des Swains R.	Northern arm of Anse aux Ecueils Brook	La Clarée Basin: Hermance Lake & Galets R.	Olsen R.	Vallée du Radioleine R.	Val des Skuas R.	Doigt de Sainte-Anne R.	Plaine de Dante R.	Mont Ventoux R.	Mont Sinaï R.	Bastille R.	Lac Mariot outlet	Plaine d'Ampère R.	Portes Noires R.	Contacts R.	Mouche R.	Larmor R.	Plage Jaune R.	Sables R.
Entrelacs R.	0	1	2	3	4	5	6	7	8	9	10	11	12	13	14	15	16	17	18	19	20	21	22	23	24	25	26	27	28	29	30	31	32	33	34	35	36	37	38	39	40	41	42	43	44	45	46	47	48	49	50	51	52	53	54	55	56	57	58	59	60	61	62	63	64	65	66	67	68	69	70	71	72	73	1	2	3	74	75	76	77	78	79	80	81
Baie Chanzy	1	0	1	2	3	4	5	6	7	8	9	10	11	12	13	14	15	16	17	18	19	20	21	22	23	24	25	26	27	28	29	30	31	32	33	34	35	36	37	38	39	40	41	42	43	44	45	46	47	48	49	50	51	52	53	54	55	56	57	58	59	60	61	62	63	64	65	66	67	68	69	70	71	72	2	3	4	73	74	75	76	77	78	79	80
Déversoir du Lac de la Croix du Sud	2	1	0	1	2	3	4	5	6	7	8	9	10	11	12	13	14	15	16	17	18	19	20	21	22	23	24	25	26	27	28	29	30	31	32	33	34	35	36	37	38	39	40	41	42	43	44	45	46	47	48	49	50	51	52	53	54	55	56	57	58	59	60	61	62	63	64	65	66	67	68	69	70	71	3	4	5	72	73	74	75	76	77	78	79
Outlet of Lac Euphrosine	3	2	1	0	1	2	3	4	5	6	7	8	9	10	11	12	13	14	15	16	17	18	19	20	21	22	23	24	25	26	27	28	29	30	31	32	33	34	35	36	37	38	39	40	41	42	43	44	45	46	47	48	49	50	51	52	53	54	55	56	57	58	59	60	61	62	63	64	65	66	67	68	69	70	4	5	6	71	72	73	74	75	76	77	78
Ballon R.	4	3	2	1	0	1	2	3	4	5	6	7	8	9	10	11	12	13	14	15	16	17	18	19	20	21	22	23	24	25	26	27	28	29	30	31	32	33	34	35	36	37	38	39	40	41	42	43	44	45	46	47	48	49	50	51	52	53	54	55	56	57	58	59	60	61	62	63	64	65	66	67	68	69	5	6	7	70	71	72	73	74	75	76	77
Sans Nom R.	5	4	3	2	1	0	1	2	3	4	5	6	7	8	9	10	11	12	13	14	15	16	17	18	19	20	21	22	23	24	25	26	27	28	29	30	31	32	33	34	35	36	37	38	39	40	41	42	43	44	45	46	47	48	49	50	51	52	53	54	55	56	57	58	59	60	61	62	63	64	65	66	67	68	6	7	8	69	70	71	72	73	74	75	76
S. Brook Ile aux Skuas	6	5	4	3	2	1	0	1	2	3	4	5	6	7	8	9	10	11	12	13	14	15	16	17	18	19	20	21	22	23	24	25	26	27	28	29	30	31	32	33	34	35	36	37	38	39	40	41	42	43	44	45	46	47	48	49	50	51	52	53	54	55	56	57	58	59	60	61	62	63	64	65	66	67	7	8	9	68	69	70	71	72	73	74	75
Brook 1 Ile du Port	7	6	5	4	3	2	1	0	1	2	3	4	5	6	7	8	9	10	11	12	13	14	15	16	17	18	19	20	21	22	23	24	25	26	27	28	29	30	31	32	33	34	35	36	37	38	39	40	41	42	43	44	45	46	47	48	49	50	51	52	53	54	55	56	57	58	59	60	61	62	63	64	65	66	8	9	10	67	68	69	70	71	72	73	74
Brook 2 Ile du Port	8	7	6	5	4	3	2	1	0	1	2	3	4	5	6	7	8	9	10	11	12	13	14	15	16	17	18	19	20	21	22	23	24	25	26	27	28	29	30	31	32	33	34	35	36	37	38	39	40	41	42	43	44	45	46	47	48	49	50	51	52	53	54	55	56	57	58	59	60	61	62	63	64	65	9	10	11	66	67	68	69	70	71	72	73
Brook 3 Ile du Port	9	8	7	6	5	4	3	2	1	0	1	2	3	4	5	6	7	8	9	10	11	12	13	14	15	16	17	18	19	20	21	22	23	24	25	26	27	28	29	30	31	32	33	34	35	36	37	38	39	40	41	42	43	44	45	46	47	48	49	50	51	52	53	54	55	56	57	58	59	60	61	62	63	64	10	11	12	65	66	67	68	69	70	71	72
Brook 4 Ile du Port	10	9	8	7	6	5	4	3	2	1	0	1	2	3	4	5	6	7	8	9	10	11	12	13	14	15	16	17	18	19	20	21	22	23	24	25	26	27	28	29	30	31	32	33	34	35	36	37	38	39	40	41	42	43	44	45	46	47	48	49	50	51	52	53	54	55	56	57	58	59	60	61	62	63	11	12	13	64	65	66	67	68	69	70	71
Val Froide R.	11	10	9	8	7	6	5	4	3	2	1	0	1	2	3	4	5	6	7	8	9	10	11	12	13	14	15	16	17	18	19	20	21	22	23	24	25	26	27	28	29	30	31	32	33	34	35	36	37	38	39	40	41	42	43	44	45	46	47	48	49	50	51	52	53	54	55	56	57	58	59	60	61	62	12	13	14	63	64	65	66	67	68	69	70
Val Travers R.	12	11	10	9	8	7	6	5	4	3	2	1	0	1	2	3	4	5	6	7	8	9	10	11	12	13	14	15	16	17	18	19	20	21	22	23	24	25	26	27	28	29	30	31	32	33	34	35	36	37	38	39	40	41	42	43	44	45	46	47	48	49	50	51	52	53	54	55	56	57	58	59	60	61	13	14	15	62	63	64	65	66	67	68	69
Brook N. of Port-Couvreux	13	12	11	10	9	8	7	6	5	4	3	2	1	0	1	2	3	4	5	6	7	8	9	10	11	12	13	14	15	16	17	18	19	20	21	22	23	24	25	26	27	28	29	30	31	32	33	34	35	36	37	38	39	40	41	42	43	44	45	46	47	48	49	50	51	52	53	54	55	56	57	58	59	60	14	15	16	61	62	63	64	65	66	67	68
Brook of Port-Couvreux cabin	14	13	12	11	10	9	8	7	6	5	4	3	2	1	0	1	2	3	4	5	6	7	8	9	10	11	12	13	14	15	16	17	18	19	20	21	22	23	24	25	26	27	28	29	30	31	32	33	34	35	36	37	38	39	40	41	42	43	44	45	46	47	48	49	50	51	52	53	54	55	56	57	58	59	15	16	17	60	61	62	63	64	65	66	67
Brook W end of Havre du Beau Temps	15	14	13	12	11	10	9	8	7	6	5	4	3	2	1	0	1	2	3	4	5	6	7	8	9	10	11	12	13	14	15	16	17	18	19	20	21	22	23	24	25	26	27	28	29	30	31	32	33	34	35	36	37	38	39	40	41	42	43	44	45	46	47	48	49	50	51	52	53	54	55	56	57	58	16	17	18	59	60	61	62	63	64	65	66
Anse Sablonneuse Brook	16	15	14	13	12	11	10	9	8	7	6	5	4	3	2	1	0	1	2	3	4	5	6	7	8	9	10	11	12	13	14	15	16	17	18	19	20	21	22	23	24	25	26	27	28	29	30	31	32	33	34	35	36	37	38	39	40	41	42	43	44	45	46	47	48	49	50	51	52	53	54	55	56	57	17	18	19	58	59	60	61	62	63	64	65
Port Kirk R.	17	16	15	14	13	12	11	10	9	8	7	6	5	4	3	2	1	0	1	2	3	4	5	6	7	8	9	10	11	12	13	14	15	16	17	18	19	20	21	22	23	24	25	26	27	28	29	30	31	32	33	34	35	36	37	38	39	40	41	42	43	44	45	46	47	48	49	50	51	52	53	54	55	56	18	19	20	57	58	59	60	61	62	63	64
Val de l'Ouest R.	18	17	16	15	14	13	12	11	10	9	8	7	6	5	4	3	2	1	0	1	2	3	4	5	6	7	8	9	10	11	12	13	14	15	16	17	18	19	20	21	22	23	24	25	26	27	28	29	30	31	32	33	34	35	36	37	38	39	40	41	42	43	44	45	46	47	48	49	50	51	52	53	54	55	19	20	21	56	57	58	59	60	61	62	63
Studer R.	19	18	17	16	15	14	13	12	11	10	9	8	7	6	5	4	3	2	1	0	1	2	3	4	5	6	7	8	9	10	11	12	13	14	15	16	17	18	19	20	21	22	23	24	25	26	27	28	29	30	31	32	33	34	35	36	37	38	39	40	41	42	43	44	45	46	47	48	49	50	51	52	53	54	20	21	22	55	56	57	58	59	60	61	62
Doute R.	20	19	18	17	16	15	14	13	12	11	10	9	8	7	6	5	4	3	2	1	0	1	2	3	4	5	6	7	8	9	10	11	12	13	14	15	16	17	18	19	20	21	22	23	24	25	26	27	28	29	30	31	32	33	34	35	36	37	38	39	40	41	42	43	44	45	46	47	48	49	50	51	52	53	21	22	23	54	55	56	57	58	59	60	61
Charbon R.	21	20	19	18	17	16	15	14	13	12	11	10	9	8	7	6	5	4	3	2	1	0	1	2	3	4	5	6	7	8	9	10	11	12	13	14	15	16	17	18	19	20	21	22	23	24	25	26	27	28	29	30	31	32	33	34	35	36	37	38	39	40	41	42	43	44	45	46	47	48	49	50	51	52	22	23	24	53	54	55	56	57	58	59	60
Sérail R.	22	21	20	19	18	17	16	15	14	13	12	11	10	9	8	7	6	5	4	3	2	1	0	1	2	3	4	5	6	7	8	9	10	11	12	13	14	15	16	17	18	19	20	21	22	23	24	25	26	27	28	29	30	31	32	33	34	35	36	37	38	39	40	41	42	43	44	45	46	47	48	49	50	51	23	24	25	52	53	54	55	56	57	58	59
Chasseurs R.	23	22	21	20	19	18	17	16	15	14	13	12	11	10	9	8	7	6	5	4	3	2	1	0	1	2	3	4	5	6	7	8	9	10	11	12	13	14	15	16	17	18	19	20	21	22	23	24	25	26	27	28	29	30	31	32	33	34	35	36	37	38	39	40	41	42	43	44	45	46	47	48	49	50	24	25	26	51	52	53	54	55	56	57	58
Nord R.	24	23	22	21	20	19	18	17	16	15	14	13	12	11	10	9	8	7	6	5	4	3	2	1	0	1	2	3	4	5	6	7	8	9	10	11	12	13	14	15	16	17	18	19	20	21	22	23	24	25	26	27	28	29	30	31	32	33	34	35	36	37	38	39	40	41	42	43	44	45	46	47	48	49	25	26	27	50	51	52	53	54	55	56	57
Pépins R.	25	24	23	22	21	20	19	18	17	16	15	14	13	12	11	10	9	8	7	6	5	4	3	2	1	0	1	2	3	4	5	6	7	8	9	10	11	12	13	14	15	16	17	18	19	20	21	22	23	24	25	26	27	28	29	30	31	32	33	34	35	36	37	38	39	40	41	42	43	44	45	46	47	48	26	27	28	49	50	51	52	53	54	55	56
Cataractes R.	26	25	24	23	22	21	20	19	18	17	16	15	14	13	12	11	10	9	8	7	6	5	4	3	2	1	0	1	2	3	4	5	6	7	8	9	10	11	12	13	14	15	16	17	18	19	20	21	22	23	24	25	26	27	28	29	30	31	32	33	34	35	36	37	38	39	40	41	42	43	44	45	46	47	27	28	29	48	49	50	51	52	53	54	55
Hautes Mares South Brook (Gorfous 1)	27	26	25	24	23	22	21	20	19	18	17	16	15	14	13	12	11	10	9	8	7	6	5	4	3	2	1	0	1	2	3	4	5	6	7	8	9	10	11	12	13	14	15	16	17	18	19	20	21	22	23	24	25	26	27	28	29	30	31	32	33	34	35	36	37	38	39	40	41	42	43	44	45	46	28	29	30	47	48	49	50	51	52	53	54
Hautes Mares North Brook (Gorfous 2)	28	27	26	25	24	23	22	21	20	19	18	17	16	15	14	13	12	11	10	9	8	7	6	5	4	3	2	1	0	1	2	3	4	5	6	7	8	9	10	11	12	13	14	15	16	17	18	19	20	21	22	23	24	25	26	27	28	29	30	31	32	33	34	35	36	37	38	39	40	41	42	43	44	45	29	30	31	46	47	48	49	50	51	52	53
Pointe des Cabanes Brook (Gorfous 4)	29	28	27	26	25	24	23	22	21	20	19	18	17	16	15	14	13	12	11	10	9	8	7	6	5	4	3	2	1	0	1	2	3	4	5	6	7	8	9	10	11	12	13	14	15	16	17	18	19	20	21	22	23	24	25	26	27	28	29	30	31	32	33	34	35	36	37	38	39	40	41	42	43	44	30	31	32	45	46	47	48	49	50	51	52
Cap Rouge Brook (Gorfous 5)	30	29	28	27	26	25	24	23	22	21	20	19	18	17	16	15	14	13	12	11	10	9	8	7	6	5	4	3	2	1	0	1	2	3	4	5	6	7	8	9	10	11	12	13	14	15	16	17	18	19	20	21	22	23	24	25	26	27	28	29	30	31	32	33	34	35	36	37	38	39	40	41	42	43	31	32	33	44	45	46	47	48	49	50	51
N. Cap Rouge Brook (Gorfous 6)	31	30	29	28	27	26	25	24	23	22	21	20	19	18	17	16	15	14	13	12	11	10	9	8	7	6	5	4	3	2	1	0	1	2	3	4	5	6	7	8	9	10	11	12	13	14	15	16	17	18	19	20	21	22	23	24	25	26	27	28	29	30	31	32	33	34	35	36	37	38	39	40	41	42	32	33	34	43	44	45	46	47	48	49	50
Cap de Rohan R.	32	31	30	29	28	27	26	25	24	23	22	21	20	19	18	17	16	15	14	13	12	11	10	9	8	7	6	5	4	3	2	1	0	1	2	3	4	5	6	7	8	9	10	11	12	13	14	15	16	17	18	19	20	21	22	23	24	25	26	27	28	29	30	31	32	33	34	35	36	37	38	39	40	41	33	34	35	42	43	44	45	46	47	48	49
Lac Marville Outlet	33	32	31	30	29	28	27	26	25	24	23	22	21	20	19	18	17	16	15	14	13	12	11	10	9	8	7	6	5	4	3	2	1	0	1	2	3	4	5	6	7	8	9	10	11	12	13	14	15	16	17	18	19	20	21	22	23	24	25	26	27	28	29	30	31	32	33	34	35	36	37	38	39	40	34	35	36	41	42	43	44	45	46	47	48
Manchots R.	34	33	32	31	30	29	28	27	26	25	24	23	22	21	20	19	18	17	16	15	14	13	12	11	10	9	8	7	6	5	4	3	2	1	0	1	2	3	4	5	6	7	8	9	10	11	12	13	14	15	16	17	18	19	20	21	22	23	24	25	26	27	28	29	30	31	32	33	34	35	36	37	38	39	35	36	37	40	41	42	43	44	45	46	47
Calcédoines R.	35	34	33	32	31	30	29	28	27	26	25	24	23	22	21	20	19	18	17	16	15	14	13	12	11	10	9	8	7	6	5	4	3	2	1	0	1	2	3	4	5	6	7	8	9	10	11	12	13	14	15	16	17	18	19	20	21	22	23	24	25	26	27	28	29	30	31	32	33	34	35	36	37	38	36	37	38	39	40	41	42	43	44	45	46
Bungay R.	36	35	34	33	32	31	30	29	28	27	26	25	24	23	22	21	20	19	18	17	16	15	14	13	12	11	10	9	8	7	6	5	4	3	2	1	0	1	2	3	4	5	6	7	8	9	10	11	12	13	14	15	16	17	18	19	20	21	22	23	24	25	26	27	28	29	30	31	32	33	34	35	36	37	37	38	39	38	39	40	41	42	43	44	45
Albatros R.	37	36	35	34	33	32	31	30	29	28	27	26	25	24	23	22	21	20	19	18	17	16	15	14	13	12	11	10	9	8	7	6	5	4	3	2	1	0	1	2	3	4	5	6	7	8	9	10	11	12	13	14	15	16	17	18	19	20	21	22	23	24	25	26	27	28	29	30	31	32	33	34	35	36	38	39	40	37	38	39	40	41	42	43	44
Norvégienne R.	38	37	36	35	34	33	32	31	30	29	28	27	26	25	24	23	22	21	20	19	18	17	16	15	14	13	12	11	10	9	8	7	6	5	4	3	2	1	0	1	2	3	4	5	6	7	8	9	10	11	12	13	14	15	16	17	18	19	20	21	22	23	24	25	26	27	28	29	30	31	32	33	34	35	39	40	41	36	37	38	39	40	41	42	43
Château R.	39	38	37	36	35	34	33	32	31	30	29	28	27	26	25	24	23	22	21	20	19	18	17	16	15	14	13	12	11	10	9	8	7	6	5	4	3	2	1	0	1	2	3	4	5	6	7	8	9	10	11	12	13	14	15	16	17	18	19	20	21	22	23	24	25	26	27	28	29	30	31	32	33	34	40	41	42	35	36	37	38	39	40	41	42
PAF Ferme R.	40	39	38	37	36	35	34	33	32	31	30	29	28	27	26	25	24	23	22	21	20	19	18	17	16	15	14	13	12	11	10	9	8	7	6	5	4	3	2	1	0	1	2	3	4	5	6	7	8	9	10	11	12	13	14	15	16	17	18	19	20	21	22	23	24	25	26	27	28	29	30	31	32	33	41	42	43	34	35	36	37	38	39	40	41
Borgne R.	41	40	39	38	37	36	35	34	33	32	31	30	29	28	27	26	25	24	23	22	21	20	19	18	17	16	15	14	13	12	11	10	9	8	7	6	5	4	3	2	1	0	1	2	3	4	5	6	7	8	9	10	11	12	13	14	15	16	17	18	19	20	21	22	23	24	25	26	27	28	29	30	31	32	42	43	44	33	34	35	36	37	38	39	40
Sud R.	42	41	40	39	38	37	36	35	34	33	32	31	30	29	28	27	26	25	24	23	22	21	20	19	18	17	16	15	14	13	12	11	10	9	8	7	6	5	4	3	2	1	0	1	2	3	4	5	6	7	8	9	10	11	12	13	14	15	16	17	18	19	20	21	22	23	24	25	26	27	28	29	30	31	43	44	45	32	33	34	35	36	37	38	39
Pointe de l'épave Brook	43	42	41	40	39	38	37	36	35	34	33	32	31	30	29	28	27	26	25	24	23	22	21	20	19	18	17	16	15	14	13	12	11	10	9	8	7	6	5	4	3	2	1	0	1	2	3	4	5	6	7	8	9	10	11	12	13	14	15	16	17	18	19	20	21	22	23	24	25	26	27	28	29	30	44	45	46	31	32	33	34	35	36	37	38
Américains R.	44	43	42	41	40	39	38	37	36	35	34	33	32	31	30	29	28	27	26	25	24	23	22	21	20	19	18	17	16	15	14	13	12	11	10	9	8	7	6	5	4	3	2	1	0	1	2	3	4	5	6	7	8	9	10	11	12	13	14	15	16	17	18	19	20	21	22	23	24	25	26	27	28	29	45	46	47	30	31	32	33	34	35	36	37
3 Lacs R.	45	44	43	42	41	40	39	38	37	36	35	34	33	32	31	30	29	28	27	26	25	24	23	22	21	20	19	18	17	16	15	14	13	12	11	10	9	8	7	6	5	4	3	2	1	0	1	2	3	4	5	6	7	8	9	10	11	12	13	14	15	16	17	18	19	20	21	22	23	24	25	26	27	28	46	47	48	29	30	31	32	33	34	35	36
Val d'Auge R.	46	45	44	43	42	41	40	39	38	37	36	35	34	33	32	31	30	29	28	27	26	25	24	23	22	21	20	19	18	17	16	15	14	13	12	11	10	9	8	7	6	5	4	3	2	1	0	1	2	3	4	5	6	7	8	9	10	11	12	13	14	15	16	17	18	19	20	21	22	23	24	25	26	27	47	48	49	28	29	30	31	32	33	34	35
Anse de St Malo R.	47	46	45	44	43	42	41	40	39	38	37	36	35	34	33	32	31	30	29	28	27	26	25	24	23	22	21	20	19	18	17	16	15	14	13	12	11	10	9	8	7	6	5	4	3	2	1	0	1	2	3	4	5	6	7	8	9	10	11	12	13	14	15	16	17	18	19	20	21	22	23	24	25	26	48	49	50	27	28	29	30	31	32	33	34
Basin downstream of Korrigans Lake	48	47	46	45	44	43	42	41	40	39	38	37	36	35	34	33	32	31	30	29	28	27	26	25	24	23	22	21	20	19	18	17	16	15	14	13	12	11	10	9	8	7	6	5	4	3	2	1	0	1	2	3	4	5	6	7	8	9	10	11	12	13	14	15	16	17	18	19	20	21	22	23	24	25	49	50	51	26	27	28	29	30	31	32	33
Baie de l'Observatoire R.	49	48	47	46	45	44	43	42	41	40	39	38	37	36	35	34	33	32	31	30	29	28	27	26	25	24	23	22	21	20	19	18	17	16	15	14	13	12	11	10	9	8	7	6	5	4	3	2	1	0	1	2	3	4	5	6	7	8	9	10	11	12	13	14	15	16	17	18	19	20	21	22	23	24	50	51	52	25	26	27	28	29	30	31	32
SW Brook on Bras Jules Laboureur	50	49	48	47	46	45	44	43	42	41	40	39	38	37	36	35	34	33	32	31	30	29	28	27	26	25	24	23	22	21	20	19	18	17	16	15	14	13	12	11	10	9	8	7	6	5	4	3	2	1	0	1	2	3	4	5	6	7	8	9	10	11	12	13	14	15	16	17	18	19	20	21	22	23	51	52	53	24	25	26	27	28	29	30	31
Grisanche R.	51	50	49	48	47	46	45	44	43	42	41	40	39	38	37	36	35	34	33	32	31	30	29	28	27	26	25	24	23	22	21	20	19	18	17	16	15	14	13	12	11	10	9	8	7	6	5	4	3	2	1	0	1	2	3	4	5	6	7	8	9	10	11	12	13	14	15	16	17	18	19	20	21	22	52	53	54	23	24	25	26	27	28	29	30
Lac des Fougères Outlet	52	51	50	49	48	47	46	45	44	43	42	41	40	39	38	37	36	35	34	33	32	31	30	29	28	27	26	25	24	23	22	21	20	19	18	17	16	15	14	13	12	11	10	9	8	7	6	5	4	3	2	1	0	1	2	3	4	5	6	7	8	9	10	11	12	13	14	15	16	17	18	19	20	21	53	54	55	22	23	24	25	26	27	28	29
Lac d'Armor Basin	53	52	51	50	49	48	47	46	45	44	43	42	41	40	39	38	37	36	35	34	33	32	31	30	29	28	27	26	25	24	23	22	21	20	19	18	17	16	15	14	13	12	11	10	9	8	7	6	5	4	3	2	1	0	1	2	3	4	5	6	7	8	9	10	11	12	13	14	15	16	17	18	19	20	54	55	56	21	22	23	24	25	26	27	28
Anse J. Bourcart Brook	54	53	52	51	50	49	48	47	46	45	44	43	42	41	40	39	38	37	36	35	34	33	32	31	30	29	28	27	26	25	24	23	22	21	20	19	18	17	16	15	14	13	12	11	10	9	8	7	6	5	4	3	2	1	0	1	2	3	4	5	6	7	8	9	10	11	12	13	14	15	16	17	18	19	55	56	57	20	21	22	23	24	25	26	27
Ravin du Charbon R.	55	54	53	52	51	50	49	48	47	46	45	44	43	42	41	40	39	38	37	36	35	34	33	32	31	30	29	28	27	26	25	24	23	22	21	20	19	18	17	16	15	14	13	12	11	10	9	8	7	6	5	4	3	2	1	0	1	2	3	4	5	6	7	8	9	10	11	12	13	14	15	16	17	18	56	57	58	19	20	21	22	23	24	25	26
Vallée des Neiges Basin	56	55	54	53	52	51	50	49	48	47	46	45	44	43	42	41	40	39	38	37	36	35	34	33	32	31	30	29	28	27	26	25	24	23	22	21	20	19	18	17	16	15	14	13	12	11	10	9	8	7	6	5	4	3	2	1	0	1	2	3	4	5	6	7	8	9	10	11	12	13	14	15	16	17	57	58	59	18	19	20	21	22	23	24	25
Acaena R.	57	56	55	54	53	52	51	50	49	48	47	46	45	44	43	42	41	40	39	38	37	36	35	34	33	32	31	30	29	28	27	26	25	24	23	22	21	20	19	18	17	16	15	14	13	12	11	10	9	8	7	6	5	4	3	2	1	0	1	2	3	4	5	6	7	8	9	10	11	12	13	14	15	16	58	59	60	17	18	19	20	21	22	23	24
Vallée de la Planchette R.	58	57	56	55	54	53	52	51	50	49	48	47	46	45	44	43	42	41	40	39	38	37	36	35	34	33	32	31	30	29	28	27	26	25	24	23	22	21	20	19	18	17	16	15	14	13	12	11	10	9	8	7	6	5	4	3	2	1	0	1	2	3	4	5	6	7	8	9	10	11	12	13	14	15	59	60	61	16	17	18	19	20	21	22	23
Val Raide Brook	59	58	57	56	55	54	53	52	51	50	49	48	47	46	45	44	43	42	41	40	39	38	37	36	35	34	33	32	31	30	29	28	27	26	25	24	23	22	21	20	19	18	17	16	15	14	13	12	11	10	9	8	7	6	5	4	3	2	1	0	1	2	3	4	5	6	7	8	9	10	11	12	13	14	60	61	62	15	16	17	18	19	20	21	22
Mouettes R.	60	59	58	57	56	55	54	53	52	51	50	49	48	47	46	45	44	43	42	41	40	39	38	37	36	35	34	33	32	31	30	29	28	27	26	25	24	23	22	21	20	19	18	17	16	15	14	13	12	11	10	9	8	7	6	5	4	3	2	1	0	1	2	3	4	5	6	7	8	9	10	11	12	13	61	62	63	14	15	16	17	18	19	20	21
Macaronis R.	61	60	59	58	57	56	55	54	53	52	51	50	49	48	47	46	45	44	43	42	41	40	39	38	37	36	35	34	33	32	31	30	29	28	27	26	25	24	23	22	21	20	19	18	17	16	15	14	13	12	11	10	9	8	7	6	5	4	3	2	1	0	1	2	3	4	5	6	7	8	9	10	11	12	62	63	64	13	14	15	16	17	18	19	20
Vallée Phonolite Basin	62	61	60	59	58	57	56	55	54	53	52	51	50	49	48	47	46	45	44	43	42	41	40	39	38	37	36	35	34	33	32	31	30	29	28	27	26	25	24	23	22	21	20	19	18	17	16	15	14	13	12	11	10	9	8	7	6	5	4	3	2	1	0	1	2	3	4	5	6	7	8	9	10	11	63	64	65	12	13	14	15	16	17	18	19
Val du Levant R.	63	62	61	60	59	58	57	56	55	54	53	52	51	50	49	48	47	46	45	44	43	42	41	40	39	38	37	36	35	34	33	32	31	30	29	28	27	26	25	24	23	22	21	20	19	18	17	16	15	14	13	12	11	10	9	8	7	6	5	4	3	2	1	0	1	2	3	4	5	6	7	8	9	10	64	65	66	11	12	13	14	15	16	17	18
Anse de la Canicule R.	64	63	62	61	60	59	58	57	56	55	54	53	52	51	50	49	48	47	46	45	44	43	42	41	40	39	38	37	36	35	34	33	32	31	30	29	28	27	26	25	24	23	22	21	20	19	18	17	16	15	14	13	12	11	10	9	8	7	6	5	4	3	2	1	0	1	2	3	4	5	6	7	8	9	65	66	67	10	11	12	13	14	15	16	17
Crête des Laves R.	65	64	63	62	61	60	59	58	57	56	55	54	53	52	51	50	49	48	47	46	45	44	43	42	41	40	39	38	37	36	35	34	33	32	31	30	29	28	27	26	25	24	23	22	21	20	19	18	17	16	15	14	13	12	11	10	9	8	7	6	5	4	3	2	1	0	1	2	3	4	5	6	7	8	66	67	68	9	10	11	12	13	14	15	16
Halage des Swains R.	66	65	64	63	62	61	60	59	58	57	56	55	54	53	52	51	50	49	48	47	46	45	44	43	42	41	40	39	38	37	36	35	34	33	32	31	30	29	28	27	26	25	24	23	22	21	20	19	18	17	16	15	14	13	12	11	10	9	8	7	6	5	4	3	2	1	0	1	2	3	4	5	6	7	67	68	69	8	9	10	11	12	13	14	15
Northern arm of Anse aux Ecueils Brook	67	66	65	64	63	62	61	60	59	58	57	56	55	54	53	52	51	50	49	48	47	46	45	44	43	42	41	40	39	38	37	36	35	34	33	32	31	30	29	28	27	26	25	24	23	22	21	20	19	18	17	16	15	14	13	12	11	10	9	8	7	6	5	4	3	2	1	0	1	2	3	4	5	6	68	69	70	7	8	9	10	11	12	13	14
La Clarée Basin: Hermance Lake & Galets R.	68	67	66	65	64	63	62	61	60	59	58	57	56	55	54	53	52	51	50	49	48	47	46	45	44	43	42	41	40	39	38	37	36	35	34	33	32	31	30	29	28	27	26	25	24	23	22	21	20	19	18	17	16	15	14	13	12	11	10	9	8	7	6	5	4	3	2	1	0	1	2	3	4	5	69	70	71	6	7	8	9	10	11	12	13
Olsen R.	69	68	67	66	65	64	63	62	61	60	59	58	57	56	55	54	53	52	51	50	49	48	47	46	45	44	43	42	41	40	39	38	37	36	35	34	33	32	31	30	29	28	27	26	25	24	23	22	21	20	19	18	17	16	15	14	13	12	11	10	9	8	7	6	5	4	3	2	1	0	1	2	3	4	70	71	72	5	6	7	8	9	10	11	12
Vallée du Radioleine R.	70	69	68	67	66	65	64	63	62	61	60	59	58	57	56	55	54	53	52	51	50	49	48	47	46	45	44	43	42	41	40	39	38	37	36	35	34	33	32	31	30	29	28	27	26	25	24	23	22	21	20	19	18	17	16	15	14	13	12	11	10	9	8	7	6	5	4	3	2	1	0	1	2	3	71	72	73	4	5	6	7	8	9	10	11
Val des Skuas R.	71	70	69	68	67	66	65	64	63	62	61	60	59	58	57	56	55	54	53	52	51	50	49	48	47	46	45	44	43	42	41	40	39	38	37	36	35	34	33	32	31	30	29	28	27	26	25	24	23	22	21	20	19	18	17	16	15	14	13	12	11	10	9	8	7	6	5	4	3	2	1	0	1	2	72	73	74	3	4	5	6	7	8	9	10
Doigt de Sainte-Anne R.	72	71	70	69	68	67	66	65	64	63	62	61	60	59	58	57	56	55	54	53	52	51	50	49	48	47	46	45	44	43	42	41	40	39	38	37	36	35	34	33	32	31	30	29	28	27	26	25	24	23	22	21	20	19	18	17	16	15	14	13	12	11	10	9	8	7	6	5	4	3	2	1	0	1	73	74	75	2	3	4	5	6	7	8	9
Plaine de Dante R.	73	72	71	70	69	68	67	66	65	64	63	62	61	60	59	58	57	56	55	54	53	52	51	50	49	48	47	46	45	44	43	42	41	40	39	38	37	36	35	34	33	32	31	30	29	28	27	26	25	24	23	22	21	20	19	18	17	16	15	14	13	12	11	10	9	8	7	6	5	4	3	2	1	0	74	75	76	1	2	3	4	5	6	7	8
Mont Ventoux R.	1	2	3	4	5	6	7	8	9	10	11	12	13	14	15	16	17	18	19	20	21	22	23	24	25	26	27	28	29	30	31	32	33	34	35	36	37	38	39	40	41	42	43	44	45	46	47	48	49	50	51	52	53	54	55	56	57	58	59	60	61	62	63	64	65	66	67	68	69	70	71	72	73	74	0	1	2	75	76	77	78	79	80	81	82
Mont Sinaï R.	2	3	4	5	6	7	8	9	10	11	12	13	14	15	16	17	18	19	20	21	22	23	24	25	26	27	28	29	30	31	32	33	34	35	36	37	38	39	40	41	42	43	44	45	46	47	48	49	50	51	52	53	54	55	56	57	58	59	60	61	62	63	64	65	66	67	68	69	70	71	72	73	74	75	1	0	1	76	77	78	79	80	81	82	83
Bastille R.	3	4	5	6	7	8	9	10	11	12	13	14	15	16	17	18	19	20	21	22	23	24	25	26	27	28	29	30	31	32	33	34	35	36	37	38	39	40	41	42	43	44	45	46	47	48	49	50	51	52	53	54	55	56	57	58	59	60	61	62	63	64	65	66	67	68	69	70	71	72	73	74	75	76	2	1	0	77	78	79	80	81	82	83	84
Lac Mariot outlet	74	73	72	71	70	69	68	67	66	65	64	63	62	61	60	59	58	57	56	55	54	53	52	51	50	49	48	47	46	45	44	43	42	41	40	39	38	37	36	35	34	33	32	31	30	29	28	27	26	25	24	23	22	21	20	19	18	17	16	15	14	13	12	11	10	9	8	7	6	5	4	3	2	1	75	76	77	0	1	2	3	4	5	6	7
Plaine d'Ampère R.	75	74	73	72	71	70	69	68	67	66	65	64	63	62	61	60	59	58	57	56	55	54	53	52	51	50	49	48	47	46	45	44	43	42	41	40	39	38	37	36	35	34	33	32	31	30	29	28	27	26	25	24	23	22	21	20	19	18	17	16	15	14	13	12	11	10	9	8	7	6	5	4	3	2	76	77	78	1	0	1	2	3	4	5	6
Portes Noires R.	76	75	74	73	72	71	70	69	68	67	66	65	64	63	62	61	60	59	58	57	56	55	54	53	52	51	50	49	48	47	46	45	44	43	42	41	40	39	38	37	36	35	34	33	32	31	30	29	28	27	26	25	24	23	22	21	20	19	18	17	16	15	14	13	12	11	10	9	8	7	6	5	4	3	77	78	79	2	1	0	1	2	3	4	5
Contacts R.	77	76	75	74	73	72	71	70	69	68	67	66	65	64	63	62	61	60	59	58	57	56	55	54	53	52	51	50	49	48	47	46	45	44	43	42	41	40	39	38	37	36	35	34	33	32	31	30	29	28	27	26	25	24	23	22	21	20	19	18	17	16	15	14	13	12	11	10	9	8	7	6	5	4	78	79	80	3	2	1	0	1	2	3	4
Mouche R.	78	77	76	75	74	73	72	71	70	69	68	67	66	65	64	63	62	61	60	59	58	57	56	55	54	53	52	51	50	49	48	47	46	45	44	43	42	41	40	39	38	37	36	35	34	33	32	31	30	29	28	27	26	25	24	23	22	21	20	19	18	17	16	15	14	13	12	11	10	9	8	7	6	5	79	80	81	4	3	2	1	0	1	2	3
Larmor R.	79	78	77	76	75	74	73	72	71	70	69	68	67	66	65	64	63	62	61	60	59	58	57	56	55	54	53	52	51	50	49	48	47	46	45	44	43	42	41	40	39	38	37	36	35	34	33	32	31	30	29	28	27	26	25	24	23	22	21	20	19	18	17	16	15	14	13	12	11	10	9	8	7	6	80	81	82	5	4	3	2	1	0	1	2
Plage Jaune R.	80	79	78	77	76	75	74	73	72	71	70	69	68	67	66	65	64	63	62	61	60	59	58	57	56	55	54	53	52	51	50	49	48	47	46	45	44	43	42	41	40	39	38	37	36	35	34	33	32	31	30	29	28	27	26	25	24	23	22	21	20	19	18	17	16	15	14	13	12	11	10	9	8	7	81	82	83	6	5	4	3	2	1	0	1
Sables R.	81	80	79	78	77	76	75	74	73	72	71	70	69	68	67	66	65	64	63	62	61	60	59	58	57	56	55	54	53	52	51	50	49	48	47	46	45	44	43	42	41	40	39	38	37	36	35	34	33	32	31	30	29	28	27	26	25	24	23	22	21	20	19	18	17	16	15	14	13	12	11	10	9	8	82	83	84	7	6	5	4	3	2	1	0

Labonne et al. 2013, Appendix 2A: Distance matrix between hydrographic systems with a stepping-stone approach

Patch names	Entrelacs R	Baie Chanzy	Déversoir du Lac de la Croix du Sud	Outlet of Lac Euphrosine	Ballon R.	Sans Nom R.	S. Brook Ile aux Skuas	Brook 1 Ile du Port	Brook 2 Ile du Port	Brook 3 Ile du Port	Brook 4 Ile du Port	Val Froide R.	Val Travers R	Brook N. of Port-Couvreux	Brook of Port-Couvreux cabin	Brook W end of Havre du Beau Temps	Anse Sablonneuse Brook	Port Kirk R.	Val de l'Ouest R.	Studer R.	Doute R.	Charbon R.	Sérail R.	Chasseurs R.	Nord R.	Pépins R.	Cataractes R.	Hautes Mares South Brook (Gorfous 1)
Entrelacs R.	0	24,83	25,03	32,37	31,75	31,17	30,95	33,25	34,12	35,53	36,24	39,23	34,08	46,91	47,82	58,3	49,27	56,18	56,4	62,75	68,13	68,36	72,62	77,42	90,83	92,25	92,47	93,37
Baie Chanzy	24,83	0	10,44	20,31	19,46	19,01	19	21,53	23,28	24,77	25,53	27,42	22,23	35,141	36,546	47,05	37,97	45,005	45,225	51,575	56,955	57,185	61,445	66,245	79,655	81,075	81,295	82,195
Déversoir du Lac de la Croix du Sud	25,03	10,44	0	15,923	15,073	14,623	14,613	17,297	19,047	20,537	21,297	23,033	17,843	30,754	32,159	42,663	33,583	40,618	40,838	47,188	52,568	52,798	57,058	61,858	75,268	76,688	76,908	77,808
Outlet of Lac Euphrosine	32,37	20,31	15,923	0	1,59	3,04	13,12	15,804	17,554	19,044	19,804	20,279	15,089	28	29,405	39,909	30,829	37,864	38,084	44,434	49,814	50,044	54,304	59,104	72,514	73,934	74,154	75,054
Ballon R.	31,75	19,46	15,073	1,59	0	1,78	12,389	15,073	16,823	18,313	19,073	19,404	14,214	27,125	28,53	39,034	29,954	36,989	37,209	43,559	48,939	49,169	53,429	58,229	71,639	73,059	73,279	74,179
Sans Nom R.	31,17	19,01	14,623	3,04	1,78	0	11,982	14,666	16,416	17,906	18,666	18,96	13,77	26,681	28,086	38,59	29,51	36,545	36,765	43,115	48,495	48,725	52,985	57,785	71,195	72,615	72,835	73,735
S. Brook Ile aux Skuas	30,95	19	14,613	13,12	12,389	11,982	0	12,51	14,25	15,87	16,65	13,23	7,77	16,794	18,199	28,703	19,623	26,658	26,878	33,228	38,608	38,838	43,098	47,898	61,308	62,728	62,948	63,848
Brook 1 Ile du Port	33,25	21,53	17,297	15,804	15,073	14,666	12,51	0	3,52	4,98	5,81	20,926	15,736	28,647	30,052	40,556	31,476	38,511	38,731	45,081	50,461	50,691	54,951	59,751	73,161	74,581	74,801	75,701
Brook 2 Ile du Port	34,12	23,28	19,047	17,554	16,823	16,416	14,25	3,52	0	3,67	4,48	22,5	17,31	30,221	31,626	42,13	33,05	40,085	40,305	46,655	52,035	52,265	56,525	61,325	74,735	76,155	76,375	77,275
Brook 3 Ile du Port	35,53	24,77	20,537	19,044	18,313	17,906	15,87	4,98	3,67	0	1,89	24,119	18,929	31,84	33,245	43,749	34,669	41,704	41,924	48,274	53,654	53,884	58,144	62,944	76,354	77,774	77,994	78,894
Brook 4 Ile du Port	36,24	25,53	21,297	19,804	19,073	18,666	16,65	5,81	4,48	1,89	0	24,908	19,718	32,629	34,034	44,538	35,458	42,493	42,713	49,063	54,443	54,673	58,933	63,733	77,143	78,563	78,783	79,683
Val Froide R.	39,23	27,42	23,033	20,279	19,404	18,96	13,23	20,926	22,5	24,119	24,908	0	6,37	29,172	30,577	41,081	32,001	39,036	39,256	45,606	50,986	51,216	55,476	60,276	73,686	75,106	75,326	76,226
Val Travers R.	34,08	22,23	17,843	15,089	14,214	13,77	7,77	15,736	17,31	18,929	19,718	6,37	0	23,629	25,034	35,538	26,458	33,493	33,713	40,063	45,443	45,673	49,933	54,733	68,143	69,563	69,783	70,683
Brook N. of Port-Couvreux	46,91	35,141	30,754	28	27,125	26,681	16,794	28,647	30,221	31,84	32,629	29,172	23,629	0	3,44	14,27	5,26	12,99	13	19,389	24,769	24,999	29,259	34,059	47,469	48,889	49,109	50,009
Brook of Port-Couvreux cabin	47,82	36,546	32,159	29,405	28,53	28,086	18,199	30,052	31,626	33,245	34,034	30,577	25,034	3,44	0	12,79	8,82	12,87	12,73	19,222	24,602	24,832	29,092	33,892	47,302	48,722	48,942	49,842
Brook W end of Havre du Beau Temps	58,3	47,05	42,663	39,909	39,034	38,59	28,703	40,556	42,13	43,749	44,538	41,081	35,538	14,27	12,79	0	12,07	22,55	23,03	29,193	34,573	34,803	39,063	43,863	57,273	58,693	58,913	59,813
Anse Sablonneuse Brook	49,27	37,97	33,583	30,829	29,954	29,51	19,623	31,476	33,05	34,669	35,458	32,001	26,458	5,26	8,82	12,07	0	13,45	14	20,358	25,738	25,968	30,228	35,028	48,438	49,858	50,078	50,978
Port Kirk R.	56,18	45,005	40,618	37,864	36,989	36,545	26,658	38,511	40,085	41,704	42,493	39,036	33,493	12,99	12,87	22,55	13,45	0	6,96	13,424	18,804	19,034	23,294	28,094	41,504	42,924	43,144	44,044
Val de l'Ouest R.	56,4	45,225	40,838	38,084	37,209	36,765	26,878	38,731	40,305	41,924	42,713	39,256	33,713	13	12,73	23,03	14	6,96	0	9,009	14,389	14,619	18,879	23,679	37,089	38,509	38,729	39,629
Studer R.	62,75	51,575	47,188	44,434	43,559	43,115	33,228	45,081	46,655	48,274	49,063	45,606	40,063	19,389	19,222	29,193	20,358	13,424	9,009	0	7,415	7,645	11,905	16,705	30,115	31,535	31,755	32,655
Doute R.	68,13	56,955	52,568	49,814	48,939	48,495	38,608	50,461	52,035	53,654	54,443	50,986	45,443	24,769	24,602	34,573	25,738	18,804	14,389	7,415	0	2,761	7,021	11,821	25,231	26,651	26,871	27,771
Charbon R.	68,36	57,185	52,798	50,044	49,169	48,725	38,838	50,691	52,265	53,884	54,673	51,216	45,673	24,999	24,832	34,803	25,968	19,034	14,619	7,645	2,761	0	7,374	12,174	25,584	27,004	27,224	28,124
Sérail R.	72,62	61,445	57,058	54,304	53,429	52,985	43,098	54,951	56,525	58,144	58,933	55,476	49,933	29,259	29,092	39,063	30,228	23,294	18,879	11,905	7,021	7,374	0	6,418	19,828	21,248	21,468	22,368
Chasseurs R.	77,42	66,245	61,858	59,104	58,229	57,785	47,898	59,751	61,325	62,944	63,733	60,276	54,733	34,059	33,892	43,863	35,028	28,094	23,679	16,705	11,821	12,174	6,418	0	15,418	16,838	17,058	17,958
Nord R.	90,83	79,655	75,268	72,514	71,639	71,195	61,308	73,161	74,735	76,354	77,143	73,686	68,143	47,469	47,302	57,273	48,438	41,504	37,089	30,115	25,231	25,584	19,828	15,418	0	7,82	8,01	9,04
Pépins R.	92,25	81,075	76,688	73,934	73,059	72,615	62,728	74,581	76,155	77,774	78,563	75,106	69,563	48,889	48,722	58,693	49,858	42,924	38,509	31,535	26,651	27,004	21,248	16,838	7,82	0	3,23	4,37
Cataractes R.	92,47	81,295	76,908	74,154	73,279	72,835	62,948	74,801	76,375	77,994	78,783	75,326	69,783	49,109	48,942	58,913	50,078	43,144	38,729	31,755	26,871	27,224	21,468	17,058	8,01	3,23	0	1,89
Hautes Mares South Brook (Gorfous 1)	93,37	82,195	77,808	75,054	74,179	73,735	63,848	75,701	77,275	78,894	79,683	76,226	70,683	50,009	49,842	59,813	50,978	44,044	39,629	32,655	27,771	28,124	22,368	17,958	9,04	4,37	1,89	0
Hautes Mares North Brook (Gorfous 2)	93,52	82,345	77,958	75,204	74,329	73,885	63,998	75,851	77,425	79,044	79,833	76,376	70,833	50,159	49,992	59,963	51,128	44,194	39,779	32,805	27,921	28,274	22,518	18,108	9,18	4,73	2,05	0,44
Pointe des Cabanes Brook (Gorfous 4)	96,31	85,135	80,748	77,994	77,119	76,675	66,788	78,641	80,215	81,834	82,623	79,166	73,623	52,949	52,782	62,753	53,918	46,984	42,569	35,595	30,711	31,064	25,308	20,898	12,01	7,36	4,87	3,8
Cap Rouge Brook (Gorfous 5)	101,09	89,915	85,528	82,774	81,899	81,455	71,568	83,421	84,995	86,614	87,403	83,946	78,403	57,729	57,562	67,533	58,698	51,764	47,349	40,375	35,491	35,844	30,088	25,678	16,82	12,44	9,95	8,41
N. Cap Rouge Brook (Gorfous 6)	101,47	90,295	85,908	83,154	82,279	81,835	71,948	83,801	85,375	86,994	87,783	84,326	78,783	58,109	57,942	67,913	59,078	52,144	47,729	40,755	35,871	36,224	30,468	26,058	17,34	12,89	10,28	8,88
Cap de Rohan R.	109,63	98,455	94,068	91,314	90,439	89,995	80,108	91,961	93,535	95,154	95,943	92,486	86,943	66,269	66,102	76,073	67,238	60,304	55,889	48,915	44,031	44,384	38,628	34,218	25,543	20,977	18,578	17,351
Lac Marville Outlet	127,24	116,07	111,68	108,92	108,05	107,61	97,718	109,57	111,15	112,76	113,55	110,1	104,55	83,879	83,712	93,683	84,848	77,914	73,499	66,525	61,641	61,994	56,238	51,828	43,153	38,587	36,188	34,961
Manchots R.	136,78	125,61	121,22	118,46	117,59	117,15	107,26	119,11	120,69	122,3	123,09	119,64	114,09	93,419	93,252	103,22	94,388	87,454	83,039	76,065	71,181	71,534	65,778	61,368	52,693	48,127	45,728	44,501
Calcédoines R.	149,29	138,12	133,73	130,97	130,1	129,66	119,77	131,62	133,2	134,81	135,6	132,15	126,6	105,93	105,76	115,73	106,9	99,964	95,549	88,575	83,691	84,044	78,288	73,878	65,203	60,637	58,238	57,011
Bungay R.	153,6	142,43	138,04	135,28	134,41	133,97	124,08	135,93	137,51	139,12	139,91	136,46	130,91	110,24	110,07	120,04	111,21	104,27	99,859	92,885	88,001	88,354	82,598	78,188	69,513	64,947	62,548	61,321
Albatros R.	162,91	151,74	147,35	144,59	143,72	143,28	133,39	145,24	146,82	148,43	149,22	145,77	140,22	119,55	119,38	129,35	120,52	113,58	109,17	102,2	97,311	97,664	91,908	87,498	78,823	74,257	71,858	70,631
Norvégienne R.	164,79	153,62	149,23	146,47	145,6	145,16	135,27	147,12	148,7	150,31	151,1	147,65	142,1	121,43	121,26	131,23	122,4	115,46	111,05	104,08	99,191	99,544	93,788	89,378	80,703	76,137	73,738	72,511
Château R.	166,04	154,87	150,48	147,72	146,85	146,41	136,52	148,37	149,95	151,56	152,35	148,9	143,35	122,68	122,51	132,48	123,65	116,71	112,3	105,33	100,44	100,79	95,038	90,628	81,953	77,387	74,988	73,761
PAF Ferme R.	202,07	190,9	186,51	183,75	182,88	182,44	172,55	184,4	185,98	187,59	188,38	184,93	179,38	158,71	158,54	168,51	159,68	152,74	148,33	141,36	136,47	136,82	131,07	126,66	117,98	113,42	111,02	109,79
Borgne R.	204,73	193,56	189,17	186,41	185,54	185,1	175,21	187,06	188,64	190,25	191,04	187,59	182,04	161,37	161,2	171,17	162,34	155,4	150,99	144,02	139,13	139,48	133,73	129,32	120,64	116,08	113,68	112,45
Sud R.	207,32	196,15	191,76	189	188,13	187,69	177,8	189,65	191,23	192,84	193,63	190,18	184,63	163,96	163,79	173,76	164,93	157,99	153,58	146,61	141,72	142,07	136,32	131,91	123,23	118,67	116,27	115,04
Pointe de l'épave Brook	208,77	197,6	193,21	190,45	189,58	189,14	179,25	191,1	192,68	194,29	195,08	191,63	186,08	165,41	165,24	175,21	166,38	159,44	155,03	148,06	143,17	143,52	137,77	133,36	124,68	120,12	117,72	116,49
Américains R.	211,05	199,88	195,49	192,73	191,86	191,42	181,53	193,38	194,96	196,57	197,36	193,91	188,36	167,69	167,52	177,49	168,66	161,72	157,31	150,34	145,45	145,8	140,05	135,64	126,96	122,4	120	118,77
3 Lacs R.	217,42	206,25	201,86	199,1	198,23	197,79	187,9	199,75	201,33	202,94	203,73	200,28	194,73	174,06	173,89	183,86	175,03	168,09	163,68	156,71	151,82	152,17	146,42	142,01	133,33	128,77	126,37	125,14
Val d'Auge R.	225,19	214,02	209,63	206,87	206	205,56	195,67	207,52	209,1	210,71	211,5	208,05	202,5	181,83	181,66	191,63	182,8	175,86	171,45	164,48	159,59	159,94	154,19	149,78	141,1	136,54	134,14	132,91
Anse de St Malo R.	232,8	221,63	217,24	214,48	213,61	213,17	203,28	215,13	216,71	218,32	219,11	215,66	210,11	189,44	189,27	199,24	190,41	183,47	179,06	172,09	167,2	167,55	161,8	157,39	148,71	144,15	141,75	140,52
Basin downstream of Korrigans Lake	228,25	217,08	212,69	209,93	209,06	208,62	198,73	210,58	212,16	213,77	214,56	211,11	205,56	184,89	184,72	194,69	185,86	178,92	174,51	167,54	162,65	163	157,25	152,84	144,16	139,6	137,2	135,97
Baie de l'Observatoire R.	227,67	216,5	212,11	209,35	208,48	208,04	198,15	210	211,58	213,19	213,98	210,53	204,98	184,31	184,14	194,11	185,28	178,34	173,93	166,96	162,07	162,42	156,67	152,26	143,58	139,02	136,62	135,39
SW Brook on Bras Jules Laboureur	237,9	226,73	222,34	219,58	218,71	218,27	208,38	220,23	221,81	223,42	224,21	220,76	215,21	194,54	194,37	204,34	195,51	188,57	184,16	177,19	172,3	172,65	166,9	162,49	153,81	149,25	146,85	145,62
Grisanche R.	257,47	246,3	241,91	239,15	238,28	237,84	227,95	239,8	241,38	242,99	243,78	240,33	234,78	214,11	213,94	223,91	215,08	208,14	203,73	196,76	191,87	192,22	186,47	182,06	173,38	168,82	166,42	165,19
Lac des Fougères Outlet	253,43	242,26	237,87	235,11	234,24	233,8	223,91	235,76	237,34	238,95	239,74	236,29	230,74	210,07	209,9	219,87	211,04	204,1	199,69	192,72	187,83	188,18	182,43	178,02	169,34	164,78	162,38	161,15
Lac d'Armor Basin	245,57	234,4	230,01	227,25	226,38	225,94	216,05	227,9	229,48	231,09	231,88	228,43	222,88	202,21	202,04	212,01	203,18	196,24	191,83	184,86	179,97	180,32	174,57	170,16	161,48	156,92	154,52	153,29
Anse J. Bourcart Brook	241,87	230,7	226,31	223,55	222,68	222,24	212,35	224,2	225,78	227,39	228,18	224,73	219,18	198,51	198,34	208,31	199,48	192,54	188,13	181,16	176,27	176,62	170,87	166,46	157,78	153,22	150,82	149,59
Ravin du Charbon R.	244,9	233,73	229,34	226,58	225,71	225,27	215,38	227,23	228,81	230,42	231,21	227,76	222,21	201,54	201,37	211,34	202,51	195,57	191,16	184,19	179,3	179,65	173,9	169,49	160,81	156,25	153,85	152,62
Vallée des Neiges Basin	248,81	237,64	233,25	230,49	229,62	229,18	219,29	231,14	232,72	234,33	235,12	231,67	226,12	205,45	205,28	215,25	206,42	199,48	195,07	188,1	183,21	183,56	177,81	173,4	164,72	160,16	157,76	156,53
Acaena R.	247,98	236,81	232,42	229,66	228,79	228,35	218,46	230,31	231,89	233,5	234,29	230,84	225,29	204,62	204,45	214,42	205,59	198,65	194,24	187,27	182,38	182,73	176,98	172,57	163,89	159,33	156,93	155,7
Vallée de la Planchette R.	245,24	234,07	229,68	226,92	226,05	225,61	215,72	227,57	229,15	230,76	231,55	228,1	222,55	201,88	201,71	211,68	202,85	195,91	191,5	184,53	179,64	179,99	174,24	169,83	161,15	156,59	154,19	152,96
Val Raide Brook	253,24	242,07	237,68	234,92	234,05	233,61	223,72	235,57	237,15	238,76	239,55	236,1	230,55	209,88	209,71	219,68	210,85	203,91	199,5	192,53	187,64	187,99	182,24	177,83	169,15	164,59	162,19	160,96
Mouettes R.	246,2	235,03	230,64	227,88	227,01	226,57	216,68	228,53	230,11	231,72	232,51	229,06	223,51	202,84	202,67	212,64	203,81	196,87	192,46	185,49	180,6	180,95	175,2	170,79	162,11	157,55	155,15	153,92
Macaronis R.	258,65	247,48	243,09	240,33	239,46	239,02	229,13	240,98	242,56	244,17	244,96	241,51	235,96	215,29	215,12	225,09	216,26	209,32	204,91	197,94	193,05	193,4	187,65	183,24	174,56	170	167,6	166,37
Vallée Phonolite Basin	270,7	259,53	255,14	252,38	251,51	251,07	241,18	253,03	254,61	256,22	257,01	253,56	248,01	227,34	227,17	237,14	228,31	221,37	216,96	209,99	205,1	205,45	199,7	195,29	186,61	182,05	179,65	178,42
Val du Levant R.	283,96	272,79	268,4	265,64	264,77	264,33	254,44	266,29	267,87	269,48	270,27	266,82	261,27	240,6	240,43	250,4	241,57	234,63	230,22	223,25	218,36	218,71	212,96	208,55	199,87	195,31	192,91	191,68
Anse de la Canicule R.	311,6	300,43	296,04	293,28	292,41	291,97	282,08	293,93	295,51	297,12	297,91	294,46	288,91	268,24	268,07	278,04	269,21	262,27	257,86	250,89	246	246,35	240,6	236,19	227,51	222,95	220,55	219,32
Crête des Laves R.	313,72	302,55	298,16	295,4	294,53	294,09	284,2	296,05	297,63	299,24	300,03	296,58	291,03	270,36	270,19	280,16	271,33	264,39	259,98	253,01	248,12	248,47	242,72	238,31	229,63	225,07	222,67	221,44
Halage des Swains R.	324,71	313,54	309,15	306,39	305,52	305,08	295,19	307,04	308,62	310,23	311,02	307,57	302,02	281,35	281,18	291,15	282,32	275,38	270,97	264	259,11	259,46	253,71	249,3	240,62	236,06	233,66	232,43
Northern arm of Anse aux Ecueils Brook	333,82	322,65	318,26	315,5	314,63	314,19	304,3	316,15	317,73	319,34	320,13	316,68	311,13	290,46	290,29	300,26	291,43	284,49	280,08	273,11	268,22	268,57	262,82	258,41	249,73	245,17	242,77	241,54
La Clarée Basin: Hermance Lake & Galets R.	334,1	322,93	318,54	315,78	314,91	314,47	304,58	316,43	318,01	319,62	320,41	316,96	311,41	290,74	290,57	300,54	291,71	284,77	280,36	273,39	268,5	268,85	263,1	258,69	250,01	245,45	243,05	241,82
Olsen R.	332,39	321,22	316,83	314,07	313,2	312,76	302,87	314,72	316,3	317,91	318,7	315,25	309,7	289,03	288,86	298,83	290	283,06	278,65	271,68	266,79	267,14	261,39	256,98	248,3	243,74	241,34	240,11
Vallée du Radioleine R.	328,43	317,26	312,87	310,11	309,24	308,8	298,91	310,76	312,34	313,95	314,74	311,29	305,74	285,07	284,9	294,87	286,04	279,1	274,69	267,72	262,83	263,18	257,43	253,02	244,34	239,78	237,38	236,15
Val des Skuas R.	336,69	325,52	321,13	318,37	317,5	317,06	307,17	319,02	320,6	322,21	323	319,55	314	293,33	293,16	303,13	294,3	287,36	282,95	275,98	271,09	271,44	265,69	261,28	252,6	248,04	245,64	244,41
Doigt de Sainte-Anne R.	355,97	344,79	340,41	337,65	336,78	336,33	326,45	338,3	339,87	341,49	342,28	338,82	333,28	312,61	312,44	322,41	313,58	306,64	302,23	295,25	290,37	290,72	284,97	280,56	271,88	267,31	264,92	263,69
Plaine de Dante R.	358,23	347,05	342,66	339,91	339,04	338,59	328,7	340,56	342,13	343,75	344,54	341,08	335,54	314,87	314,7	324,67	315,83	308,9	304,49	297,51	292,63	292,98	287,22	282,81	274,14	269,57	267,17	265,95
Mont Ventoux R.	2,67	22,716	22,916	30,256	29,636	29,056	28,836	31,136	32,006	33,416	34,126	37,116	31,966	44,796	45,706	56,186	47,156	54,066	54,286	60,636	66,016	66,246	70,506	75,306	88,716	90,136	90,356	91,256
Mont Sinaï R.	8,93	18,586	18,786	26,126	25,506	24,926	24,706	27,006	27,876	29,286	29,996	32,986	27,836	40,666	41,576	52,056	43,026	49,936	50,156	56,506	61,886	62,116	66,376	71,176	84,586	86,006	86,226	87,126
Bastille R.	45,54	41,401	41,601	48,941	48,321	47,741	47,521	49,821	50,691	52,101	52,811	55,801	50,651	63,481	64,391	74,871	65,841	72,751	72,971	79,321	84,701	84,931	89,191	93,991	107,4	108,82	109,04	109,94
Lac Mariot outlet	372,56	361,39	357	354,24	353,37	352,93	343,04	354,89	356,47	358,08	358,87	355,42	349,87	329,2	329,03	339	330,17	323,23	318,82	311,85	306,96	307,31	301,56	297,15	288,47	283,91	281,51	280,28
Plaine d'Ampère R.	375,22	364,05	359,66	356,9	356,03	355,59	345,7	357,55	359,13	360,74	361,53	358,08	352,53	331,86	331,69	341,66	332,83	325,89	321,48	314,51	309,62	309,97	304,22	299,81	291,13	286,57	284,17	282,94
Portes Noires R.	383,89	372,72	368,33	365,57	364,7	364,26	354,37	366,22	367,8	369,41	370,2	366,75	361,2	340,53	340,36	350,33	341,5	334,56	330,15	323,18	318,29	318,64	312,89	308,48	299,8	295,24	292,84	291,61
Contacts R.	408,3	397,13	392,74	389,98	389,11	388,67	378,78	390,63	392,21	393,82	394,61	391,16	385,61	364,94	364,77	374,74	365,91	358,97	354,56	347,59	342,7	343,05	337,3	332,89	324,21	319,65	317,25	316,02
Mouche R.	403,86	392,69	388,3	385,54	384,67	384,23	374,34	386,19	387,77	389,38	390,17	386,72	381,17	360,5	360,33	370,3	361,47	354,53	350,12	343,15	338,26	338,61	332,86	328,45	319,77	315,21	312,81	311,58
Larmor R.	398,15	386,98	382,59	379,83	378,96	378,52	368,63	380,48	382,06	383,67	384,46	381,01	375,46	354,79	354,62	364,59	355,76	348,82	344,41	337,44	332,55	332,9	327,15	322,74	314,06	309,5	307,1	305,87
Plage Jaune R.	407,08	395,91	391,52	388,76	387,89	387,45	377,56	389,41	390,99	392,6	393,39	389,94	384,39	363,72	363,55	373,52	364,69	357,75	353,34	346,37	341,48	341,83	336,08	331,67	322,99	318,43	316,03	314,8
Sables R.	413,67	402,5	398,11	395,35	394,48	394,04	384,15	396	397,58	399,19	399,98	396,53	390,98	370,31	370,14	380,11	371,28	364,34	359,93	352,96	348,07	348,42	342,67	338,26	329,58	325,02	322,62	321,39

Patch names	Hautes Mares North Brook (Gorfous 2)	Pointe des Cabanes Brook (Gorfous 4)	Cap Rouge Brook (Gorfous 5)	N. Cap Rouge Brook (Gorfous 6)	Cap de Rohan R.	Lac Marville Outlet	Manchots R	Calcédoines R	Bungay R.	Albatros R	Norvégienne R.	Château R.	PAF Ferme R.	Borgne R.	Sud R.	Pointe de l'épave Brook	Américains R.	3 Lacs R	Val d'Auge R.	Anse de St Malo R	Basin downstream of Korrigans Lake	Baie de l'Observatoire R.	SW Brook on Bras Jules Laboureur	Grisanche R.	Lac des Fougères Outlet	Lac d'Armor Basin	Anse J. Bourcart Brook	Ravin du Charbon R.	Vallée des Neiges Basin	Acaena R.
Entrelacs R.	93,52	96,31	101,09	101,47	109,63	127,24	136,78	149,29	153,6	162,91	164,79	166,04	202,07	204,73	207,32	208,77	211,05	217,42	225,19	232,8	228,25	227,67	237,9	257,47	253,43	245,57	241,87	244,9	248,81	247,98
Baie Chanzy	82,345	85,135	89,915	90,295	98,455	116,07	125,61	138,12	142,43	151,74	153,62	154,87	190,9	193,56	196,15	197,6	199,88	206,25	214,02	221,63	217,08	216,5	226,73	246,3	242,26	234,4	230,7	233,73	237,64	236,81
Déversoir du Lac de la Croix du Sud	77,958	80,748	85,528	85,908	94,068	111,68	121,22	133,73	138,04	147,35	149,23	150,48	186,51	189,17	191,76	193,21	195,49	201,86	209,63	217,24	212,69	212,11	222,34	241,91	237,87	230,01	226,31	229,34	233,25	232,42
Outlet of Lac Euphrosine	75,204	77,994	82,774	83,154	91,314	108,92	118,46	130,97	135,28	144,59	146,47	147,72	183,75	186,41	189	190,45	192,73	199,1	206,87	214,48	209,93	209,35	219,58	239,15	235,11	227,25	223,55	226,58	230,49	229,66
Ballon R.	74,329	77,119	81,899	82,279	90,439	108,05	117,59	130,1	134,41	143,72	145,6	146,85	182,88	185,54	188,13	189,58	191,86	198,23	206	213,61	209,06	208,48	218,71	238,28	234,24	226,38	222,68	225,71	229,62	228,79
Sans Nom R.	73,885	76,675	81,455	81,835	89,995	107,61	117,15	129,66	133,97	143,28	145,16	146,41	182,44	185,1	187,69	189,14	191,42	197,79	205,56	213,17	208,62	208,04	218,27	237,84	233,8	225,94	222,24	225,27	229,18	228,35
S. Brook Ile aux Skuas	63,998	66,788	71,568	71,948	80,108	97,718	107,26	119,77	124,08	133,39	135,27	136,52	172,55	175,21	177,8	179,25	181,53	187,9	195,67	203,28	198,73	198,15	208,38	227,95	223,91	216,05	212,35	215,38	219,29	218,46
Brook 1 Ile du Port	75,851	78,641	83,421	83,801	91,961	109,57	119,11	131,62	135,93	145,24	147,12	148,37	184,4	187,06	189,65	191,1	193,38	199,75	207,52	215,13	210,58	210	220,23	239,8	235,76	227,9	224,2	227,23	231,14	230,31
Brook 2 Ile du Port	77,425	80,215	84,995	85,375	93,535	111,15	120,69	133,2	137,51	146,82	148,7	149,95	185,98	188,64	191,23	192,68	194,96	201,33	209,1	216,71	212,16	211,58	221,81	241,38	237,34	229,48	225,78	228,81	232,72	231,89
Brook 3 Ile du Port	79,044	81,834	86,614	86,994	95,154	112,76	122,3	134,81	139,12	148,43	150,31	151,56	187,59	190,25	192,84	194,29	196,57	202,94	210,71	218,32	213,77	213,19	223,42	242,99	238,95	231,09	227,39	230,42	234,33	233,5
Brook 4 Ile du Port	79,833	82,623	87,403	87,783	95,943	113,55	123,09	135,6	139,91	149,22	151,1	152,35	188,38	191,04	193,63	195,08	197,36	203,73	211,5	219,11	214,56	213,98	224,21	243,78	239,74	231,88	228,18	231,21	235,12	234,29
Val Froide R.	76,376	79,166	83,946	84,326	92,486	110,1	119,64	132,15	136,46	145,77	147,65	148,9	184,93	187,59	190,18	191,63	193,91	200,28	208,05	215,66	211,11	210,53	220,76	240,33	236,29	228,43	224,73	227,76	231,67	230,84
Val Travers R.	70,833	73,623	78,403	78,783	86,943	104,55	114,09	126,6	130,91	140,22	142,1	143,35	179,38	182,04	184,63	186,08	188,36	194,73	202,5	210,11	205,56	204,98	215,21	234,78	230,74	222,88	219,18	222,21	226,12	225,29
Brook N. of Port-Couvreux	50,159	52,949	57,729	58,109	66,269	83,879	93,419	105,93	110,24	119,55	121,43	122,68	158,71	161,37	163,96	165,41	167,69	174,06	181,83	189,44	184,89	184,31	194,54	214,11	210,07	202,21	198,51	201,54	205,45	204,62
Brook of Port-Couvreux cabin	49,992	52,782	57,562	57,942	66,102	83,712	93,252	105,76	110,07	119,38	121,26	122,51	158,54	161,2	163,79	165,24	167,52	173,89	181,66	189,27	184,72	184,14	194,37	213,94	209,9	202,04	198,34	201,37	205,28	204,45
Brook W end of Havre du Beau Temps	59,963	62,753	67,533	67,913	76,073	93,683	103,22	115,73	120,04	129,35	131,23	132,48	168,51	171,17	173,76	175,21	177,49	183,86	191,63	199,24	194,69	194,11	204,34	223,91	219,87	212,01	208,31	211,34	215,25	214,42
Anse Sablonneuse Brook	51,128	53,918	58,698	59,078	67,238	84,848	94,388	106,9	111,21	120,52	122,4	123,65	159,68	162,34	164,93	166,38	168,66	175,03	182,8	190,41	185,86	185,28	195,51	215,08	211,04	203,18	199,48	202,51	206,42	205,59
Port Kirk R.	44,194	46,984	51,764	52,144	60,304	77,914	87,454	99,964	104,27	113,58	115,46	116,71	152,74	155,4	157,99	159,44	161,72	168,09	175,86	183,47	178,92	178,34	188,57	208,14	204,1	196,24	192,54	195,57	199,48	198,65
Val de l'Ouest R.	39,779	42,569	47,349	47,729	55,889	73,499	83,039	95,549	99,859	109,17	111,05	112,3	148,33	150,99	153,58	155,03	157,31	163,68	171,45	179,06	174,51	173,93	184,16	203,73	199,69	191,83	188,13	191,16	195,07	194,24
Studer R.	32,805	35,595	40,375	40,755	48,915	66,525	76,065	88,575	92,885	102,2	104,08	105,33	141,36	144,02	146,61	148,06	150,34	156,71	164,48	172,09	167,54	166,96	177,19	196,76	192,72	184,86	181,16	184,19	188,1	187,27
Doute R.	27,921	30,711	35,491	35,871	44,031	61,641	71,181	83,691	88,001	97,311	99,191	100,44	136,47	139,13	141,72	143,17	145,45	151,82	159,59	167,2	162,65	162,07	172,3	191,87	187,83	179,97	176,27	179,3	183,21	182,38
Charbon R.	28,274	31,064	35,844	36,224	44,384	61,994	71,534	84,044	88,354	97,664	99,544	100,79	136,82	139,48	142,07	143,52	145,8	152,17	159,94	167,55	163	162,42	172,65	192,22	188,18	180,32	176,62	179,65	183,56	182,73
Sérail R.	22,518	25,308	30,088	30,468	38,628	56,238	65,778	78,288	82,598	91,908	93,788	95,038	131,07	133,73	136,32	137,77	140,05	146,42	154,19	161,8	157,25	156,67	166,9	186,47	182,43	174,57	170,87	173,9	177,81	176,98
Chasseurs R.	18,108	20,898	25,678	26,058	34,218	51,828	61,368	73,878	78,188	87,498	89,378	90,628	126,66	129,32	131,91	133,36	135,64	142,01	149,78	157,39	152,84	152,26	162,49	182,06	178,02	170,16	166,46	169,49	173,4	172,57
Nord R.	9,18	12,01	16,82	17,34	25,543	43,153	52,693	65,203	69,513	78,823	80,703	81,953	117,98	120,64	123,23	124,68	126,96	133,33	141,1	148,71	144,16	143,58	153,81	173,38	169,34	161,48	157,78	160,81	164,72	163,89
Pépins R.	4,73	7,36	12,44	12,89	20,977	38,587	48,127	60,637	64,947	74,257	76,137	77,387	113,42	116,08	118,67	120,12	122,4	128,77	136,54	144,15	139,6	139,02	149,25	168,82	164,78	156,92	153,22	156,25	160,16	159,33
Cataractes R.	2,05	4,87	9,95	10,28	18,578	36,188	45,728	58,238	62,548	71,858	73,738	74,988	111,02	113,68	116,27	117,72	120	126,37	134,14	141,75	137,2	136,62	146,85	166,42	162,38	154,52	150,82	153,85	157,76	156,93
Hautes Mares South Brook (Gorfous 1)	0,44	3,8	8,41	8,88	17,351	34,961	44,501	57,011	61,321	70,631	72,511	73,761	109,79	112,45	115,04	116,49	118,77	125,14	132,91	140,52	135,97	135,39	145,62	165,19	161,15	153,29	149,59	152,62	156,53	155,7
Hautes Mares North Brook (Gorfous 2)	0	3,57	8,44	8,64	16,972	34,582	44,122	56,632	60,942	70,252	72,132	73,382	109,41	112,07	114,66	116,11	118,39	124,76	132,53	140,14	135,59	135,01	145,24	164,81	160,77	152,91	149,21	152,24	156,15	155,32
Pointe des Cabanes Brook (Gorfous 4)	3,57	0	5,04	5,43	13,741	31,351	40,891	53,401	57,711	67,021	68,901	70,151	106,18	108,84	111,43	112,88	115,16	121,53	129,3	136,91	132,36	131,78	142,01	161,58	157,54	149,68	145,98	149,01	152,92	152,09
Cap Rouge Brook (Gorfous 5)	8,44	5,04	0	0,96	9,602	27,212	36,752	49,262	53,572	62,882	64,762	66,012	102,04	104,7	107,29	108,74	111,02	117,39	125,16	132,77	128,22	127,64	137,87	157,44	153,4	145,54	141,84	144,87	148,78	147,95
N. Cap Rouge Brook (Gorfous 6)	8,64	5,43	0,96	0	8,777	26,387	35,927	48,437	52,747	62,057	63,937	65,187	101,22	103,88	106,47	107,92	110,2	116,57	124,34	131,95	127,4	126,82	137,05	156,62	152,58	144,72	141,02	144,05	147,96	147,13
Cap de Rohan R.	16,972	13,741	9,602	8,777	0	17,601	27,141	39,651	43,961	53,271	55,151	56,401	92,431	95,091	97,681	99,131	101,41	107,78	115,55	123,16	118,61	118,03	128,26	147,83	143,79	135,93	132,23	135,26	139,17	138,34
Lac Marville Outlet	34,582	31,351	27,212	26,387	17,601	0	13,305	25,815	30,125	39,435	41,315	42,565	78,595	81,255	83,845	85,295	87,575	93,945	101,72	109,33	104,78	104,2	114,43	134	129,96	122,1	118,4	121,43	125,34	124,51
Manchots R.	44,122	40,891	36,752	35,927	27,141	13,305	0	12,549	16,859	26,169	28,049	29,299	65,329	67,989	70,579	72,029	74,309	80,679	88,449	96,059	91,509	90,929	101,16	120,73	116,69	108,83	105,13	108,16	112,07	111,24
Calcédoines R.	56,632	53,401	49,262	48,437	39,651	25,815	12,549	0	4,695	14,005	15,885	17,135	53,165	55,825	58,415	59,865	62,145	68,515	76,285	83,895	79,345	78,765	88,995	108,57	104,53	96,665	92,965	95,995	99,905	99,075
Bungay R.	60,942	57,711	53,572	52,747	43,961	30,125	16,859	4,695	0	9,755	11,635	12,885	48,915	51,575	54,165	55,615	57,895	64,265	72,035	79,645	75,095	74,515	84,745	104,32	100,28	92,415	88,715	91,745	95,655	94,825
Albatros R.	70,252	67,021	62,882	62,057	53,271	39,435	26,169	14,005	9,755	0	2,76	4,01	40,04	42,7	45,29	46,74	49,02	55,39	63,16	70,77	66,22	65,64	75,87	95,44	91,4	83,54	79,84	82,87	86,78	85,95
Norvégienne R.	72,132	68,901	64,762	63,937	55,151	41,315	28,049	15,885	11,635	2,76	0	2,259	38,289	40,949	43,539	44,989	47,269	53,639	61,409	69,019	64,469	63,889	74,119	93,689	89,649	81,789	78,089	81,119	85,029	84,199
Château R.	73,382	70,151	66,012	65,187	56,401	42,565	29,299	17,135	12,885	4,01	2,259	0	37,53	40,19	42,78	44,23	46,51	52,88	60,65	68,26	63,71	63,13	73,36	92,93	88,89	81,03	77,33	80,36	84,27	83,44
PAF Ferme R.	109,41	106,18	102,04	101,22	92,431	78,595	65,329	53,165	48,915	40,04	38,289	37,53	0	4,29	6,98	8,51	10,74	17,186	24,956	32,566	28,016	27,436	37,666	57,236	53,196	45,336	41,636	44,666	48,576	47,746
Borgne R.	112,07	108,84	104,7	103,88	95,091	81,255	67,989	55,825	51,575	42,7	40,949	40,19	4,29	0	2,89	4,33	6,66	13,084	20,854	28,464	23,914	23,334	33,564	53,134	49,094	41,234	37,534	40,564	44,474	43,644
Sud R.	114,66	111,43	107,29	106,47	97,681	83,845	70,579	58,415	54,165	45,29	43,539	42,78	6,98	2,89	0	1,47	3,86	10,358	18,128	25,738	21,188	20,608	30,838	50,408	46,368	38,508	34,808	37,838	41,748	40,918
Pointe de l'épave Brook	116,11	112,88	108,74	107,92	99,131	85,295	72,029	59,865	55,615	46,74	44,989	44,23	8,51	4,33	1,47	0	2,64	9,149	16,919	24,529	19,979	19,399	29,629	49,199	45,159	37,299	33,599	36,629	40,539	39,709
Américains R.	118,39	115,16	111,02	110,2	101,41	87,575	74,309	62,145	57,895	49,02	47,269	46,51	10,74	6,66	3,86	2,64	0	7,421	15,191	22,801	18,251	17,671	27,901	47,471	43,431	35,571	31,871	34,901	38,811	37,981
3 Lacs R.	124,76	121,53	117,39	116,57	107,78	93,945	80,679	68,515	64,265	55,39	53,639	52,88	17,186	13,084	10,358	9,149	7,421	0	8,3	15,91	11,73	11,04	22,4	42,811	38,04	30,15	26,22	29,15	33,23	32,52
Val d'Auge R.	132,53	129,3	125,16	124,34	115,55	101,72	88,449	76,285	72,035	63,16	61,409	60,65	24,956	20,854	18,128	16,919	15,191	8,3	0	8,1	12,32	11,82	24,54	44,95	40,1	32,29	28,36	31,29	35,38	36,62
Anse de St Malo R.	140,14	136,91	132,77	131,95	123,16	109,33	96,059	83,895	79,645	70,77	69,019	68,26	32,566	28,464	25,738	24,529	22,801	15,91	8,1	0	15,37	14,99	27,71	48,23	43,22	35,57	31,65	34,56	38,66	42,91
Basin downstream of Korrigans Lake	135,59	132,36	128,22	127,4	118,61	104,78	91,509	79,345	75,095	66,22	64,469	63,71	28,016	23,914	21,188	19,979	18,251	11,73	12,32	15,37	0	2,42	18,1	38,52	33,61	25,86	21,94	24,84	28,95	32,73
Baie de l'Observatoire R.	135,01	131,78	127,64	126,82	118,03	104,2	90,929	78,765	74,515	65,64	63,889	63,13	27,436	23,334	20,608	19,399	17,671	11,04	11,82	14,99	2,42	0	16,75	37,15	32,16	24,49	20,56	23,49	27,58	31,36
SW Brook on Bras Jules Laboureur	145,24	142,01	137,87	137,05	128,26	114,43	101,16	88,995	84,745	75,87	74,119	73,36	37,666	33,564	30,838	29,629	27,901	22,4	24,54	27,71	18,1	16,75	0	28,89	22,92	15,43	13,34	18,38	22,58	28,59
Grisanche R.	164,81	161,58	157,44	156,62	147,83	134	120,73	108,57	104,32	95,44	93,689	92,93	57,236	53,134	50,408	49,199	47,471	42,811	44,95	48,23	38,52	37,15	28,89	0	4,97	18,44	21,12	26,79	31,57	37,61
Lac des Fougères Outlet	160,77	157,54	153,4	152,58	143,79	129,96	116,69	104,53	100,28	91,4	89,649	88,89	53,196	49,094	46,368	45,159	43,431	38,04	40,1	43,22	33,61	32,16	22,92	4,97	0	13,47	16,15	21,82	26,6	32,64
Lac d'Armor Basin	152,91	149,68	145,54	144,72	135,93	122,1	108,83	96,665	92,415	83,54	81,789	81,03	45,336	41,234	38,508	37,299	35,571	30,15	32,29	35,57	25,86	24,49	15,43	18,44	13,47	0	7,44	18,13	22,68	28,78
Anse J. Bourcart Brook	149,21	145,98	141,84	141,02	132,23	118,4	105,13	92,965	88,715	79,84	78,089	77,33	41,636	37,534	34,808	33,599	31,871	26,22	28,36	31,65	21,94	20,56	13,34	21,12	16,15	7,44	0	11,01	15,86	22,01
Ravin du Charbon R.	152,24	149,01	144,87	144,05	135,26	121,43	108,16	95,995	91,745	82,87	81,119	80,36	44,666	40,564	37,838	36,629	34,901	29,15	31,29	34,56	24,84	23,49	18,38	26,79	21,82	18,13	11,01	0	4,54	10,61
Vallée des Neiges Basin	156,15	152,92	148,78	147,96	139,17	125,34	112,07	99,905	95,655	86,78	85,029	84,27	48,576	44,474	41,748	40,539	38,811	33,23	35,38	38,66	28,95	27,58	22,58	31,57	26,6	22,68	15,86	4,54	0	6,16
Acaena R.	155,32	152,09	147,95	147,13	138,34	124,51	111,24	99,075	94,825	85,95	84,199	83,44	47,746	43,644	40,918	39,709	37,981	32,52	36,62	42,91	32,73	31,36	28,59	37,61	32,64	28,78	22,01	10,61	6,16	0
Vallée de la Planchette R.	152,58	149,35	145,21	144,39	135,6	121,77	108,5	96,335	92,085	83,21	81,459	80,7	45,006	40,904	38,178	36,969	35,241	29,92	34,02	40,24	31,26	29,99	31,22	40,63	35,66	27,33	23,28	15,91	11,54	6,55
Val Raide Brook	160,58	157,35	153,21	152,39	143,6	129,77	116,5	104,34	100,09	91,21	89,459	88,7	53,006	48,904	46,178	44,969	43,241	37,81	42,02	48,31	39,33	38,03	39,64	48,89	43,92	36,07	29,61	24,6	20,19	15,08
Mouettes R.	153,54	150,31	146,17	145,35	136,56	122,73	109,46	97,295	93,045	84,17	82,419	81,66	45,966	41,864	39,138	37,929	36,201	30,89	34,99	41,29	32,31	31,01	32,66	41,86	36,89	29,42	22,6	17,96	13,46	8,38
Macaronis R.	165,99	162,76	158,62	157,8	149,01	135,18	121,91	109,75	105,5	96,62	94,869	94,11	58,416	54,314	51,588	50,379	48,651	43,19	47,29	53,6	44,62	43,31	48,86	64,59	59,62	51,27	47,54	48,47	44,04	39,16
Vallée Phonolite Basin	178,04	174,81	170,67	169,85	161,06	147,23	133,96	121,8	117,55	108,67	106,92	106,16	70,466	66,364	63,638	62,429	60,701	55,24	59,34	65,65	56,67	55,36	60,91	76,64	71,67	63,32	59,59	60,52	56,09	51,21
Val du Levant R.	191,3	188,07	183,93	183,11	174,32	160,49	147,22	135,06	130,81	121,93	120,18	119,42	83,726	79,624	76,898	75,689	73,961	68,5	72,6	78,91	69,93	68,62	74,17	89,9	84,93	76,58	72,85	73,78	69,35	64,47
Anse de la Canicule R.	218,94	215,71	211,57	210,75	201,96	188,13	174,86	162,7	158,45	149,57	147,82	147,06	111,37	107,26	104,54	103,33	101,6	96,14	100,24	106,55	97,57	96,26	101,81	117,54	112,57	104,22	100,49	101,42	96,99	92,11
Crête des Laves R.	221,06	217,83	213,69	212,87	204,08	190,25	176,98	164,82	160,57	151,69	149,94	149,18	113,49	109,38	106,66	105,45	103,72	98,26	102,36	108,67	99,69	98,38	103,93	119,66	114,69	106,34	102,61	103,54	99,11	94,23
Halage des Swains R.	232,05	228,82	224,68	223,86	215,07	201,24	187,97	175,81	171,56	162,68	160,93	160,17	124,48	120,37	117,65	116,44	114,71	109,25	113,35	119,66	110,68	109,37	114,92	130,65	125,68	117,33	113,6	114,53	110,1	105,22
Northern arm of Anse aux Ecueils Brook	241,16	237,93	233,79	232,97	224,18	210,35	197,08	184,92	180,67	171,79	170,04	169,28	133,59	129,48	126,76	125,55	123,82	118,36	122,46	128,77	119,79	118,48	124,03	139,76	134,79	126,44	122,71	123,64	119,21	114,33
La Clarée Basin: Hermance Lake & Galets R.	241,44	238,21	234,07	233,25	224,46	210,63	197,36	185,2	180,95	172,07	170,32	169,56	133,87	129,76	127,04	125,83	124,1	118,64	122,74	129,05	120,07	118,76	124,31	140,04	135,07	126,72	122,99	123,92	119,49	114,61
Olsen R.	239,73	236,5	232,36	231,54	222,75	208,92	195,65	183,49	179,24	170,36	168,61	167,85	132,16	128,05	125,33	124,12	122,39	116,93	121,03	127,34	118,36	117,05	122,6	138,33	133,36	125,01	121,28	122,21	117,78	112,9
Vallée du Radioleine R.	235,77	232,54	228,4	227,58	218,79	204,96	191,69	179,53	175,28	166,4	164,65	163,89	128,2	124,09	121,37	120,16	118,43	112,97	117,07	123,38	114,4	113,09	118,64	134,37	129,4	121,05	117,32	118,25	113,82	108,94
Val des Skuas R.	244,03	240,8	236,66	235,84	227,05	213,22	199,95	187,79	183,54	174,66	172,91	172,15	136,46	132,35	129,63	128,42	126,69	121,23	125,33	131,64	122,66	121,35	126,9	142,63	137,66	129,31	125,58	126,51	122,08	117,2
Doigt de Sainte-Anne R.	263,31	260,08	255,94	255,11	246,33	232,49	219,23	207,06	202,81	193,94	192,19	191,43	155,73	151,63	148,91	147,7	145,97	140,51	144,61	150,92	141,94	140,63	146,18	161,91	156,94	148,59	144,86	145,79	141,36	136,48
Plaine de Dante R.	265,57	262,34	258,2	257,37	248,59	234,75	221,49	209,32	205,07	196,2	194,45	193,69	157,99	153,89	151,16	149,96	148,23	142,77	146,87	153,18	144,2	142,89	148,44	164,17	159,2	150,85	147,12	148,05	143,62	138,74
Mont Ventoux R.	91,406	94,196	98,976	99,356	107,52	125,13	134,67	147,18	151,49	160,8	162,68	163,93	199,96	202,62	205,21	206,66	208,94	215,31	223,08	230,69	226,14	225,56	235,79	255,36	251,32	243,46	239,76	242,79	246,7	245,87
Mont Sinaï R.	87,276	90,066	94,846	95,226	103,39	121	130,54	143,05	147,36	156,67	158,55	159,8	195,83	198,49	201,08	202,53	204,81	211,18	218,95	226,56	222,01	221,43	231,66	251,23	247,19	239,33	235,63	238,66	242,57	241,74
Bastille R.	110,09	112,88	117,66	118,04	126,2	143,81	153,35	165,86	170,17	179,48	181,36	182,61	218,64	221,3	223,89	225,34	227,62	233,99	241,76	249,37	244,82	244,24	254,47	274,04	270	262,14	258,44	261,47	265,38	264,55
Lac Mariot outlet	279,9	276,67	272,53	271,71	262,92	249,09	235,82	223,66	219,41	210,53	208,78	208,02	172,33	168,22	165,5	164,29	162,56	157,1	161,2	167,51	158,53	157,22	162,77	178,5	173,53	165,18	161,45	162,38	157,95	153,07
Plaine d'Ampère R.	282,56	279,33	275,19	274,37	265,58	251,75	238,48	226,32	222,07	213,19	211,44	210,68	174,99	170,88	168,16	166,95	165,22	159,76	163,86	170,17	161,19	159,88	165,43	181,16	176,19	167,84	164,11	165,04	160,61	155,73
Portes Noires R.	291,23	288	283,86	283,04	274,25	260,42	247,15	234,99	230,74	221,86	220,11	219,35	183,66	179,55	176,83	175,62	173,89	168,43	172,53	178,84	169,86	168,55	174,1	189,83	184,86	176,51	172,78	173,71	169,28	164,4
Contacts R.	315,64	312,41	308,27	307,45	298,66	284,83	271,56	259,4	255,15	246,27	244,52	243,76	208,07	203,96	201,24	200,03	198,3	192,84	196,94	203,25	194,27	192,96	198,51	214,24	209,27	200,92	197,19	198,12	193,69	188,81
Mouche R.	311,2	307,97	303,83	303,01	294,22	280,39	267,12	254,96	250,71	241,83	240,08	239,32	203,63	199,52	196,8	195,59	193,86	188,4	192,5	198,81	189,83	188,52	194,07	209,8	204,83	196,48	192,75	193,68	189,25	184,37
Larmor R.	305,49	302,26	298,12	297,3	288,51	274,68	261,41	249,25	245	236,12	234,37	233,61	197,92	193,81	191,09	189,88	188,15	182,69	186,79	193,1	184,12	182,81	188,36	204,09	199,12	190,77	187,04	187,97	183,54	178,66
Plage Jaune R.	314,42	311,19	307,05	306,23	297,44	283,61	270,34	258,18	253,93	245,05	243,3	242,54	206,85	202,74	200,02	198,81	197,08	191,62	195,72	202,03	193,05	191,74	197,29	213,02	208,05	199,7	195,97	196,9	192,47	187,59
Sables R.	321,01	317,78	313,64	312,82	304,03	290,2	276,93	264,77	260,52	251,64	249,89	249,13	213,44	209,33	206,61	205,4	203,67	198,21	202,31	208,62	199,64	198,33	203,88	219,61	214,64	206,29	202,56	203,49	199,06	194,18

Patch names	Vallée de la Planchette R.	Val Raide Brook	Mouettes R.	Macaronis R.	Vallée Phonolite Basin	Val du Levant R.	Anse de la Canicule R.	Crête des Laves R.	Halage des Swains R.	Northern arm of Anse aux Ecueils Brook	La Clarée Basin: Hermance Lake & Galets R.	Olsen R.	Vallée du Radioleine R.	Val des Skuas R.	Doigt de Sainte-Anne R.	Plaine de Dante R.	Mont Ventoux R.	Mont Sinaï R.	Bastille R.	Lac Mariot outlet	Plaine d'Ampère R.	Portes Noires R.	Contacts R.	Mouche R.	Larmor R.	Plage Jaune R.	Sables R.
Entrelacs R.	245,24	253,24	246,2	258,65	270,7	283,96	311,6	313,72	324,71	333,82	334,1	332,39	328,43	336,69	355,97	358,23	2,67	8,93	45,54	372,56	375,22	383,89	408,3	403,86	398,15	407,08	413,67
Baie Chanzy	234,07	242,07	235,03	247,48	259,53	272,79	300,43	302,55	313,54	322,65	322,93	321,22	317,26	325,52	344,79	347,05	22,716	18,586	41,401	361,39	364,05	372,72	397,13	392,69	386,98	395,91	402,5
Déversoir du Lac de la Croix du Sud	229,68	237,68	230,64	243,09	255,14	268,4	296,04	298,16	309,15	318,26	318,54	316,83	312,87	321,13	340,41	342,66	22,916	18,786	41,601	357	359,66	368,33	392,74	388,3	382,59	391,52	398,11
Outlet of Lac Euphrosine	226,92	234,92	227,88	240,33	252,38	265,64	293,28	295,4	306,39	315,5	315,78	314,07	310,11	318,37	337,65	339,91	30,256	26,126	48,941	354,24	356,9	365,57	389,98	385,54	379,83	388,76	395,35
Ballon R.	226,05	234,05	227,01	239,46	251,51	264,77	292,41	294,53	305,52	314,63	314,91	313,2	309,24	317,5	336,78	339,04	29,636	25,506	48,321	353,37	356,03	364,7	389,11	384,67	378,96	387,89	394,48
Sans Nom R.	225,61	233,61	226,57	239,02	251,07	264,33	291,97	294,09	305,08	314,19	314,47	312,76	308,8	317,06	336,33	338,59	29,056	24,926	47,741	352,93	355,59	364,26	388,67	384,23	378,52	387,45	394,04
S. Brook Ile aux Skuas	215,72	223,72	216,68	229,13	241,18	254,44	282,08	284,2	295,19	304,3	304,58	302,87	298,91	307,17	326,45	328,7	28,836	24,706	47,521	343,04	345,7	354,37	378,78	374,34	368,63	377,56	384,15
Brook 1 Ile du Port	227,57	235,57	228,53	240,98	253,03	266,29	293,93	296,05	307,04	316,15	316,43	314,72	310,76	319,02	338,3	340,56	31,136	27,006	49,821	354,89	357,55	366,22	390,63	386,19	380,48	389,41	396
Brook 2 Ile du Port	229,15	237,15	230,11	242,56	254,61	267,87	295,51	297,63	308,62	317,73	318,01	316,3	312,34	320,6	339,87	342,13	32,006	27,876	50,691	356,47	359,13	367,8	392,21	387,77	382,06	390,99	397,58
Brook 3 Ile du Port	230,76	238,76	231,72	244,17	256,22	269,48	297,12	299,24	310,23	319,34	319,62	317,91	313,95	322,21	341,49	343,75	33,416	29,286	52,101	358,08	360,74	369,41	393,82	389,38	383,67	392,6	399,19
Brook 4 Ile du Port	231,55	239,55	232,51	244,96	257,01	270,27	297,91	300,03	311,02	320,13	320,41	318,7	314,74	323	342,28	344,54	34,126	29,996	52,811	358,87	361,53	370,2	394,61	390,17	384,46	393,39	399,98
Val Froide R.	228,1	236,1	229,06	241,51	253,56	266,82	294,46	296,58	307,57	316,68	316,96	315,25	311,29	319,55	338,82	341,08	37,116	32,986	55,801	355,42	358,08	366,75	391,16	386,72	381,01	389,94	396,53
Val Travers R.	222,55	230,55	223,51	235,96	248,01	261,27	288,91	291,03	302,02	311,13	311,41	309,7	305,74	314	333,28	335,54	31,966	27,836	50,651	349,87	352,53	361,2	385,61	381,17	375,46	384,39	390,98
Brook N. of Port-Couvreux	201,88	209,88	202,84	215,29	227,34	240,6	268,24	270,36	281,35	290,46	290,74	289,03	285,07	293,33	312,61	314,87	44,796	40,666	63,481	329,2	331,86	340,53	364,94	360,5	354,79	363,72	370,31
Brook of Port-Couvreux cabin	201,71	209,71	202,67	215,12	227,17	240,43	268,07	270,19	281,18	290,29	290,57	288,86	284,9	293,16	312,44	314,7	45,706	41,576	64,391	329,03	331,69	340,36	364,77	360,33	354,62	363,55	370,14
Brook W end of Havre du Beau Temps	211,68	219,68	212,64	225,09	237,14	250,4	278,04	280,16	291,15	300,26	300,54	298,83	294,87	303,13	322,41	324,67	56,186	52,056	74,871	339	341,66	350,33	374,74	370,3	364,59	373,52	380,11
Anse Sablonneuse Brook	202,85	210,85	203,81	216,26	228,31	241,57	269,21	271,33	282,32	291,43	291,71	290	286,04	294,3	313,58	315,83	47,156	43,026	65,841	330,17	332,83	341,5	365,91	361,47	355,76	364,69	371,28
Port Kirk R.	195,91	203,91	196,87	209,32	221,37	234,63	262,27	264,39	275,38	284,49	284,77	283,06	279,1	287,36	306,64	308,9	54,066	49,936	72,751	323,23	325,89	334,56	358,97	354,53	348,82	357,75	364,34
Val de l'Ouest R.	191,5	199,5	192,46	204,91	216,96	230,22	257,86	259,98	270,97	280,08	280,36	278,65	274,69	282,95	302,23	304,49	54,286	50,156	72,971	318,82	321,48	330,15	354,56	350,12	344,41	353,34	359,93
Studer R.	184,53	192,53	185,49	197,94	209,99	223,25	250,89	253,01	264	273,11	273,39	271,68	267,72	275,98	295,25	297,51	60,636	56,506	79,321	311,85	314,51	323,18	347,59	343,15	337,44	346,37	352,96
Doute R.	179,64	187,64	180,6	193,05	205,1	218,36	246	248,12	259,11	268,22	268,5	266,79	262,83	271,09	290,37	292,63	66,016	61,886	84,701	306,96	309,62	318,29	342,7	338,26	332,55	341,48	348,07
Charbon R.	179,99	187,99	180,95	193,4	205,45	218,71	246,35	248,47	259,46	268,57	268,85	267,14	263,18	271,44	290,72	292,98	66,246	62,116	84,931	307,31	309,97	318,64	343,05	338,61	332,9	341,83	348,42
Sérail R.	174,24	182,24	175,2	187,65	199,7	212,96	240,6	242,72	253,71	262,82	263,1	261,39	257,43	265,69	284,97	287,22	70,506	66,376	89,191	301,56	304,22	312,89	337,3	332,86	327,15	336,08	342,67
Chasseurs R.	169,83	177,83	170,79	183,24	195,29	208,55	236,19	238,31	249,3	258,41	258,69	256,98	253,02	261,28	280,56	282,81	75,306	71,176	93,991	297,15	299,81	308,48	332,89	328,45	322,74	331,67	338,26
Nord R.	161,15	169,15	162,11	174,56	186,61	199,87	227,51	229,63	240,62	249,73	250,01	248,3	244,34	252,6	271,88	274,14	88,716	84,586	107,4	288,47	291,13	299,8	324,21	319,77	314,06	322,99	329,58
Pépins R.	156,59	164,59	157,55	170	182,05	195,31	222,95	225,07	236,06	245,17	245,45	243,74	239,78	248,04	267,31	269,57	90,136	86,006	108,82	283,91	286,57	295,24	319,65	315,21	309,5	318,43	325,02
Cataractes R.	154,19	162,19	155,15	167,6	179,65	192,91	220,55	222,67	233,66	242,77	243,05	241,34	237,38	245,64	264,92	267,17	90,356	86,226	109,04	281,51	284,17	292,84	317,25	312,81	307,1	316,03	322,62
Hautes Mares South Brook (Gorfous 1)	152,96	160,96	153,92	166,37	178,42	191,68	219,32	221,44	232,43	241,54	241,82	240,11	236,15	244,41	263,69	265,95	91,256	87,126	109,94	280,28	282,94	291,61	316,02	311,58	305,87	314,8	321,39
Hautes Mares North Brook (Gorfous 2)	152,58	160,58	153,54	165,99	178,04	191,3	218,94	221,06	232,05	241,16	241,44	239,73	235,77	244,03	263,31	265,57	91,406	87,276	110,09	279,9	282,56	291,23	315,64	311,2	305,49	314,42	321,01
Pointe des Cabanes Brook (Gorfous 4)	149,35	157,35	150,31	162,76	174,81	188,07	215,71	217,83	228,82	237,93	238,21	236,5	232,54	240,8	260,08	262,34	94,196	90,066	112,88	276,67	279,33	288	312,41	307,97	302,26	311,19	317,78
Cap Rouge Brook (Gorfous 5)	145,21	153,21	146,17	158,62	170,67	183,93	211,57	213,69	224,68	233,79	234,07	232,36	228,4	236,66	255,94	258,2	98,976	94,846	117,66	272,53	275,19	283,86	308,27	303,83	298,12	307,05	313,64
N. Cap Rouge Brook (Gorfous 6)	144,39	152,39	145,35	157,8	169,85	183,11	210,75	212,87	223,86	232,97	233,25	231,54	227,58	235,84	255,11	257,37	99,356	95,226	118,04	271,71	274,37	283,04	307,45	303,01	297,3	306,23	312,82
Cap de Rohan R.	135,6	143,6	136,56	149,01	161,06	174,32	201,96	204,08	215,07	224,18	224,46	222,75	218,79	227,05	246,33	248,59	107,52	103,39	126,2	262,92	265,58	274,25	298,66	294,22	288,51	297,44	304,03
Lac Marville Outlet	121,77	129,77	122,73	135,18	147,23	160,49	188,13	190,25	201,24	210,35	210,63	208,92	204,96	213,22	232,49	234,75	125,13	121	143,81	249,09	251,75	260,42	284,83	280,39	274,68	283,61	290,2
Manchots R.	108,5	116,5	109,46	121,91	133,96	147,22	174,86	176,98	187,97	197,08	197,36	195,65	191,69	199,95	219,23	221,49	134,67	130,54	153,35	235,82	238,48	247,15	271,56	267,12	261,41	270,34	276,93
Calcédoines R.	96,335	104,34	97,295	109,75	121,8	135,06	162,7	164,82	175,81	184,92	185,2	183,49	179,53	187,79	207,06	209,32	147,18	143,05	165,86	223,66	226,32	234,99	259,4	254,96	249,25	258,18	264,77
Bungay R.	92,085	100,09	93,045	105,5	117,55	130,81	158,45	160,57	171,56	180,67	180,95	179,24	175,28	183,54	202,81	205,07	151,49	147,36	170,17	219,41	222,07	230,74	255,15	250,71	245	253,93	260,52
Albatros R.	83,21	91,21	84,17	96,62	108,67	121,93	149,57	151,69	162,68	171,79	172,07	170,36	166,4	174,66	193,94	196,2	160,8	156,67	179,48	210,53	213,19	221,86	246,27	241,83	236,12	245,05	251,64
Norvégienne R.	81,459	89,459	82,419	94,869	106,92	120,18	147,82	149,94	160,93	170,04	170,32	168,61	164,65	172,91	192,19	194,45	162,68	158,55	181,36	208,78	211,44	220,11	244,52	240,08	234,37	243,3	249,89
Château R.	80,7	88,7	81,66	94,11	106,16	119,42	147,06	149,18	160,17	169,28	169,56	167,85	163,89	172,15	191,43	193,69	163,93	159,8	182,61	208,02	210,68	219,35	243,76	239,32	233,61	242,54	249,13
PAF Ferme R.	45,006	53,006	45,966	58,416	70,466	83,726	111,37	113,49	124,48	133,59	133,87	132,16	128,2	136,46	155,73	157,99	199,96	195,83	218,64	172,33	174,99	183,66	208,07	203,63	197,92	206,85	213,44
Borgne R.	40,904	48,904	41,864	54,314	66,364	79,624	107,26	109,38	120,37	129,48	129,76	128,05	124,09	132,35	151,63	153,89	202,62	198,49	221,3	168,22	170,88	179,55	203,96	199,52	193,81	202,74	209,33
Sud R.	38,178	46,178	39,138	51,588	63,638	76,898	104,54	106,66	117,65	126,76	127,04	125,33	121,37	129,63	148,91	151,16	205,21	201,08	223,89	165,5	168,16	176,83	201,24	196,8	191,09	200,02	206,61
Pointe de l'épave Brook	36,969	44,969	37,929	50,379	62,429	75,689	103,33	105,45	116,44	125,55	125,83	124,12	120,16	128,42	147,7	149,96	206,66	202,53	225,34	164,29	166,95	175,62	200,03	195,59	189,88	198,81	205,4
Américains R.	35,241	43,241	36,201	48,651	60,701	73,961	101,6	103,72	114,71	123,82	124,1	122,39	118,43	126,69	145,97	148,23	208,94	204,81	227,62	162,56	165,22	173,89	198,3	193,86	188,15	197,08	203,67
3 Lacs R.	29,92	37,81	30,89	43,19	55,24	68,5	96,14	98,26	109,25	118,36	118,64	116,93	112,97	121,23	140,51	142,77	215,31	211,18	233,99	157,1	159,76	168,43	192,84	188,4	182,69	191,62	198,21
Val d'Auge R.	34,02	42,02	34,99	47,29	59,34	72,6	100,24	102,36	113,35	122,46	122,74	121,03	117,07	125,33	144,61	146,87	223,08	218,95	241,76	161,2	163,86	172,53	196,94	192,5	186,79	195,72	202,31
Anse de St Malo R.	40,24	48,31	41,29	53,6	65,65	78,91	106,55	108,67	119,66	128,77	129,05	127,34	123,38	131,64	150,92	153,18	230,69	226,56	249,37	167,51	170,17	178,84	203,25	198,81	193,1	202,03	208,62
Basin downstream of Korrigans Lake	31,26	39,33	32,31	44,62	56,67	69,93	97,57	99,69	110,68	119,79	120,07	118,36	114,4	122,66	141,94	144,2	226,14	222,01	244,82	158,53	161,19	169,86	194,27	189,83	184,12	193,05	199,64
Baie de l'Observatoire R.	29,99	38,03	31,01	43,31	55,36	68,62	96,26	98,38	109,37	118,48	118,76	117,05	113,09	121,35	140,63	142,89	225,56	221,43	244,24	157,22	159,88	168,55	192,96	188,52	182,81	191,74	198,33
SW Brook on Bras Jules Laboureur	31,22	39,64	32,66	48,86	60,91	74,17	101,81	103,93	114,92	124,03	124,31	122,6	118,64	126,9	146,18	148,44	235,79	231,66	254,47	162,77	165,43	174,1	198,51	194,07	188,36	197,29	203,88
Grisanche R.	40,63	48,89	41,86	64,59	76,64	89,9	117,54	119,66	130,65	139,76	140,04	138,33	134,37	142,63	161,91	164,17	255,36	251,23	274,04	178,5	181,16	189,83	214,24	209,8	204,09	213,02	219,61
Lac des Fougères Outlet	35,66	43,92	36,89	59,62	71,67	84,93	112,57	114,69	125,68	134,79	135,07	133,36	129,4	137,66	156,94	159,2	251,32	247,19	270	173,53	176,19	184,86	209,27	204,83	199,12	208,05	214,64
Lac d'Armor Basin	27,33	36,07	29,42	51,27	63,32	76,58	104,22	106,34	117,33	126,44	126,72	125,01	121,05	129,31	148,59	150,85	243,46	239,33	262,14	165,18	167,84	176,51	200,92	196,48	190,77	199,7	206,29
Anse J. Bourcart Brook	23,28	29,61	22,6	47,54	59,59	72,85	100,49	102,61	113,6	122,71	122,99	121,28	117,32	125,58	144,86	147,12	239,76	235,63	258,44	161,45	164,11	172,78	197,19	192,75	187,04	195,97	202,56
Ravin du Charbon R.	15,91	24,6	17,96	48,47	60,52	73,78	101,42	103,54	114,53	123,64	123,92	122,21	118,25	126,51	145,79	148,05	242,79	238,66	261,47	162,38	165,04	173,71	198,12	193,68	187,97	196,9	203,49
Vallée des Neiges Basin	11,54	20,19	13,46	44,04	56,09	69,35	96,99	99,11	110,1	119,21	119,49	117,78	113,82	122,08	141,36	143,62	246,7	242,57	265,38	157,95	160,61	169,28	193,69	189,25	183,54	192,47	199,06
Acaena R.	6,55	15,08	8,38	39,16	51,21	64,47	92,11	94,23	105,22	114,33	114,61	112,9	108,94	117,2	136,48	138,74	245,87	241,74	264,55	153,07	155,73	164,4	188,81	184,37	178,66	187,59	194,18
Vallée de la Planchette R.	0	9,09	2,67	36,26	48,31	61,57	89,21	91,33	102,32	111,43	111,71	110	106,04	114,3	133,58	135,84	243,13	239	261,81	150,17	152,83	161,5	185,91	181,47	175,76	184,69	191,28
Val Raide Brook	9,09	0	7,07	44,46	56,51	69,77	97,41	99,53	110,52	119,63	119,91	118,2	114,24	122,5	141,78	144,04	251,13	247	269,81	158,37	161,03	169,7	194,11	189,67	183,96	192,89	199,48
Mouettes R.	2,67	7,07	0	37,52	49,57	62,83	90,47	92,59	103,58	112,69	112,97	111,26	107,3	115,56	134,84	137,1	244,09	239,96	262,77	151,43	154,09	162,76	187,17	182,73	177,02	185,95	192,54
Macaronis R.	36,26	44,46	37,52	0	13,57	26,83	54,47	56,59	67,58	76,69	76,97	75,26	71,3	79,56	98,837	101,1	256,54	252,41	275,22	115,43	118,09	126,76	151,17	146,73	141,02	149,95	156,54
Vallée Phonolite Basin	48,31	56,51	49,57	13,57	0	20,75	48,39	50,51	61,5	70,61	70,89	69,18	65,22	73,48	92,757	95,016	268,59	264,46	287,27	109,35	112,01	120,68	145,09	140,65	134,94	143,87	150,46
Val du Levant R.	61,57	69,77	62,83	26,83	20,75	0	31,374	33,494	44,484	53,594	53,874	52,164	48,204	56,464	75,741	78	281,85	277,72	300,53	92,334	94,994	103,66	128,07	123,63	117,92	126,85	133,44
Anse de la Canicule R.	89,21	97,41	90,47	54,47	48,39	31,374	0	2,847	13,837	22,947	23,227	21,517	17,557	25,817	45,094	47,353	309,49	305,36	328,17	61,687	64,347	73,017	97,427	92,987	87,277	96,207	102,8
Crête des Laves R.	91,33	99,53	92,59	56,59	50,51	33,494	2,847	0	11,242	20,352	20,632	18,922	14,962	23,222	42,499	44,758	311,61	307,48	330,29	59,092	61,752	70,422	94,832	90,392	84,682	93,612	100,2
Halage des Swains R.	102,32	110,52	103,58	67,58	61,5	44,484	13,837	11,242	0	11,671	14,99	13,188	9,58	28,59	47,91	50,22	322,6	318,47	341,28	64,53	67,284	75,92	100,62	96,16	90,89	99,16	106,11
Northern arm of Anse aux Ecueils Brook	111,43	119,63	112,69	76,69	70,61	53,594	22,947	20,352	11,671	0	16,05	14,3	10,3	30,32	49,42	51,78	331,71	327,58	350,39	65,75	68,61	77,15	101,84	97,39	92,11	100,38	107,34
La Clarée Basin: Hermance Lake & Galets R.	111,71	119,91	112,97	76,97	70,89	53,874	23,227	20,632	14,99	16,05	0	2,57	6,41	27,79	47,15	49,83	331,99	327,86	350,67	63,56	66,31	74,95	99,64	95,193	89,92	98,19	105,14
Olsen R.	110	118,2	111,26	75,26	69,18	52,164	21,517	18,922	13,188	14,3	2,57	0	4,44	25,74	45,14	47,45	330,28	326,15	348,96	61,56	64,32	72,96	97,65	93,2	87,92	96,2	103,15
Vallée du Radioleine R.	106,04	114,24	107,3	71,3	65,22	48,204	17,557	14,962	9,58	10,3	6,41	4,44	0	21,43	40,45	43,28	326,32	322,19	345	57,3	60,05	68,69	93,39	88,93	83,66	91,93	98,88
Val des Skuas R.	114,3	122,5	115,56	79,56	73,48	56,464	25,817	23,222	28,59	30,32	27,79	25,74	21,43	0	20,19	22,65	334,58	330,45	353,26	36,61	39,34	47,98	72,67	68,17	62,86	71,17	78,17
Doigt de Sainte-Anne R.	133,58	141,78	134,84	98,837	92,757	75,741	45,094	42,499	47,91	49,42	47,15	45,14	40,45	20,19	0	2,44	353,85	349,72	372,54	16,91	19,55	28,36	53,05	48,6	43,27	51,6	58,55
Plaine de Dante R.	135,84	144,04	137,1	101,1	95,016	78	47,353	44,758	50,22	51,78	49,83	47,45	43,28	22,65	2,44	0	356,11	351,98	374,8	14,71	17,54	26,35	51,05	46,59	41,24	49,59	56,54
Mont Ventoux R.	243,13	251,13	244,09	256,54	268,59	281,85	309,49	311,61	322,6	331,71	331,99	330,28	326,32	334,58	353,85	356,11	0	9,14	46,01	370,45	373,11	381,78	406,19	401,75	396,04	404,97	411,56
Mont Sinaï R.	239	247	239,96	252,41	264,46	277,72	305,36	307,48	318,47	327,58	327,86	326,15	322,19	330,45	349,72	351,98	9,14	0	39,32	366,32	368,98	377,65	402,06	397,62	391,91	400,84	407,43
Bastille R.	261,81	269,81	262,77	275,22	287,27	300,53	328,17	330,29	341,28	350,39	350,67	348,96	345	353,26	372,54	374,8	46,01	39,32	0	389,13	391,79	400,46	424,87	420,43	414,72	423,65	430,24
Lac Mariot outlet	150,17	158,37	151,43	115,43	109,35	92,334	61,687	59,092	64,53	65,75	63,56	61,56	57,3	36,61	16,91	14,71	370,45	366,32	389,13	0	28,01	36,82	61,52	57,06	51,74	60,06	67,01
Plaine d'Ampère R.	152,83	161,03	154,09	118,09	112,01	94,994	64,347	61,752	67,284	68,61	66,31	64,32	60,05	39,34	19,55	17,54	373,11	368,98	391,79	28,01	0	9,6	34,9	30,54	25,31	33,66	39,95
Portes Noires R.	161,5	169,7	162,76	126,76	120,68	103,66	73,017	70,422	75,92	77,15	74,95	72,96	68,69	47,98	28,36	26,35	381,78	377,65	400,46	36,82	9,6	0	26,923	22,563	17,333	25,683	31,973
Contacts R.	185,91	194,11	187,17	151,17	145,09	128,07	97,427	94,832	100,62	101,84	99,64	97,65	93,39	72,67	53,05	51,05	406,19	402,06	424,87	61,52	34,9	26,923	0	5,04	14,58	22,81	29,867
Mouche R.	181,47	189,67	182,73	146,73	140,65	123,63	92,987	90,392	96,16	97,39	95,193	93,2	88,93	68,17	48,6	46,59	401,75	397,62	420,43	57,06	30,54	22,563	5,04	0	9,98	18,21	25,26
Larmor R.	175,76	183,96	177,02	141,02	134,94	117,92	87,277	84,682	90,89	92,11	89,92	87,92	83,66	62,86	43,27	41,24	396,04	391,91	414,72	51,74	25,31	17,333	14,58	9,98	0	8,8	15,86
Plage Jaune R.	184,69	192,89	185,95	149,95	143,87	126,85	96,207	93,612	99,16	100,38	98,19	96,2	91,93	71,17	51,6	49,59	404,97	400,84	423,65	60,06	33,66	25,683	22,81	18,21	8,8	0	7,43
Sables R.	191,28	199,48	192,54	156,54	150,46	133,44	102,8	100,2	106,11	107,34	105,14	103,15	98,88	78,17	58,55	56,54	411,56	407,43	430,24	67,01	39,95	31,973	29,867	25,26	15,86	7,43	0

Labonne et al. 2013, Appendix 2B: Distance matrix between hydrographic systems calculated with a 1 km buffer

Patch names	Entrelacs R.	Baie Chanzy	Déversoir du Lac de la Croix du Sud	Outlet of Lac Euphrosine	Ballon R.	Sans Nom R.	S. Brook Ile aux Skuas	Brook 1 Ile du Port	Brook 2 Ile du Port	Brook 3 Ile du Port	Brook 4 Ile du Port	Val Froide R.	Val Travers R.	Brook N. of Port-Couvreux	Brook of Port-Couvreux cabin	Brook W end of Havre du Beau Temps	Anse Sablonneuse Brook	Port Kirk R.	Val de l'Ouest R.	Studer R.	Doute R.	Charbon R.	Sérail R.	Chasseurs R.	Nord R.	Pépins R.	Cataractes R.	Hautes Mares South Brook (Gorfous 1)	Hautes Mares North Brook (Gorfous 2)	Pointe des Cabanes Brook (Gorfous 4)	Cap Rouge Brook (Gorfous 5)	N. Cap Rouge Brook (Gorfous 6)	Cap de Rohan R.	Lac Marville Outlet	Manchots R.
Entrelacs R.	0	24,83	25,03	32,37	31,75	31,17	30,95	32,15	33,56	35,25	36,031	39,23	34,08	44,32	45,13	55,58	46,56	47,73	47,81	45,94	50	50,12	53,96	58,66	71,72	73,01	72,93	73,04	73,13	72,89	75,78	76,43	84,48	102,18	111,15
Baie Chanzy	24,83	0	10,44	20,31	19,46	19,01	19	21,53	23,28	24,77	25,53	27,42	22,23	33,42	34,47	44,85	35,96	37,02	37,1	38,27	43,22	43,43	47,42	51,97	65,158	66,572	66,492	66,602	66,692	66,452	69,342	69,992	78,042	95,742	104,71
Déversoir du Lac de la Croix du Sud	25,03	10,44	0	15,923	15,073	14,623	14,613	17,297	19,047	20,537	21,297	23,033	17,843	28,92	29,96	40,346	31,45	32,58	32,73	34,05	38,9	39,2	43,197	47,75	60,93	62,227	62,147	62,257	62,347	62,107	64,997	65,647	73,697	91,397	100,37
Outlet of Lac Euphrosine	32,37	20,31	15,923	0	1,59	3,04	13,12	15,804	17,554	19,044	19,804	20,279	15,089	27,14	28,19	38,57	29,68	30,82	31,01	32,63	37,83	38,1	42,13	46,66	59,84	61,136	61,056	61,166	61,256	61,016	63,906	64,556	72,606	90,306	99,276
Ballon R.	31,75	19,46	15,073	1,59	0	2,178	12,258	14,942	16,692	18,182	18,942	19,417	14,227	26,278	27,328	37,708	28,818	29,958	30,148	31,768	36,968	37,238	41,268	45,798	58,978	60,274	60,194	60,304	60,394	60,154	63,044	63,694	71,744	89,444	98,414
Sans Nom R.	31,17	19,01	14,623	3,04	2,178	0	11,798	14,482	16,232	17,722	18,482	18,957	13,767	25,818	26,868	37,248	28,358	29,498	29,688	31,308	36,508	36,778	40,808	45,338	58,518	59,814	59,734	59,844	59,934	59,694	62,584	63,234	71,284	88,984	97,954
S. Brook Ile aux Skuas	30,95	19	14,613	13,12	12,258	11,798	0	9,182	9,72	10,82	11,68	13,14	7,58	16,6	17,53	28,18	19,37	20,6	20,55	22,85	28,33	28,51	32,63	37,34	50,84	51,916	51,836	51,946	52,036	51,796	54,686	55,336	63,386	81,086	90,056
Brook 1 Ile du Port	32,15	21,53	17,297	15,804	14,942	14,482	9,182	0	3,52	4,98	5,81	20,72	15,16	15,48	16,23	26,82	17,8	18,34	18,17	18,7	23,63	23,91	27,67	32,33	45,47	46,899	46,819	46,929	47,019	46,779	49,669	50,319	58,369	76,069	85,039
Brook 2 Ile du Port	33,56	23,28	19,047	17,554	16,692	16,232	9,72	3,52	0	3,67	4,48	21,92	16,48	14,85	15,6	26,19	17,18	17,72	17,54	17,56	22,6	22,77	26,46	31,12	44,263	45,533	45,453	45,563	45,653	45,413	48,303	48,953	57,003	74,703	83,673
Brook 3 Ile du Port	35,25	24,77	20,537	19,044	18,182	17,722	10,82	4,98	3,67	0	1,89	23,28	17,84	15,24	15,98	26,57	17,55	18,06	16,63	16,34	21,1	21,3	25,03	29,66	42,82	44,259	44,179	44,289	44,379	44,139	47,029	47,679	55,729	73,429	82,399
Brook 4 Ile du Port	36,031	25,53	21,297	19,804	18,942	18,482	11,68	5,81	4,48	1,89	0	24,05	18,65	15,94	16,68	27,27	18,26	18,76	17,33	16,94	21,47	21,63	25,32	29,94	43,11	44,476	44,396	44,506	44,596	44,356	47,246	47,896	55,946	73,646	82,616
Val Froide R.	39,23	27,42	23,033	20,279	19,417	18,957	13,14	20,72	21,92	23,28	24,05	0	6,37	29,172	30,577	41,081	32,001	33,25	33,2	35,53	41,02	41,2	45,32	50,03	63,51	64,595	64,515	64,625	64,715	64,475	67,365	68,015	76,065	93,765	102,74
Val Travers R.	34,08	22,23	17,843	15,089	14,227	13,767	7,58	15,16	16,48	17,84	18,65	6,37	0	23,629	25,034	35,538	26,458	27,63	27,6	30,1	35,49	35,58	39,79	44,51	57,99	58,909	58,829	58,939	59,029	58,789	61,679	62,329	70,379	88,079	97,049
Brook N. of Port-Couvreux	44,32	33,42	28,92	27,14	26,278	25,818	16,6	15,48	14,85	15,24	15,94	29,172	23,629	0	3,44	14,27	5,26	12,06	12,23	16,68	22,17	22,42	26,85	31,4	44,54	46,007	45,927	46,037	46,127	45,887	48,777	49,427	57,477	75,177	84,147
Brook of Port-Couvreux cabin	45,13	34,47	29,96	28,19	27,328	26,868	17,53	16,23	15,6	15,98	16,68	30,577	25,034	3,44	0	12,43	3,55	12,994	13,164	17,614	23,104	23,354	27,784	32,334	45,474	46,941	46,861	46,971	47,061	46,821	49,711	50,361	58,411	76,111	85,081
Brook W end of Havre du Beau Temps	55,58	44,85	40,346	38,57	37,708	37,248	28,18	26,82	26,19	26,57	27,27	41,081	35,538	14,27	12,43	0	11,76	22,637	22,807	27,257	32,747	32,997	37,427	41,977	55,117	56,584	56,504	56,614	56,704	56,464	59,354	60,004	68,054	85,754	94,724
Anse Sablonneuse Brook	46,56	35,96	31,45	29,68	28,818	28,358	19,37	17,8	17,18	17,55	18,26	32,001	26,458	5,26	3,55	11,76	0	13,399	13,569	18,019	23,509	23,759	28,189	32,739	45,879	47,346	47,266	47,376	47,466	47,226	50,116	50,766	58,816	76,516	85,486
Port Kirk R.	47,73	37,02	32,58	30,82	29,958	29,498	20,6	18,34	17,72	18,06	18,76	33,25	27,63	12,06	12,994	22,637	13,399	0	6,96	13,074	18,564	18,814	23,244	27,794	40,934	42,401	42,321	42,431	42,521	42,281	45,171	45,821	53,871	71,571	80,541
Val de l'Ouest R.	47,81	37,1	32,73	31,01	30,148	29,688	20,55	18,17	17,54	16,63	17,33	33,2	27,6	12,23	13,164	22,807	13,569	6,96	0	8,919	14,409	14,659	19,089	23,639	36,779	38,246	38,166	38,276	38,366	38,126	41,016	41,666	49,716	67,416	76,386
Studer R.	45,94	38,27	34,05	32,63	31,768	31,308	22,85	18,7	17,56	16,34	16,94	35,53	30,1	16,68	17,614	27,257	18,019	13,074	8,919	0	7,415	7,645	11,905	16,705	29,666	31,133	31,053	31,163	31,253	31,013	33,903	34,553	42,603	60,303	69,273
Doute R.	50	43,22	38,9	37,83	36,968	36,508	28,33	23,63	22,6	21,1	21,47	41,02	35,49	22,17	23,104	32,747	23,509	18,564	14,409	7,415	0	2,761	7,021	11,821	25,621	27,088	27,008	27,118	27,208	26,968	29,858	30,508	38,558	56,258	65,228
Charbon R.	50,12	43,43	39,2	38,1	37,238	36,778	28,51	23,91	22,77	21,3	21,63	41,2	35,58	22,42	23,354	32,997	23,759	18,814	14,659	7,645	2,761	0	7,374	12,174	25,539	27,006	26,926	27,036	27,126	26,886	29,776	30,426	38,476	56,176	65,146
Sérail R.	53,96	47,42	43,197	42,13	41,268	40,808	32,63	27,67	26,46	25,03	25,32	45,32	39,79	26,85	27,784	37,427	28,189	23,244	19,089	11,905	7,021	7,374	0	6,418	19,751	21,218	21,138	21,248	21,338	21,098	23,988	24,638	32,688	50,388	59,358
Chasseurs R.	58,66	51,97	47,75	46,66	45,798	45,338	37,34	32,33	31,12	29,66	29,94	50,03	44,51	31,4	32,334	41,977	32,739	27,794	23,639	16,705	11,821	12,174	6,418	0	15,377	16,844	16,764	16,874	16,964	16,724	19,614	20,264	28,314	46,014	54,984
Nord R.	71,72	65,158	60,93	59,84	58,978	58,518	50,84	45,47	44,263	42,82	43,11	63,51	57,99	44,54	45,474	55,117	45,879	40,934	36,779	29,666	25,621	25,539	19,751	15,377	0	7,82	8,01	8,57	8,78	10,28	14,82	15,36	23,527	41,227	50,197
Pépins R.	73,01	66,572	62,227	61,136	60,274	59,814	51,916	46,899	45,533	44,259	44,476	64,595	58,909	46,007	46,941	56,584	47,346	42,401	38,246	31,133	27,088	27,006	21,218	16,844	7,82	0	3,3	4,37	4,73	7,22	12,05	12,59	20,755	38,455	47,425
Cataractes R.	72,93	66,492	62,147	61,056	60,194	59,734	51,836	46,819	45,453	44,179	44,396	64,515	58,829	45,927	46,861	56,504	47,266	42,321	38,166	31,053	27,008	26,926	21,138	16,764	8,01	3,3	0	1,89	2,05	4,87	9,95	10,28	18,406	36,106	45,076
Hautes Mares South Brook (Gorfous 1)	73,04	66,602	62,257	61,166	60,304	59,844	51,946	46,929	45,563	44,289	44,506	64,625	58,939	46,037	46,971	56,614	47,376	42,431	38,276	31,163	27,118	27,036	21,248	16,874	8,57	4,37	1,89	0	0,44	3,8	8,41	8,88	17,115	34,815	43,785
Hautes Mares North Brook (Gorfous 2)	73,13	66,692	62,347	61,256	60,394	59,934	52,036	47,019	45,653	44,379	44,596	64,715	59,029	46,127	47,061	56,704	47,466	42,521	38,366	31,253	27,208	27,126	21,338	16,964	8,78	4,73	2,05	0,44	0	3,57	8,44	8,64	16,726	34,426	43,396
Pointe des Cabanes Brook (Gorfous 4)	72,89	66,452	62,107	61,016	60,154	59,694	51,796	46,779	45,413	44,139	44,356	64,475	58,789	45,887	46,821	56,464	47,226	42,281	38,126	31,013	26,968	26,886	21,098	16,724	10,28	7,22	4,87	3,8	3,57	0	5,04	5,43	13,532	31,232	40,202
Cap Rouge Brook (Gorfous 5)	75,78	69,342	64,997	63,906	63,044	62,584	54,686	49,669	48,303	47,029	47,246	67,365	61,679	48,777	49,711	59,354	50,116	45,171	41,016	33,903	29,858	29,776	23,988	19,614	14,82	12,05	9,95	8,41	8,44	5,04	0	0,96	9,389	27,089	36,059
N. Cap Rouge Brook (Gorfous 6)	76,43	69,992	65,647	64,556	63,694	63,234	55,336	50,319	48,953	47,679	47,896	68,015	62,329	49,427	50,361	60,004	50,766	45,821	41,666	34,553	30,508	30,426	24,638	20,264	15,36	12,59	10,28	8,88	8,64	5,43	0,96	0	8,52	26,22	35,19
Cap de Rohan R.	84,48	78,042	73,697	72,606	71,744	71,284	63,386	58,369	57,003	55,729	55,946	76,065	70,379	57,477	58,411	68,054	58,816	53,871	49,716	42,603	38,558	38,476	32,688	28,314	23,527	20,755	18,406	17,115	16,726	13,532	9,389	8,52	0	17,868	26,838
Lac Marville Outlet	102,18	95,742	91,397	90,306	89,444	88,984	81,086	76,069	74,703	73,429	73,646	93,765	88,079	75,177	76,111	85,754	76,516	71,571	67,416	60,303	56,258	56,176	50,388	46,014	41,227	38,455	36,106	34,815	34,426	31,232	27,089	26,22	17,868	0	9,72
Manchots R.	111,15	104,71	100,37	99,276	98,414	97,954	90,056	85,039	83,673	82,399	82,616	102,74	97,049	84,147	85,081	94,724	85,486	80,541	76,386	69,273	65,228	65,146	59,358	54,984	50,197	47,425	45,076	43,785	43,396	40,202	36,059	35,19	26,838	9,72	0
Calcédoines R.	123,64	117,21	112,86	111,77	110,91	110,45	102,55	97,533	96,167	94,893	95,11	115,23	109,54	96,641	97,575	107,22	97,98	93,035	88,88	81,767	77,722	77,64	71,852	67,478	62,691	59,919	57,57	56,279	55,89	52,696	48,553	47,684	39,332	22,214	12,395
Bungay R.	127,83	121,4	117,05	115,96	115,1	114,64	106,74	101,72	100,36	99,083	99,3	119,42	113,73	100,83	101,77	111,41	102,17	97,225	93,07	85,957	81,912	81,83	76,042	71,668	66,881	64,109	61,76	60,469	60,08	56,886	52,743	51,874	43,522	26,404	16,585
Albatros R.	137,33	130,89	126,55	125,46	124,59	124,13	116,24	111,22	109,85	108,58	108,8	128,92	123,23	110,33	111,26	120,9	111,67	106,72	102,57	95,453	91,408	91,326	85,538	81,164	76,377	73,605	71,256	69,965	69,576	66,382	62,239	61,37	53,018	35,9	26,081
Norvégienne R.	138,96	132,52	128,18	127,09	126,22	125,76	117,87	112,85	111,48	110,21	110,43	130,55	124,86	111,96	112,89	122,53	113,3	108,35	104,2	97,083	93,038	92,956	87,168	82,794	78,007	75,235	72,886	71,595	71,206	68,012	63,869	63	54,648	37,53	27,711
Château R.	139,96	133,52	129,18	128,09	127,22	126,76	118,87	113,85	112,48	111,21	111,43	131,55	125,86	112,96	113,89	123,53	114,3	109,35	105,2	98,083	94,038	93,956	88,168	83,794	79,007	76,235	73,886	72,595	72,206	69,012	64,869	64	55,648	38,53	28,711
PAF Ferme R.	159,05	152,61	148,27	147,18	146,31	145,85	137,96	132,94	131,57	130,3	130,52	150,64	144,95	132,05	132,98	142,62	133,39	128,44	124,29	117,17	113,13	113,05	107,26	102,88	98,097	95,325	92,976	91,685	91,296	88,102	83,959	83,09	74,738	57,62	47,801
Borgne R.	160,99	154,55	150,21	149,12	148,25	147,79	139,9	134,88	133,51	132,24	132,46	152,58	146,89	133,99	134,92	144,56	135,33	130,38	126,23	119,11	115,07	114,99	109,2	104,82	100,04	97,265	94,916	93,625	93,236	90,042	85,899	85,03	76,678	59,56	49,741
Sud R.	162,91	156,47	152,13	151,04	150,17	149,71	141,82	136,8	135,43	134,16	134,38	154,5	148,81	135,91	136,84	146,48	137,25	132,3	128,15	121,03	116,99	116,91	111,12	106,74	101,96	99,185	96,836	95,545	95,156	91,962	87,819	86,95	78,598	61,48	51,661
Pointe de l'épave Brook	164,13	157,69	153,35	152,26	151,39	150,93	143,04	138,02	136,65	135,38	135,6	155,72	150,03	137,13	138,06	147,7	138,47	133,52	129,37	122,25	118,21	118,13	112,34	107,96	103,18	100,41	98,056	96,765	96,376	93,182	89,039	88,17	79,818	62,7	52,881
Américains R.	165,44	159	154,66	153,57	152,7	152,24	144,35	139,33	137,96	136,69	136,91	157,03	151,34	138,44	139,37	149,01	139,78	134,83	130,68	123,56	119,52	119,44	113,65	109,27	104,49	101,72	99,366	98,075	97,686	94,492	90,349	89,48	81,128	64,01	54,191
3 Lacs R.	169,8	163,36	159,02	157,93	157,06	156,6	148,71	143,69	142,32	141,05	141,27	161,39	155,7	142,8	143,73	153,37	144,14	139,19	135,04	127,92	123,88	123,8	118,01	113,63	108,85	106,08	103,73	102,44	102,05	98,852	94,709	93,84	85,488	68,37	58,551
Val d'Auge R.	177,42	170,98	166,64	165,55	164,68	164,22	156,33	151,31	149,94	148,67	148,89	169,01	163,32	150,42	151,35	160,99	151,76	146,81	142,66	135,54	131,5	131,42	125,63	121,25	116,47	113,7	111,35	110,06	109,67	106,47	102,33	101,46	93,108	75,99	66,171
Anse de St Malo R.	184,99	178,55	174,21	173,12	172,25	171,79	163,9	158,88	157,51	156,24	156,46	176,58	170,89	157,99	158,92	168,56	159,33	154,38	150,23	143,11	139,07	138,99	133,2	128,82	124,04	121,27	118,92	117,63	117,24	114,04	109,9	109,03	100,68	83,56	73,741
Basin downstream of Korrigans Lake	178,48	172,04	167,7	166,61	165,74	165,28	157,39	152,37	151	149,73	149,95	170,07	164,38	151,48	152,41	162,05	152,82	147,87	143,72	136,6	132,56	132,48	126,69	122,31	117,53	114,76	112,41	111,12	110,73	107,53	103,39	102,52	94,168	77,05	67,231
Baie de l'Observatoire R.	177,31	170,87	166,53	165,44	164,57	164,11	156,22	151,2	149,83	148,56	148,78	168,9	163,21	150,31	151,24	160,88	151,65	146,7	142,55	135,43	131,39	131,31	125,52	121,14	116,36	113,59	111,24	109,95	109,56	106,36	102,22	101,35	92,998	75,88	66,061
SW Brook on Bras Jules Laboureur	184,76	178,32	173,98	172,89	172,02	171,56	163,67	158,65	157,28	156,01	156,23	176,35	170,66	157,76	158,69	168,33	159,1	154,15	150	142,88	138,84	138,76	132,97	128,59	123,81	121,04	118,69	117,4	117,01	113,81	109,67	108,8	100,45	83,33	73,511
Grisanche R.	201,86	195,42	191,08	189,99	189,12	188,66	180,77	175,75	174,38	173,11	173,33	193,45	187,76	174,86	175,79	185,43	176,2	171,25	167,1	159,98	155,94	155,86	150,07	145,69	140,91	138,14	135,79	134,5	134,11	130,91	126,77	125,9	117,55	100,43	90,611
Lac des Fougères Outlet	197,71	191,27	186,93	185,84	184,97	184,51	176,62	171,6	170,23	168,96	169,18	189,3	183,61	170,71	171,64	181,28	172,05	167,1	162,95	155,83	151,79	151,71	145,92	141,54	136,76	133,99	131,64	130,35	129,96	126,76	122,62	121,75	113,4	96,28	86,461
Lac d'Armor Basin	189,44	183	178,66	177,57	176,7	176,24	168,35	163,33	161,96	160,69	160,91	181,03	175,34	162,44	163,37	173,01	163,78	158,83	154,68	147,56	143,52	143,44	137,65	133,27	128,49	125,72	123,37	122,08	121,69	118,49	114,35	113,48	105,13	88,01	78,191
Anse J. Bourcart Brook	185,85	179,41	175,07	173,98	173,11	172,65	164,76	159,74	158,37	157,1	157,32	177,44	171,75	158,85	159,78	169,42	160,19	155,24	151,09	143,97	139,93	139,85	134,06	129,68	124,9	122,13	119,78	118,49	118,1	114,9	110,76	109,89	101,54	84,42	74,601
Ravin du Charbon R.	188,59	182,15	177,81	176,72	175,85	175,39	167,5	162,48	161,11	159,84	160,06	180,18	174,49	161,59	162,52	172,16	162,93	157,98	153,83	146,71	142,67	142,59	136,8	132,42	127,64	124,87	122,52	121,23	120,84	117,64	113,5	112,63	104,28	87,16	77,341
Vallée des Neiges Basin	183,84	177,4	173,06	171,97	171,1	170,64	162,75	157,73	156,36	155,09	155,31	175,43	169,74	156,84	157,77	167,41	158,18	153,23	149,08	141,96	137,92	137,84	132,05	127,67	122,89	120,12	117,77	116,48	116,09	112,89	108,75	107,88	99,528	82,41	72,591
Acaena R.	178,99	172,55	168,21	167,12	166,25	165,79	157,9	152,88	151,51	150,24	150,46	170,58	164,89	151,99	152,92	162,56	153,33	148,38	144,23	137,11	133,07	132,99	127,2	122,82	118,04	115,27	112,92	111,63	111,24	108,04	103,9	103,03	94,678	77,56	67,741
Vallée de la Planchette R.	176,27	169,83	165,49	164,4	163,53	163,07	155,18	150,16	148,79	147,52	147,74	167,86	162,17	149,27	150,2	159,84	150,61	145,66	141,51	134,39	130,35	130,27	124,48	120,1	115,32	112,55	110,2	108,91	108,52	105,32	101,18	100,31	91,958	74,84	65,021
Val Raide Brook	184,23	177,79	173,45	172,36	171,49	171,03	163,14	158,12	156,75	155,48	155,7	175,82	170,13	157,23	158,16	167,8	158,57	153,62	149,47	142,35	138,31	138,23	132,44	128,06	123,28	120,51	118,16	116,87	116,48	113,28	109,14	108,27	99,918	82,8	72,981
Mouettes R.	177,32	170,88	166,54	165,45	164,58	164,12	156,23	151,21	149,84	148,57	148,79	168,91	163,22	150,32	151,25	160,89	151,66	146,71	142,56	135,44	131,4	131,32	125,53	121,15	116,37	113,6	111,25	109,96	109,57	106,37	102,23	101,36	93,008	75,89	66,071
Macaronis R.	159,8	153,36	149,02	147,93	147,06	146,6	138,71	133,69	132,32	131,05	131,27	151,39	145,7	132,8	133,73	143,37	134,14	129,19	125,04	117,92	113,88	113,8	108,01	103,63	98,847	96,075	93,726	92,435	92,046	88,852	84,709	83,84	75,488	58,37	48,551
Vallée Phonolite Basin	171,96	165,52	161,18	160,09	159,22	158,76	150,87	145,85	144,48	143,21	143,43	163,55	157,86	144,96	145,89	155,53	146,3	141,35	137,2	130,08	126,04	125,96	120,17	115,79	111,01	108,24	105,89	104,6	104,21	101,01	96,869	96	87,648	70,53	60,711
Val du Levant R.	179,68	173,24	168,9	167,81	166,94	166,48	158,59	153,57	152,2	150,93	151,15	171,27	165,58	152,68	153,61	163,25	154,02	149,07	144,92	137,8	133,76	133,68	127,89	123,51	118,73	115,96	113,61	112,32	111,93	108,73	104,59	103,72	95,368	78,25	68,431
Anse de la Canicule R.	206,42	199,98	195,64	194,55	193,68	193,22	185,33	180,31	178,94	177,67	177,89	198,01	192,32	179,42	180,35	189,99	180,76	175,81	171,66	164,54	160,5	160,42	154,63	150,25	145,47	142,7	140,35	139,06	138,67	135,47	131,33	130,46	122,11	104,99	95,171
Crête des Laves R.	208,54	202,1	197,76	196,67	195,8	195,34	187,45	182,43	181,06	179,79	180,01	200,13	194,44	181,54	182,47	192,11	182,88	177,93	173,78	166,66	162,62	162,54	156,75	152,37	147,59	144,82	142,47	141,18	140,79	137,59	133,45	132,58	124,23	107,11	97,291
Halage des Swains R.	219,53	213,09	208,75	207,66	206,79	206,33	198,44	193,42	192,05	190,78	191	211,12	205,43	192,53	193,46	203,1	193,87	188,92	184,77	177,65	173,61	173,53	167,74	163,36	158,58	155,81	153,46	152,17	151,78	148,58	144,44	143,57	135,22	118,1	108,28
Northern arm of Anse aux Ecueils Brook	228,65	222,22	217,87	216,78	215,92	215,46	207,56	202,54	201,18	199,9	200,12	220,24	214,55	201,65	202,58	212,23	202,99	198,04	193,89	186,78	182,73	182,65	176,86	172,49	167,7	164,93	162,58	161,29	160,9	157,71	153,56	152,69	144,34	127,22	117,4
La Clarée Basin: Hermance Lake & Galets R.	227,91	221,47	217,13	216,04	215,17	214,71	206,82	201,8	200,43	199,16	199,38	219,5	213,81	200,91	201,84	211,48	202,25	197,3	193,15	186,03	181,99	181,91	176,12	171,74	166,96	164,19	161,84	160,55	160,16	156,96	152,82	151,95	143,6	126,48	116,66
Olsen R.	226,01	219,57	215,23	214,14	213,27	212,81	204,92	199,9	198,53	197,26	197,48	217,6	211,91	199,01	199,94	209,58	200,35	195,4	191,25	184,13	180,09	180,01	174,22	169,84	165,06	162,29	159,94	158,65	158,26	155,06	150,92	150,05	141,7	124,58	114,76
Vallée du Radioleine R.	221,71	215,27	210,93	209,84	208,97	208,51	200,62	195,6	194,23	192,96	193,18	213,3	207,61	194,71	195,64	205,28	196,05	191,1	186,95	179,83	175,79	175,71	169,92	165,54	160,76	157,99	155,64	154,35	153,96	150,76	146,62	145,75	137,4	120,28	110,46
Val des Skuas R.	219,82	213,38	209,04	207,95	207,08	206,62	198,73	193,71	192,34	191,07	191,29	211,41	205,72	192,82	193,75	203,39	194,16	189,21	185,06	177,94	173,9	173,82	168,03	163,65	158,87	156,1	153,75	152,46	152,07	148,87	144,73	143,86	135,51	118,39	108,57
Doigt de Sainte-Anne R.	239,09	232,65	228,31	227,22	226,35	225,89	218	212,98	211,61	210,34	210,56	230,68	224,99	212,09	213,02	222,66	213,43	208,48	204,33	197,21	193,17	193,09	187,3	182,92	178,14	175,37	173,02	171,73	171,34	168,14	164	163,13	154,78	137,66	127,84
Plaine de Dante R.	241,35	234,91	230,57	229,48	228,61	228,15	220,26	215,24	213,87	212,6	212,82	232,94	227,25	214,35	215,28	224,92	215,69	210,74	206,59	199,47	195,43	195,35	189,56	185,18	180,4	177,63	175,28	173,99	173,6	170,4	166,26	165,39	157,04	139,92	130,1
Mont Ventoux R.	2,67	22,716	22,916	30,256	29,636	29,056	28,836	29,849	31,259	32,949	33,73	36,929	31,779	42,019	42,829	53,279	44,259	45,429	45,509	43,639	47,699	47,819	51,659	56,359	69,419	70,709	70,629	70,739	70,829	70,589	73,479	74,129	82,179	99,879	108,85
Mont Sinaï R.	8,93	18,586	18,786	26,126	25,506	24,926	24,706	25,868	27,278	28,968	29,749	32,948	27,798	38,038	38,848	49,298	40,278	41,448	41,528	39,658	43,718	43,838	47,678	52,378	65,438	66,728	66,648	66,758	66,848	66,608	69,498	70,148	78,198	95,898	104,87
Bastille R.	44,56	38,32	36,424	40,51	39,28	38,881	38,98	28,88	27,35	24,78	24,68	47,62	41,82	36,89	36,99	48,18	38,75	37,29	35,25	31,85	36,08	36,09	40	44,18	57,48	59,05	58,44	58,67	58,66	58,64	61,366	62,295	70,127	87,995	96,965
Lac Mariot outlet	255,57	249,13	244,79	243,7	242,83	242,37	234,48	229,46	228,09	226,82	227,04	247,16	241,47	228,57	229,5	239,14	229,91	224,96	220,81	213,69	209,65	209,57	203,78	199,4	194,62	191,85	189,5	188,21	187,82	184,62	180,48	179,61	171,26	154,14	144,32
Plaine d'Ampère R.	256,84	250,4	246,06	244,97	244,1	243,64	235,75	230,73	229,36	228,09	228,31	248,43	242,74	229,84	230,77	240,41	231,18	226,23	222,08	214,96	210,92	210,84	205,05	200,67	195,89	193,12	190,77	189,48	189,09	185,89	181,75	180,88	172,53	155,41	145,59
Portes Noires R.	264,62	258,18	253,84	252,75	251,88	251,42	243,53	238,51	237,14	235,87	236,09	256,21	250,52	237,62	238,55	248,19	238,96	234,01	229,86	222,74	218,7	218,62	212,83	208,45	203,67	200,9	198,55	197,26	196,87	193,67	189,53	188,66	180,31	163,19	153,37
Contacts R.	265,85	259,41	255,07	253,98	253,11	252,65	244,76	239,74	238,37	237,1	237,32	257,44	251,75	238,85	239,78	249,42	240,19	235,24	231,09	223,97	219,93	219,85	214,06	209,68	204,9	202,13	199,78	198,49	198,1	194,9	190,76	189,89	181,54	164,42	154,6
Mouche R.	261,31	254,87	250,53	249,44	248,57	248,11	240,22	235,2	233,83	232,56	232,78	252,9	247,21	234,31	235,24	244,88	235,65	230,7	226,55	219,43	215,39	215,31	209,52	205,14	200,36	197,59	195,24	193,95	193,56	190,36	186,22	185,35	177	159,88	150,06
Larmor R.	255,32	248,88	244,54	243,45	242,58	242,12	234,23	229,21	227,84	226,57	226,79	246,91	241,22	228,32	229,25	238,89	229,66	224,71	220,56	213,44	209,4	209,32	203,53	199,15	194,37	191,6	189,25	187,96	187,57	184,37	180,23	179,36	171,01	153,89	144,07
Plage Jaune R.	261,54	255,1	250,76	249,67	248,8	248,34	240,45	235,43	234,06	232,79	233,01	253,13	247,44	234,54	235,47	245,11	235,88	230,93	226,78	219,66	215,62	215,54	209,75	205,37	200,59	197,82	195,47	194,18	193,79	190,59	186,45	185,58	177,23	160,11	150,29
Sables R.	268,39	261,95	257,61	256,52	255,65	255,19	247,3	242,28	240,91	239,64	239,86	259,98	254,29	241,39	242,32	251,96	242,73	237,78	233,63	226,51	222,47	222,39	216,6	212,22	207,44	204,67	202,32	201,03	200,64	197,44	193,3	192,43	184,08	166,96	157,14

Patch names	Calcédoines R.	Bungay R.	Albatros R.	Norvégienne R.	Château R.	PAF Ferme R.	Borgne R.	Sud R.	Pointe de l'épave Brook	Américains R.	3 Lacs R.	Val d'Auge R.	Anse de St Malo R.	Basin downstream of Korrigans Lake	Baie de l'Observatoire R.	SW Brook on Bras Jules Laboureur	Grisanche R.	Lac des Fougères Outlet	Lac d'Armor Basin	Anse J. Bourcart Brook	Ravin du Charbon R.	Vallée des Neiges Basin	Acaena R.	Vallée de la Planchette R.	Val Raide Brook	Mouettes R.
Entrelacs R.	123,64	127,83	137,33	138,96	139,96	159,05	160,99	162,91	164,13	165,44	169,8	177,42	184,99	178,48	177,31	184,76	201,86	197,71	189,44	185,85	188,59	183,84	178,99	176,27	184,23	177,32
Baie Chanzy	117,21	121,4	130,89	132,52	133,52	152,61	154,55	156,47	157,69	159	163,36	170,98	178,55	172,04	170,87	178,32	195,42	191,27	183	179,41	182,15	177,4	172,55	169,83	177,79	170,88
Déversoir du Lac de la Croix du Sud	112,86	117,05	126,55	128,18	129,18	148,27	150,21	152,13	153,35	154,66	159,02	166,64	174,21	167,7	166,53	173,98	191,08	186,93	178,66	175,07	177,81	173,06	168,21	165,49	173,45	166,54
Outlet of Lac Euphrosine	111,77	115,96	125,46	127,09	128,09	147,18	149,12	151,04	152,26	153,57	157,93	165,55	173,12	166,61	165,44	172,89	189,99	185,84	177,57	173,98	176,72	171,97	167,12	164,4	172,36	165,45
Ballon R.	110,91	115,1	124,59	126,22	127,22	146,31	148,25	150,17	151,39	152,7	157,06	164,68	172,25	165,74	164,57	172,02	189,12	184,97	176,7	173,11	175,85	171,1	166,25	163,53	171,49	164,58
Sans Nom R.	110,45	114,64	124,13	125,76	126,76	145,85	147,79	149,71	150,93	152,24	156,6	164,22	171,79	165,28	164,11	171,56	188,66	184,51	176,24	172,65	175,39	170,64	165,79	163,07	171,03	164,12
S. Brook Ile aux Skuas	102,55	106,74	116,24	117,87	118,87	137,96	139,9	141,82	143,04	144,35	148,71	156,33	163,9	157,39	156,22	163,67	180,77	176,62	168,35	164,76	167,5	162,75	157,9	155,18	163,14	156,23
Brook 1 Ile du Port	97,533	101,72	111,22	112,85	113,85	132,94	134,88	136,8	138,02	139,33	143,69	151,31	158,88	152,37	151,2	158,65	175,75	171,6	163,33	159,74	162,48	157,73	152,88	150,16	158,12	151,21
Brook 2 Ile du Port	96,167	100,36	109,85	111,48	112,48	131,57	133,51	135,43	136,65	137,96	142,32	149,94	157,51	151	149,83	157,28	174,38	170,23	161,96	158,37	161,11	156,36	151,51	148,79	156,75	149,84
Brook 3 Ile du Port	94,893	99,083	108,58	110,21	111,21	130,3	132,24	134,16	135,38	136,69	141,05	148,67	156,24	149,73	148,56	156,01	173,11	168,96	160,69	157,1	159,84	155,09	150,24	147,52	155,48	148,57
Brook 4 Ile du Port	95,11	99,3	108,8	110,43	111,43	130,52	132,46	134,38	135,6	136,91	141,27	148,89	156,46	149,95	148,78	156,23	173,33	169,18	160,91	157,32	160,06	155,31	150,46	147,74	155,7	148,79
Val Froide R.	115,23	119,42	128,92	130,55	131,55	150,64	152,58	154,5	155,72	157,03	161,39	169,01	176,58	170,07	168,9	176,35	193,45	189,3	181,03	177,44	180,18	175,43	170,58	167,86	175,82	168,91
Val Travers R.	109,54	113,73	123,23	124,86	125,86	144,95	146,89	148,81	150,03	151,34	155,7	163,32	170,89	164,38	163,21	170,66	187,76	183,61	175,34	171,75	174,49	169,74	164,89	162,17	170,13	163,22
Brook N. of Port-Couvreux	96,641	100,83	110,33	111,96	112,96	132,05	133,99	135,91	137,13	138,44	142,8	150,42	157,99	151,48	150,31	157,76	174,86	170,71	162,44	158,85	161,59	156,84	151,99	149,27	157,23	150,32
Brook of Port-Couvreux cabin	97,575	101,77	111,26	112,89	113,89	132,98	134,92	136,84	138,06	139,37	143,73	151,35	158,92	152,41	151,24	158,69	175,79	171,64	163,37	159,78	162,52	157,77	152,92	150,2	158,16	151,25
Brook W end of Havre du Beau Temps	107,22	111,41	120,9	122,53	123,53	142,62	144,56	146,48	147,7	149,01	153,37	160,99	168,56	162,05	160,88	168,33	185,43	181,28	173,01	169,42	172,16	167,41	162,56	159,84	167,8	160,89
Anse Sablonneuse Brook	97,98	102,17	111,67	113,3	114,3	133,39	135,33	137,25	138,47	139,78	144,14	151,76	159,33	152,82	151,65	159,1	176,2	172,05	163,78	160,19	162,93	158,18	153,33	150,61	158,57	151,66
Port Kirk R.	93,035	97,225	106,72	108,35	109,35	128,44	130,38	132,3	133,52	134,83	139,19	146,81	154,38	147,87	146,7	154,15	171,25	167,1	158,83	155,24	157,98	153,23	148,38	145,66	153,62	146,71
Val de l'Ouest R.	88,88	93,07	102,57	104,2	105,2	124,29	126,23	128,15	129,37	130,68	135,04	142,66	150,23	143,72	142,55	150	167,1	162,95	154,68	151,09	153,83	149,08	144,23	141,51	149,47	142,56
Studer R.	81,767	85,957	95,453	97,083	98,083	117,17	119,11	121,03	122,25	123,56	127,92	135,54	143,11	136,6	135,43	142,88	159,98	155,83	147,56	143,97	146,71	141,96	137,11	134,39	142,35	135,44
Doute R.	77,722	81,912	91,408	93,038	94,038	113,13	115,07	116,99	118,21	119,52	123,88	131,5	139,07	132,56	131,39	138,84	155,94	151,79	143,52	139,93	142,67	137,92	133,07	130,35	138,31	131,4
Charbon R.	77,64	81,83	91,326	92,956	93,956	113,05	114,99	116,91	118,13	119,44	123,8	131,42	138,99	132,48	131,31	138,76	155,86	151,71	143,44	139,85	142,59	137,84	132,99	130,27	138,23	131,32
Sérail R.	71,852	76,042	85,538	87,168	88,168	107,26	109,2	111,12	112,34	113,65	118,01	125,63	133,2	126,69	125,52	132,97	150,07	145,92	137,65	134,06	136,8	132,05	127,2	124,48	132,44	125,53
Chasseurs R.	67,478	71,668	81,164	82,794	83,794	102,88	104,82	106,74	107,96	109,27	113,63	121,25	128,82	122,31	121,14	128,59	145,69	141,54	133,27	129,68	132,42	127,67	122,82	120,1	128,06	121,15
Nord R.	62,691	66,881	76,377	78,007	79,007	98,097	100,04	101,96	103,18	104,49	108,85	116,47	124,04	117,53	116,36	123,81	140,91	136,76	128,49	124,9	127,64	122,89	118,04	115,32	123,28	116,37
Pépins R.	59,919	64,109	73,605	75,235	76,235	95,325	97,265	99,185	100,41	101,72	106,08	113,7	121,27	114,76	113,59	121,04	138,14	133,99	125,72	122,13	124,87	120,12	115,27	112,55	120,51	113,6
Cataractes R.	57,57	61,76	71,256	72,886	73,886	92,976	94,916	96,836	98,056	99,366	103,73	111,35	118,92	112,41	111,24	118,69	135,79	131,64	123,37	119,78	122,52	117,77	112,92	110,2	118,16	111,25
Hautes Mares South Brook (Gorfous 1)	56,279	60,469	69,965	71,595	72,595	91,685	93,625	95,545	96,765	98,075	102,44	110,06	117,63	111,12	109,95	117,4	134,5	130,35	122,08	118,49	121,23	116,48	111,63	108,91	116,87	109,96
Hautes Mares North Brook (Gorfous 2)	55,89	60,08	69,576	71,206	72,206	91,296	93,236	95,156	96,376	97,686	102,05	109,67	117,24	110,73	109,56	117,01	134,11	129,96	121,69	118,1	120,84	116,09	111,24	108,52	116,48	109,57
Pointe des Cabanes Brook (Gorfous 4)	52,696	56,886	66,382	68,012	69,012	88,102	90,042	91,962	93,182	94,492	98,852	106,47	114,04	107,53	106,36	113,81	130,91	126,76	118,49	114,9	117,64	112,89	108,04	105,32	113,28	106,37
Cap Rouge Brook (Gorfous 5)	48,553	52,743	62,239	63,869	64,869	83,959	85,899	87,819	89,039	90,349	94,709	102,33	109,9	103,39	102,22	109,67	126,77	122,62	114,35	110,76	113,5	108,75	103,9	101,18	109,14	102,23
N. Cap Rouge Brook (Gorfous 6)	47,684	51,874	61,37	63	64	83,09	85,03	86,95	88,17	89,48	93,84	101,46	109,03	102,52	101,35	108,8	125,9	121,75	113,48	109,89	112,63	107,88	103,03	100,31	108,27	101,36
Cap de Rohan R.	39,332	43,522	53,018	54,648	55,648	74,738	76,678	78,598	79,818	81,128	85,488	93,108	100,68	94,168	92,998	100,45	117,55	113,4	105,13	101,54	104,28	99,528	94,678	91,958	99,918	93,008
Lac Marville Outlet	22,214	26,404	35,9	37,53	38,53	57,62	59,56	61,48	62,7	64,01	68,37	75,99	83,56	77,05	75,88	83,33	100,43	96,28	88,01	84,42	87,16	82,41	77,56	74,84	82,8	75,89
Manchots R.	12,395	16,585	26,081	27,711	28,711	47,801	49,741	51,661	52,881	54,191	58,551	66,171	73,741	67,231	66,061	73,511	90,611	86,461	78,191	74,601	77,341	72,591	67,741	65,021	72,981	66,071
Calcédoines R.	0	4,695	14,005	15,885	16,93	35,666	37,606	39,526	40,746	42,056	46,416	54,036	61,606	55,096	53,926	61,376	78,476	74,326	66,056	62,466	65,206	60,456	55,606	52,886	60,846	53,936
Bungay R.	4,695	0	9,755	11,635	12,86	31,932	33,872	35,792	37,012	38,322	42,682	50,302	57,872	51,362	50,192	57,642	74,742	70,592	62,322	58,732	61,472	56,722	51,872	49,152	57,112	50,202
Albatros R.	14,005	9,755	0	2,76	4,01	33,803	35,743	37,663	38,883	40,193	44,553	52,173	59,743	53,233	52,063	59,513	76,613	72,463	64,193	60,603	63,343	58,593	53,743	51,023	58,983	52,073
Norvégienne R.	15,885	11,635	2,76	0	2,259	35,329	37,269	39,189	40,409	41,719	46,079	53,699	61,269	54,759	53,589	61,039	78,139	73,989	65,719	62,129	64,869	60,119	55,269	52,549	60,509	53,599
Château R.	16,93	12,86	4,01	2,259	0	35,761	37,701	39,621	40,841	42,151	46,511	54,131	61,701	55,191	54,021	61,471	78,571	74,421	66,151	62,561	65,301	60,551	55,701	52,981	60,941	54,031
PAF Ferme R.	35,666	31,932	33,803	35,329	35,761	0	4,29	6,98	8,51	10,52	16,05	23,86	31,3	26,63	26,12	35,71	54,54	50,3	42,41	38,71	40,56	43,12	36,57	33,68	41,68	34,74
Borgne R.	37,606	33,872	35,743	37,269	37,701	4,29	0	2,89	4,33	6,66	12,52	20,33	27,77	23,14	22,63	32,38	51,56	47,33	39,44	35,74	37,73	40,37	34,25	31,36	39,36	32,42
Sud R.	39,526	35,792	37,663	39,189	39,621	6,98	2,89	0	1,47	3,86	10,04	17,84	25,22	20,67	20,17	30,04	49,36	45,13	37,24	33,56	35,71	38,35	33,07	30,18	38,17	31,23
Pointe de l'épave Brook	40,746	37,012	38,883	40,409	40,841	8,51	4,33	1,47	0	2,64	9,11	16,919	24,529	19,86	19,36	29,3	48,65	44,412	36,52	32,82	35	37,64	33,17	30,27	38,27	31,33
Américains R.	42,056	38,322	40,193	41,719	42,151	10,52	6,66	3,86	2,64	0	7,611	15,42	23,03	18,361	17,861	27,801	47,151	42,913	35,021	31,321	33,501	36,141	31,671	28,771	36,771	29,831
3 Lacs R.	46,416	42,682	44,553	46,079	46,511	16,05	12,52	10,04	9,11	7,611	0	8,3	15,91	11,73	11,04	22,4	42,811	38,04	30,15	26,22	29	31,64	28,36	25,46	33,46	26,52
Val d'Auge R.	54,036	50,302	52,173	53,699	54,131	23,86	20,33	17,84	16,919	15,42	8,3	0	8,1	12,32	11,82	24,54	44,95	40,1	32,29	28,36	30,7	33,34	33,16	30,26	38,26	31,32
Anse de St Malo R.	61,606	57,872	59,743	61,269	61,701	31,3	27,77	25,22	24,529	23,03	15,91	8,1	0	15,37	14,99	27,71	48,23	43,22	35,57	31,65	33,902	36,542	37,282	34,462	42,502	35,562
Basin downstream of Korrigans Lake	55,096	51,362	53,233	54,759	55,191	26,63	23,14	20,67	19,86	18,361	11,73	12,32	15,37	0	2,42	18,1	38,52	33,61	25,86	21,94	24,13	26,77	27,32	24,49	32,54	25,6
Baie de l'Observatoire R.	53,926	50,192	52,063	53,589	54,021	26,12	22,63	20,17	19,36	17,861	11,04	11,82	14,99	2,42	0	16,75	37,15	32,16	24,49	20,56	22,834	25,474	26,024	23,194	31,244	24,304
SW Brook on Bras Jules Laboureur	61,376	57,642	59,513	61,039	61,471	35,71	32,38	30,04	29,3	27,801	22,4	24,54	27,71	18,1	16,75	0	28,89	22,92	15,43	13,34	18,38	21,29	28,59	27,36	35,4	28,46
Grisanche R.	78,476	74,742	76,613	78,139	78,571	54,54	51,56	49,36	48,65	47,151	42,811	44,95	48,23	38,52	37,15	28,89	0	4,97	18,44	21,12	25,982	29,082	34,802	39,732	48,132	41,102
Lac des Fougères Outlet	74,326	70,592	72,463	73,989	74,421	50,3	47,33	45,13	44,412	42,913	38,04	40,1	43,22	33,61	32,16	22,92	4,97	0	13,47	16,15	21,77	24,87	30,59	35,52	43,92	36,89
Lac d'Armor Basin	66,056	62,322	64,193	65,719	66,151	42,41	39,44	37,24	36,52	35,021	30,15	32,29	35,57	25,86	24,49	15,43	18,44	13,47	0	7,44	13,37	16,42	22,14	27,24	36,07	29,01
Anse J. Bourcart Brook	62,466	58,732	60,603	62,129	62,561	38,71	35,74	33,56	32,82	31,321	26,22	28,36	31,65	21,94	20,56	13,34	21,12	16,15	7,44	0	11,01	12	12,73	22,87	31,26	24,57
Ravin du Charbon R.	65,206	61,472	63,343	64,869	65,301	40,56	37,73	35,71	35	33,501	29	30,7	33,902	24,13	22,834	18,38	25,982	21,77	13,37	11,01	0	4,54	10,61	16,2	24,29	17,78
Vallée des Neiges Basin	60,456	56,722	58,593	60,119	60,551	43,12	40,37	38,35	37,64	36,141	31,64	33,34	36,542	26,77	25,474	21,29	29,082	24,87	16,42	12	4,54	0	6,16	11,55	19,88	13,17
Acaena R.	55,606	51,872	53,743	55,269	55,701	36,57	34,25	33,07	33,17	31,671	28,36	33,16	37,282	27,32	26,024	28,59	34,802	30,59	22,14	12,73	10,61	6,16	0	6,55	15,08	8,38
Vallée de la Planchette R.	52,886	49,152	51,023	52,549	52,981	33,68	31,36	30,18	30,27	28,771	25,46	30,26	34,462	24,49	23,194	27,36	39,732	35,52	27,24	22,87	16,2	11,55	6,55	0	9,09	2,67
Val Raide Brook	60,846	57,112	58,983	60,509	60,941	41,68	39,36	38,17	38,27	36,771	33,46	38,26	42,502	32,54	31,244	35,4	48,132	43,92	36,07	31,26	24,29	19,88	15,08	9,09	0	7,07
Mouettes R.	53,936	50,202	52,073	53,599	54,031	34,74	32,42	31,23	31,33	29,831	26,52	31,32	35,562	25,6	24,304	28,46	41,102	36,89	29,01	24,57	17,78	13,17	8,38	2,67	7,07	0
Macaronis R.	36,416	32,682	34,553	36,079	36,511	29,5	31,15	32,42	33,59	34,82	35,97	43,25	49,462	42,199	40,903	47,29	62,362	58,15	50,3	46,134	48,83	44,28	39,16	36,26	44,46	37,52
Vallée Phonolite Basin	48,576	44,842	46,713	48,239	48,671	41,8	43,45	44,72	45,9	47,12	48,28	55,55	61,762	54,5	53,204	59,59	74,673	70,461	62,6	58,44	61,142	56,54	51,68	48,65	56,75	49,87
Val du Levant R.	56,296	52,562	54,433	55,959	56,391	49,46	51,11	52,38	53,55	54,78	55,93	63,21	69,722	62,46	61,164	67,514	82,595	78,383	70,525	66,362	69,064	64,51	59,65	56,578	64,678	57,801
Anse de la Canicule R.	83,036	79,302	81,173	82,699	83,131	79,031	80,81	82,163	83,205	84,253	84,863	92,381	99,168	91,905	90,782	96,95	112,22	107,81	99,664	95,93	98,583	93,985	89,252	86,117	94,308	87,32
Crête des Laves R.	85,156	81,422	83,293	84,819	85,251	81,151	82,93	84,283	85,325	86,373	86,983	94,501	101,29	94,025	92,902	99,07	114,34	109,93	101,78	98,05	100,7	96,105	91,372	88,237	96,428	89,44
Halage des Swains R.	96,146	92,412	94,283	95,809	96,241	92,141	93,92	95,273	96,315	97,363	97,973	105,49	112,28	105,02	103,89	110,06	125,33	120,92	112,77	109,04	111,69	107,1	102,36	99,227	107,42	100,43
Northern arm of Anse aux Ecueils Brook	105,27	101,54	103,41	104,93	105,36	101,26	103,04	104,4	105,44	106,49	107,1	114,61	121,4	114,14	113,02	119,18	134,45	130,04	121,9	118,16	120,82	116,22	111,49	108,35	116,54	109,55
La Clarée Basin: Hermance Lake & Galets R.	104,53	100,79	102,66	104,19	104,62	100,52	102,3	103,65	104,7	105,74	106,35	113,87	120,66	113,4	112,27	118,44	133,71	129,3	121,15	117,42	120,07	115,48	110,74	107,61	115,8	108,81
Olsen R.	102,63	98,892	100,76	102,29	102,72	98,621	100,4	101,75	102,8	103,84	104,45	111,97	118,76	111,5	110,37	116,54	131,81	127,4	119,25	115,52	118,17	113,58	108,84	105,71	113,9	106,91
Vallée du Radioleine R.	98,326	94,592	96,463	97,989	98,421	94,321	96,1	97,453	98,495	99,543	100,15	107,67	114,46	107,2	106,07	112,24	127,51	123,1	114,95	111,22	113,87	109,28	104,54	101,41	109,6	102,61
Val des Skuas R.	96,436	92,702	94,573	96,099	96,531	92,431	94,21	95,563	96,605	97,653	98,263	105,78	112,57	105,31	104,18	110,35	125,62	121,21	113,06	109,33	111,98	107,39	102,65	99,517	107,71	100,72
Doigt de Sainte-Anne R.	115,71	111,97	113,84	115,37	115,8	111,7	113,48	114,83	115,88	116,92	117,53	125,05	131,84	124,58	123,45	129,62	144,89	140,48	132,33	128,6	131,25	126,66	121,92	118,79	126,98	119,99
Plaine de Dante R.	117,97	114,23	116,1	117,63	118,06	113,96	115,74	117,09	118,14	119,18	119,79	127,31	134,1	126,84	125,71	131,88	147,15	142,74	134,59	130,86	133,51	128,92	124,18	121,05	129,24	122,25
Mont Ventoux R.	121,34	125,53	135,03	136,66	137,66	156,75	158,69	160,61	161,83	163,14	167,5	175,12	182,69	176,18	175,01	182,46	199,56	195,41	187,14	183,55	186,29	181,54	176,69	173,97	181,93	175,02
Mont Sinaï R.	117,36	121,55	131,05	132,68	133,68	152,77	154,71	156,63	157,85	159,16	163,52	171,14	178,71	172,2	171,03	178,48	195,58	191,43	183,16	179,57	182,31	177,56	172,71	169,99	177,95	171,04
Bastille R.	109,46	113,65	123,15	124,78	125,78	144,87	146,81	148,73	149,95	151,26	155,62	163,24	170,81	164,3	163,13	170,58	187,68	183,53	175,26	171,67	174,41	169,66	164,81	162,09	170,05	163,14
Lac Mariot outlet	132,19	128,45	130,32	131,85	132,28	128,18	129,96	131,31	132,36	133,4	134,01	141,53	148,32	141,06	139,93	146,1	161,37	156,96	148,81	145,08	147,73	143,14	138,4	135,27	143,46	136,47
Plaine d'Ampère R.	133,46	129,72	131,59	133,12	133,55	129,45	131,23	132,58	133,63	134,67	135,28	142,8	149,59	142,33	141,2	147,37	162,64	158,23	150,08	146,35	149	144,41	139,67	136,54	144,73	137,74
Portes Noires R.	141,24	137,5	139,37	140,9	141,33	137,23	139,01	140,36	141,41	142,45	143,06	150,58	157,37	150,11	148,98	155,15	170,42	166,01	157,86	154,13	156,78	152,19	147,45	144,32	152,51	145,52
Contacts R.	142,47	138,73	140,6	142,13	142,56	138,46	140,24	141,59	142,64	143,68	144,29	151,81	158,6	151,34	150,21	156,38	171,65	167,24	159,09	155,36	158,01	153,42	148,68	145,55	153,74	146,75
Mouche R.	137,93	134,19	136,06	137,59	138,02	133,92	135,7	137,05	138,1	139,14	139,75	147,27	154,06	146,8	145,67	151,84	167,11	162,7	154,55	150,82	153,47	148,88	144,14	141,01	149,2	142,21
Larmor R.	131,94	128,2	130,07	131,6	132,03	127,93	129,71	131,06	132,11	133,15	133,76	141,28	148,07	140,81	139,68	145,85	161,12	156,71	148,56	144,83	147,48	142,89	138,15	135,02	143,21	136,22
Plage Jaune R.	138,16	134,42	136,29	137,82	138,25	134,15	135,93	137,28	138,33	139,37	139,98	147,5	154,29	147,03	145,9	152,07	167,34	162,93	154,78	151,05	153,7	149,11	144,37	141,24	149,43	142,44
Sables R.	145,01	141,27	143,14	144,67	145,1	141	142,78	144,13	145,18	146,22	146,83	154,35	161,14	153,88	152,75	158,92	174,19	169,78	161,63	157,9	160,55	155,96	151,22	148,09	156,28	149,29

Patch names	Macaronis R.	Vallée Phonolite Basin	Val du Levant R.	Anse de la Canicule R.	Crête des Laves R.	Halage des Swains R.	Northern arm of Anse aux Ecueils Brook	La Clarée Basin: Hermance Lake & Galets R	Olsen R.	Vallée du Radioleine R.	Val des Skuas R.	Doigt de Sainte-Anne R.	Plaine de Dante R.	Mont Ventoux R.	Mont Sinaï R.	Bastille R.	Lac Mariot outlet	Plaine d'Ampère R.	Portes Noires R.	Contacts R.	Mouche R.	Larmor R.	Plage Jaune R.	Sables R.
Entrelacs R.	159,8	171,96	179,68	206,42	208,54	219,53	228,65	227,91	226,01	221,71	219,82	239,09	241,35	2,67	8,93	44,56	255,57	256,84	264,62	265,85	261,31	255,32	261,54	268,39
Baie Chanzy	153,36	165,52	173,24	199,98	202,1	213,09	222,22	221,47	219,57	215,27	213,38	232,65	234,91	22,716	18,586	38,32	249,13	250,4	258,18	259,41	254,87	248,88	255,1	261,95
Déversoir du Lac de la Croix du Sud	149,02	161,18	168,9	195,64	197,76	208,75	217,87	217,13	215,23	210,93	209,04	228,31	230,57	22,916	18,786	36,424	244,79	246,06	253,84	255,07	250,53	244,54	250,76	257,61
Outlet of Lac Euphrosine	147,93	160,09	167,81	194,55	196,67	207,66	216,78	216,04	214,14	209,84	207,95	227,22	229,48	30,256	26,126	40,51	243,7	244,97	252,75	253,98	249,44	243,45	249,67	256,52
Ballon R.	147,06	159,22	166,94	193,68	195,8	206,79	215,92	215,17	213,27	208,97	207,08	226,35	228,61	29,636	25,506	39,28	242,83	244,1	251,88	253,11	248,57	242,58	248,8	255,65
Sans Nom R.	146,6	158,76	166,48	193,22	195,34	206,33	215,46	214,71	212,81	208,51	206,62	225,89	228,15	29,056	24,926	38,881	242,37	243,64	251,42	252,65	248,11	242,12	248,34	255,19
S. Brook Ile aux Skuas	138,71	150,87	158,59	185,33	187,45	198,44	207,56	206,82	204,92	200,62	198,73	218	220,26	28,836	24,706	38,98	234,48	235,75	243,53	244,76	240,22	234,23	240,45	247,3
Brook 1 Ile du Port	133,69	145,85	153,57	180,31	182,43	193,42	202,54	201,8	199,9	195,6	193,71	212,98	215,24	29,849	25,868	28,88	229,46	230,73	238,51	239,74	235,2	229,21	235,43	242,28
Brook 2 Ile du Port	132,32	144,48	152,2	178,94	181,06	192,05	201,18	200,43	198,53	194,23	192,34	211,61	213,87	31,259	27,278	27,35	228,09	229,36	237,14	238,37	233,83	227,84	234,06	240,91
Brook 3 Ile du Port	131,05	143,21	150,93	177,67	179,79	190,78	199,9	199,16	197,26	192,96	191,07	210,34	212,6	32,949	28,968	24,78	226,82	228,09	235,87	237,1	232,56	226,57	232,79	239,64
Brook 4 Ile du Port	131,27	143,43	151,15	177,89	180,01	191	200,12	199,38	197,48	193,18	191,29	210,56	212,82	33,73	29,749	24,68	227,04	228,31	236,09	237,32	232,78	226,79	233,01	239,86
Val Froide R.	151,39	163,55	171,27	198,01	200,13	211,12	220,24	219,5	217,6	213,3	211,41	230,68	232,94	36,929	32,948	47,62	247,16	248,43	256,21	257,44	252,9	246,91	253,13	259,98
Val Travers R.	145,7	157,86	165,58	192,32	194,44	205,43	214,55	213,81	211,91	207,61	205,72	224,99	227,25	31,779	27,798	41,82	241,47	242,74	250,52	251,75	247,21	241,22	247,44	254,29
Brook N. of Port-Couvreux	132,8	144,96	152,68	179,42	181,54	192,53	201,65	200,91	199,01	194,71	192,82	212,09	214,35	42,019	38,038	36,89	228,57	229,84	237,62	238,85	234,31	228,32	234,54	241,39
Brook of Port-Couvreux cabin	133,73	145,89	153,61	180,35	182,47	193,46	202,58	201,84	199,94	195,64	193,75	213,02	215,28	42,829	38,848	36,99	229,5	230,77	238,55	239,78	235,24	229,25	235,47	242,32
Brook W end of Havre du Beau Temps	143,37	155,53	163,25	189,99	192,11	203,1	212,23	211,48	209,58	205,28	203,39	222,66	224,92	53,279	49,298	48,18	239,14	240,41	248,19	249,42	244,88	238,89	245,11	251,96
Anse Sablonneuse Brook	134,14	146,3	154,02	180,76	182,88	193,87	202,99	202,25	200,35	196,05	194,16	213,43	215,69	44,259	40,278	38,75	229,91	231,18	238,96	240,19	235,65	229,66	235,88	242,73
Port Kirk R.	129,19	141,35	149,07	175,81	177,93	188,92	198,04	197,3	195,4	191,1	189,21	208,48	210,74	45,429	41,448	37,29	224,96	226,23	234,01	235,24	230,7	224,71	230,93	237,78
Val de l'Ouest R.	125,04	137,2	144,92	171,66	173,78	184,77	193,89	193,15	191,25	186,95	185,06	204,33	206,59	45,509	41,528	35,25	220,81	222,08	229,86	231,09	226,55	220,56	226,78	233,63
Studer R.	117,92	130,08	137,8	164,54	166,66	177,65	186,78	186,03	184,13	179,83	177,94	197,21	199,47	43,639	39,658	31,85	213,69	214,96	222,74	223,97	219,43	213,44	219,66	226,51
Doute R.	113,88	126,04	133,76	160,5	162,62	173,61	182,73	181,99	180,09	175,79	173,9	193,17	195,43	47,699	43,718	36,08	209,65	210,92	218,7	219,93	215,39	209,4	215,62	222,47
Charbon R.	113,8	125,96	133,68	160,42	162,54	173,53	182,65	181,91	180,01	175,71	173,82	193,09	195,35	47,819	43,838	36,09	209,57	210,84	218,62	219,85	215,31	209,32	215,54	222,39
Sérail R.	108,01	120,17	127,89	154,63	156,75	167,74	176,86	176,12	174,22	169,92	168,03	187,3	189,56	51,659	47,678	40	203,78	205,05	212,83	214,06	209,52	203,53	209,75	216,6
Chasseurs R.	103,63	115,79	123,51	150,25	152,37	163,36	172,49	171,74	169,84	165,54	163,65	182,92	185,18	56,359	52,378	44,18	199,4	200,67	208,45	209,68	205,14	199,15	205,37	212,22
Nord R.	98,847	111,01	118,73	145,47	147,59	158,58	167,7	166,96	165,06	160,76	158,87	178,14	180,4	69,419	65,438	57,48	194,62	195,89	203,67	204,9	200,36	194,37	200,59	207,44
Pépins R.	96,075	108,24	115,96	142,7	144,82	155,81	164,93	164,19	162,29	157,99	156,1	175,37	177,63	70,709	66,728	59,05	191,85	193,12	200,9	202,13	197,59	191,6	197,82	204,67
Cataractes R.	93,726	105,89	113,61	140,35	142,47	153,46	162,58	161,84	159,94	155,64	153,75	173,02	175,28	70,629	66,648	58,44	189,5	190,77	198,55	199,78	195,24	189,25	195,47	202,32
Hautes Mares South Brook (Gorfous 1)	92,435	104,6	112,32	139,06	141,18	152,17	161,29	160,55	158,65	154,35	152,46	171,73	173,99	70,739	66,758	58,67	188,21	189,48	197,26	198,49	193,95	187,96	194,18	201,03
Hautes Mares North Brook (Gorfous 2)	92,046	104,21	111,93	138,67	140,79	151,78	160,9	160,16	158,26	153,96	152,07	171,34	173,6	70,829	66,848	58,66	187,82	189,09	196,87	198,1	193,56	187,57	193,79	200,64
Pointe des Cabanes Brook (Gorfous 4)	88,852	101,01	108,73	135,47	137,59	148,58	157,71	156,96	155,06	150,76	148,87	168,14	170,4	70,589	66,608	58,64	184,62	185,89	193,67	194,9	190,36	184,37	190,59	197,44
Cap Rouge Brook (Gorfous 5)	84,709	96,869	104,59	131,33	133,45	144,44	153,56	152,82	150,92	146,62	144,73	164	166,26	73,479	69,498	61,366	180,48	181,75	189,53	190,76	186,22	180,23	186,45	193,3
N. Cap Rouge Brook (Gorfous 6)	83,84	96	103,72	130,46	132,58	143,57	152,69	151,95	150,05	145,75	143,86	163,13	165,39	74,129	70,148	62,295	179,61	180,88	188,66	189,89	185,35	179,36	185,58	192,43
Cap de Rohan R.	75,488	87,648	95,368	122,11	124,23	135,22	144,34	143,6	141,7	137,4	135,51	154,78	157,04	82,179	78,198	70,127	171,26	172,53	180,31	181,54	177	171,01	177,23	184,08
Lac Marville Outlet	58,37	70,53	78,25	104,99	107,11	118,1	127,22	126,48	124,58	120,28	118,39	137,66	139,92	99,879	95,898	87,995	154,14	155,41	163,19	164,42	159,88	153,89	160,11	166,96
Manchots R.	48,551	60,711	68,431	95,171	97,291	108,28	117,4	116,66	114,76	110,46	108,57	127,84	130,1	108,85	104,87	96,965	144,32	145,59	153,37	154,6	150,06	144,07	150,29	157,14
Calcédoines R.	36,416	48,576	56,296	83,036	85,156	96,146	105,27	104,53	102,63	98,326	96,436	115,71	117,97	121,34	117,36	109,46	132,19	133,46	141,24	142,47	137,93	131,94	138,16	145,01
Bungay R.	32,682	44,842	52,562	79,302	81,422	92,412	101,54	100,79	98,892	94,592	92,702	111,97	114,23	125,53	121,55	113,65	128,45	129,72	137,5	138,73	134,19	128,2	134,42	141,27
Albatros R.	34,553	46,713	54,433	81,173	83,293	94,283	103,41	102,66	100,76	96,463	94,573	113,84	116,1	135,03	131,05	123,15	130,32	131,59	139,37	140,6	136,06	130,07	136,29	143,14
Norvégienne R.	36,079	48,239	55,959	82,699	84,819	95,809	104,93	104,19	102,29	97,989	96,099	115,37	117,63	136,66	132,68	124,78	131,85	133,12	140,9	142,13	137,59	131,6	137,82	144,67
Château R.	36,511	48,671	56,391	83,131	85,251	96,241	105,36	104,62	102,72	98,421	96,531	115,8	118,06	137,66	133,68	125,78	132,28	133,55	141,33	142,56	138,02	132,03	138,25	145,1
PAF Ferme R.	29,5	41,8	49,46	79,031	81,151	92,141	101,26	100,52	98,621	94,321	92,431	111,7	113,96	156,75	152,77	144,87	128,18	129,45	137,23	138,46	133,92	127,93	134,15	141
Borgne R.	31,15	43,45	51,11	80,81	82,93	93,92	103,04	102,3	100,4	96,1	94,21	113,48	115,74	158,69	154,71	146,81	129,96	131,23	139,01	140,24	135,7	129,71	135,93	142,78
Sud R.	32,42	44,72	52,38	82,163	84,283	95,273	104,4	103,65	101,75	97,453	95,563	114,83	117,09	160,61	156,63	148,73	131,31	132,58	140,36	141,59	137,05	131,06	137,28	144,13
Pointe de l'épave Brook	33,59	45,9	53,55	83,205	85,325	96,315	105,44	104,7	102,8	98,495	96,605	115,88	118,14	161,83	157,85	149,95	132,36	133,63	141,41	142,64	138,1	132,11	138,33	145,18
Américains R.	34,82	47,12	54,78	84,253	86,373	97,363	106,49	105,74	103,84	99,543	97,653	116,92	119,18	163,14	159,16	151,26	133,4	134,67	142,45	143,68	139,14	133,15	139,37	146,22
3 Lacs R.	35,97	48,28	55,93	84,863	86,983	97,973	107,1	106,35	104,45	100,15	98,263	117,53	119,79	167,5	163,52	155,62	134,01	135,28	143,06	144,29	139,75	133,76	139,98	146,83
Val d'Auge R.	43,25	55,55	63,21	92,381	94,501	105,49	114,61	113,87	111,97	107,67	105,78	125,05	127,31	175,12	171,14	163,24	141,53	142,8	150,58	151,81	147,27	141,28	147,5	154,35
Anse de St Malo R.	49,462	61,762	69,722	99,168	101,29	112,28	121,4	120,66	118,76	114,46	112,57	131,84	134,1	182,69	178,71	170,81	148,32	149,59	157,37	158,6	154,06	148,07	154,29	161,14
Basin downstream of Korrigans Lake	42,199	54,5	62,46	91,905	94,025	105,02	114,14	113,4	111,5	107,2	105,31	124,58	126,84	176,18	172,2	164,3	141,06	142,33	150,11	151,34	146,8	140,81	147,03	153,88
Baie de l'Observatoire R.	40,903	53,204	61,164	90,782	92,902	103,89	113,02	112,27	110,37	106,07	104,18	123,45	125,71	175,01	171,03	163,13	139,93	141,2	148,98	150,21	145,67	139,68	145,9	152,75
SW Brook on Bras Jules Laboureur	47,29	59,59	67,514	96,95	99,07	110,06	119,18	118,44	116,54	112,24	110,35	129,62	131,88	182,46	178,48	170,58	146,1	147,37	155,15	156,38	151,84	145,85	152,07	158,92
Grisanche R.	62,362	74,673	82,595	112,22	114,34	125,33	134,45	133,71	131,81	127,51	125,62	144,89	147,15	199,56	195,58	187,68	161,37	162,64	170,42	171,65	167,11	161,12	167,34	174,19
Lac des Fougères Outlet	58,15	70,461	78,383	107,81	109,93	120,92	130,04	129,3	127,4	123,1	121,21	140,48	142,74	195,41	191,43	183,53	156,96	158,23	166,01	167,24	162,7	156,71	162,93	169,78
Lac d'Armor Basin	50,3	62,6	70,525	99,664	101,78	112,77	121,9	121,15	119,25	114,95	113,06	132,33	134,59	187,14	183,16	175,26	148,81	150,08	157,86	159,09	154,55	148,56	154,78	161,63
Anse J. Bourcart Brook	46,134	58,44	66,362	95,93	98,05	109,04	118,16	117,42	115,52	111,22	109,33	128,6	130,86	183,55	179,57	171,67	145,08	146,35	154,13	155,36	150,82	144,83	151,05	157,9
Ravin du Charbon R.	48,83	61,142	69,064	98,583	100,7	111,69	120,82	120,07	118,17	113,87	111,98	131,25	133,51	186,29	182,31	174,41	147,73	149	156,78	158,01	153,47	147,48	153,7	160,55
Vallée des Neiges Basin	44,28	56,54	64,51	93,985	96,105	107,1	116,22	115,48	113,58	109,28	107,39	126,66	128,92	181,54	177,56	169,66	143,14	144,41	152,19	153,42	148,88	142,89	149,11	155,96
Acaena R.	39,16	51,68	59,65	89,252	91,372	102,36	111,49	110,74	108,84	104,54	102,65	121,92	124,18	176,69	172,71	164,81	138,4	139,67	147,45	148,68	144,14	138,15	144,37	151,22
Vallée de la Planchette R.	36,26	48,65	56,578	86,117	88,237	99,227	108,35	107,61	105,71	101,41	99,517	118,79	121,05	173,97	169,99	162,09	135,27	136,54	144,32	145,55	141,01	135,02	141,24	148,09
Val Raide Brook	44,46	56,75	64,678	94,308	96,428	107,42	116,54	115,8	113,9	109,6	107,71	126,98	129,24	181,93	177,95	170,05	143,46	144,73	152,51	153,74	149,2	143,21	149,43	156,28
Mouettes R.	37,52	49,87	57,801	87,32	89,44	100,43	109,55	108,81	106,91	102,61	100,72	119,99	122,25	175,02	171,04	163,14	136,47	137,74	145,52	146,75	142,21	136,22	142,44	149,29
Macaronis R.	0	12,36	20,168	49,401	51,521	62,511	71,634	70,891	68,991	64,691	62,801	82,071	84,331	157,5	153,52	145,62	98,551	99,821	107,6	108,83	104,29	98,301	104,52	111,37
Vallée Phonolite Basin	12,36	0	20,68	47,282	49,402	60,39	69,507	68,77	66,87	62,56	60,67	79,94	82,206	169,66	165,68	157,78	96,426	97,695	105,48	106,71	102,17	96,177	102,4	109,25
Val du Levant R.	20,168	20,68	0	30,131	32,251	43,239	52,356	51,619	49,719	45,409	44,54	62,789	65,055	177,38	173,4	165,5	79,275	80,544	88,329	89,559	85,019	79,026	85,249	92,099
Anse de la Canicule R.	49,401	47,282	30,131	0	2,847	13,837	22,947	22,52	20,43	16,19	19,163	37,412	39,678	204,12	200,14	192,24	53,898	55,167	62,952	64,182	59,642	53,649	59,872	66,722
Crête des Laves R.	51,521	49,402	32,251	2,847	0	11,242	20,352	19,94	17,89	13,52	16,937	35,186	37,452	206,24	202,26	194,36	51,672	52,941	60,726	61,956	57,416	51,423	57,646	64,496
Halage des Swains R.	62,511	60,39	43,239	13,837	11,242	0	11,671	14,99	13,17	8,91	22,194	40,443	42,709	217,23	213,25	205,35	56,929	58,198	65,983	67,213	62,673	56,68	62,903	69,753
Northern arm of Anse aux Ecueils Brook	71,634	69,507	52,356	22,947	20,352	11,671	0	16,05	14,3	10,3	29,299	47,548	49,814	226,35	222,37	214,47	64,034	65,303	73,088	74,318	69,778	63,785	70,008	76,858
La Clarée Basin: Hermance Lake & Galets R.	70,891	68,77	51,619	22,52	19,94	14,99	16,05	0	2,57	6,41	27,79	46,009	48,275	225,61	221,63	213,73	62,495	63,764	71,549	72,779	68,239	62,246	68,469	75,319
Olsen R.	68,991	66,87	49,719	20,43	17,89	13,17	14,3	2,57	0	4,44	25,74	44,084	46,35	223,71	219,73	211,83	60,57	61,839	69,624	70,854	66,314	60,321	66,544	73,394
Vallée du Radioleine R.	64,691	62,56	45,409	16,19	13,52	8,91	10,3	6,41	4,44	0	21,43	39,703	41,969	219,41	215,43	207,53	56,189	57,458	65,243	66,473	61,933	55,94	62,163	69,013
Val des Skuas R.	62,801	60,67	44,54	19,163	16,937	22,194	29,299	27,79	25,74	21,43	0	19,73	22,19	217,52	213,54	205,64	36,37	37,64	45,47	47,58	43,03	36,97	43,22	50,07
Doigt de Sainte-Anne R.	82,071	79,94	62,789	37,412	35,186	40,443	47,548	46,009	44,084	39,703	19,73	0	2,44	236,79	232,81	224,91	16,91	19,34	27,17	32,6	28,09	22,49	29,39	36,29
Plaine de Dante R.	84,331	82,206	65,055	39,678	37,452	42,709	49,814	48,275	46,35	41,969	22,19	2,44	0	239,05	235,07	227,17	14,71	17,94	25,71	32,67	28,2	22,62	29,62	36,51
Mont Ventoux R.	157,5	169,66	177,38	204,12	206,24	217,23	226,35	225,61	223,71	219,41	217,52	236,79	239,05	0	6,89	42,45	253,27	254,54	262,32	263,55	259,01	253,02	259,24	266,09
Mont Sinaï R.	153,52	165,68	173,4	200,14	202,26	213,25	222,37	221,63	219,73	215,43	213,54	232,81	235,07	6,89	0	38,04	249,29	250,56	258,34	259,57	255,03	249,04	255,26	262,11
Bastille R.	145,62	157,78	165,5	192,24	194,36	205,35	214,47	213,73	211,83	207,53	205,64	224,91	227,17	42,45	38,04	0	241,39	242,66	250,44	251,67	247,13	241,14	247,36	254,21
Lac Mariot outlet	98,551	96,426	79,275	53,898	51,672	56,929	64,034	62,495	60,57	56,189	36,37	16,91	14,71	253,27	249,29	241,39	0	28,22	36,04	43,93	39,511	33,95	41,06	47,95
Plaine d'Ampère R.	99,821	97,695	80,544	55,167	52,941	58,198	65,303	63,764	61,839	57,458	37,64	19,34	17,94	254,54	250,56	242,66	28,22	0	9,6	34,27	29,77	24,34	32,22	39,08
Portes Noires R.	107,6	105,48	88,329	62,952	60,726	65,983	73,088	71,549	69,624	65,243	45,47	27,17	25,71	262,32	258,34	250,44	36,04	9,6	0	41,755	37,255	31,825	39,705	46,565
Contacts R.	108,83	106,71	89,559	64,182	61,956	67,213	74,318	72,779	70,854	66,473	47,58	32,6	32,67	263,55	259,57	251,67	43,93	34,27	41,755	0	5,04	14,58	22,81	29,867
Mouche R.	104,29	102,17	85,019	59,642	57,416	62,673	69,778	68,239	66,314	61,933	43,03	28,09	28,2	259,01	255,03	247,13	39,511	29,77	37,255	5,04	0	9,98	18,21	25,26
Larmor R.	98,301	96,177	79,026	53,649	51,423	56,68	63,785	62,246	60,321	55,94	36,97	22,49	22,62	253,02	249,04	241,14	33,95	24,34	31,825	14,58	9,98	0	8,8	15,86
Plage Jaune R.	104,52	102,4	85,249	59,872	57,646	62,903	70,008	68,469	66,544	62,163	43,22	29,39	29,62	259,24	255,26	247,36	41,06	32,22	39,705	22,81	18,21	8,8	0	7,43
Sables R.	111,37	109,25	92,099	66,722	64,496	69,753	76,858	75,319	73,394	69,013	50,07	36,29	36,51	266,09	262,11	254,21	47,95	39,08	46,565	29,867	25,26	15,86	7,43	0

Labonne et al. 2013, Appendix 2C: Distance matrix between hydrographic systems calculated with a 5 km buffer
